# Dynamic Evolution of α-Gliadin Prolamin Gene Family in Homeologous Genomes of Hexaploid Wheat

**DOI:** 10.1038/s41598-018-23570-5

**Published:** 2018-03-26

**Authors:** Naxin Huo, Tingting Zhu, Susan Altenbach, Lingli Dong, Yi Wang, Toni Mohr, Zhiyong Liu, Jan Dvorak, Ming-Cheng Luo, Yong Q. Gu

**Affiliations:** 1 United States Department of Agriculture-Agricultural Research Service, Western Regional Research Center, Albany, California 94710 USA; 2 Department of Plant Sciences, University of California, Davis, CA 95616 USA; 3 State Key Laboratory of Plant Cell and Chromosome Engineering, Institute of Genetics and Developmental Biology, Chinese Academy of Sciences, Beijing, 100101 China

## Abstract

Wheat *Gli-2* loci encode complex groups of α-gliadin prolamins that are important for breadmaking, but also major triggers of celiac disease (CD). Elucidation of α-gliadin evolution provides knowledge to produce wheat with better end-use properties and reduced immunogenic potential. The *Gli-2* loci contain a large number of tandemly duplicated genes and highly repetitive DNA, making sequence assembly of their genomic regions challenging. Here, we constructed high-quality sequences spanning the three wheat homeologous α-gliadin loci by aligning PacBio-based sequence contigs with BioNano genome maps. A total of 47 α-gliadin genes were identified with only 26 encoding intact full-length protein products. Analyses of α-gliadin loci and phylogenetic tree reconstruction indicate significant duplications of α-gliadin genes in the last ~2.5 million years after the divergence of the A, B and D genomes, supporting its rapid lineage-independent expansion in different Triticeae genomes. We showed that dramatic divergence in expression of α-gliadin genes could not be attributed to sequence variations in the promoter regions. The study also provided insights into the evolution of CD epitopes and identified a single indel event in the hexaploid wheat D genome that likely resulted in the generation of the highly toxic 33-mer CD epitope.

## Introduction

Polyploidization, an evolutionary process resulting in more than one genome per cell, has played a significant role in the evolutionary history of plants, particularly in agriculturally important crops^1–4^. One of the best examples is bread wheat, which is an annual species in the tribe of the grass family *Poaceae*, and one of the most widely cultivated and most important staple crops worldwide. Bread wheat is an allohexaploid species (*Triticum aestivum* L. 2n = 6 × = 42), consisting of three sets of highly related homeologous genomes (A, B, and D), each of which contains seven pairs of chromosomes. The origin and evolution of hexaploid wheat has been well documented through extensive genetic, cytogenetic, taxonomical, and phylogenomic studies^5,6^. The allohexaploid wheat species is believed to have originated from two independent polyploidization events. The first event, occurring 0.36 to 0.5 million years ago, involved the hybridization of two diploid progenitors, an ancestor of *Triticum urartu* (2n = 2 × = 14, genome AA) and an unconfirmed species (BB genome) related to *Aegilops speltoides* (2n = 2 × = 14, genome SS), which resulted in cultivated allotetraploid emmer wheat (*T. turgidum* ssp. *dicoccum*, 2n = 4 × = 28, genomes AABB)^7,8^. In the second event, which occurred 8,000~10,000 years ago, an ancestor of the diploid *Aegilops tauschii* (DD genome) hybridized with the allotetraploid to form a hexaploid wheat (2n = 6 × = 42)^6^. Recent phylogenomics studies using available genome sequence resources from wheat and its wild diploid ancestor species have provided new insights into the origin and relationship of the wheat A, B and D subgenomes^9^. It has been hypothesized that the D subgenome in hexaploid wheat resulted from a complex history of single or possibly multiple rounds of hybridization between ancestral A and B diploid species^9,10^.

Because of its relatively recent speciation, wheat represents an excellent system to study evolutionary events that occur in genomes shortly after allopolyploidization. In newly formed allopolyploids following the hybridization of two divergent species, various molecular processes including genetic, epigenetic, and structural subgenome modifications must take place, presumably to couple with the “genome shock” and facilitate the coordination of functions of multiple genomes in a single cell^3,5^. Although the mechanisms underlying these processes are still not clear, a number of investigations have documented active occurrence of the elimination of coding and noncoding DNA sequences, differential miRNA expression, transposon activations, and gene silencing or pseudogenization in nascent allohexaploid wheats^5,11^. The successful speciation of an allopolyploid often provides the new species with better adaptation to a wide range of climates as the heterogeneity from subgenomes could provide genetic advantage (hybrid heterosis) compared to the parental species^4,11–13^.

In addition to enhanced adaptability, the addition of the D genome in allohexaploid wheat is largely responsible the unique viscoelastic properties required for breadmaking^14,15^. The wheat dough properties are primarily controlled by prolamin storage proteins synthesized and accumulated in endosperm tissue during seed development. Wheat prolamins can be classified into glutenin and gliadin groups. The glutenin group contains HMW-glutenin and LMW-glutenin subunits involved in the formation of gluten polymers through intermolecular disulfide bonds, providing the elasticity to wheat dough. Gliadins, which are monomers, are viscous and give the dough its extensibility. These gliadins are subdivided into α, γ, δ and ω-gliadins based on electrophoretic mobility^16,17^.

Like prolamins in other cereal crops, wheat prolamin genes belong to large multigene families. Understanding their structure, evolution, and function in relation to baking quality is challenging, but essential for improving the end-use quality of wheat flour^16^. In wheat, the prolamins are primarily mapped to three genomic regions. HMW-glutenin genes are located at the *Glu-1* loci on the long arms of the wheat group 1 homeologous chromosomes^18^, while the short arms of the same chromosomes carry the *Glu-3* loci encoding the LMW-glutenins and the *Gli-1* loci encoding γ-, δ-, and ω-gliadins^19,20^. Studies have shown that the *Glu-3* and *Gli-1* loci are tightly linked based on genetic and genomic sequence analyses^19,21^. The third genomic region located on the short arms of wheat group 6 chromosomes harbors the *Gli-2* loci encoding α-gliadins. Gene family members arrayed in clusters are often prone to genetic variations in copy number, sequence polymorphism, and gene expression. Allelic variations in the composition of prolamins have been shown to be strongly correlated with differences in the breadmaking quality in different wheat varieties^22,23^. Both the quality (coding sequence variation) and quantity (differential gene expression) of prolamins contribute to dough properties of flour from different cultivars^16^.

Of the three major genomic regions harboring wheat prolamin genes, the *Gli-2* loci encoding α-gliadins are the youngest by evolution, as closely related species in Triticeae tribe such as barley and rye do not carry α-gliadin genes^24^. The α-gliadin gene family is also the most complex with estimates of copy numbers ranging from 25 to 150 in different wheat cultivars and ancestral species^25,26^. In addition, α-gliadins are important as they account for 15–30% of the total seed storage proteins in the wheat grain^27^. Unfortunately, α-gliadins are also major triggers of celiac disease (CD), a food-sensitive autoimmune disorder that impacts about 0.7–2% of the human population worldwide^28^. The most significant T-cell epitopes in celiac patients are PFPQPQLPY (DQ2.5-glia-α1a), PYPQPQLPY (DQ2.5-glia-α1b), PQPQLPYPQ (DQ2.5-glia-α2) and FRPQQPYPQ (DQ2.5-glia-α3)^28^. In some α-gliadins, one DQ2.5-glia-α1a, two DQ2.5-glia-α1b and three DQ2.5-glia-α2 epitopes overlap in a 33-mer peptide that is resistant to proteolytic digestion and is highly toxic in celiac patients^28^. A breeding effort to develop wheat with reduced levels of immunologically active epitopes, while retaining baking functionality, has been proposed^29^. A comprehensive understanding of α-gliadin genomic regions through detailed sequence analyses could facilitate the breeding effort by unraveling their genetic diversity and developing molecular markers for selections in breeding programs.

Due to the genomic complexity of the *Gli-2* locus regions, the structure and evolution of the α-gliadin gene family in the hexaploid wheat genome has not been well understood. Although several hundreds of α-gliadin gene sequences from polyploid wheats and their ancestral diploid species are available in NCBI GenBank, they were primarily obtained by sequencing cDNA or PCR amplified clones, transcriptome RNA-seq analysis, or low-coverage shotgun genome sequencing^30–33^. However, the origin and evolutionary relationships of α-gliadin family members are often difficult to draw in the absence of the context of their genomic organization. Recently, five α-gliadin-containing BACs from hexaploid wheat were sequenced to reveal sequence compositions surrounding the α-gliadin genes^34^. Since the sequences of these BACs were not contiguous and covered portions of the α-gliadin locus genomic regions, comparisons among the homeologous α-gliadin regions to reveal sequence changes were limited. A high-quality sequence that covers the entire *Gli-2* locus region has only been reported for *Ae. tauschii*, the progenitor of the wheat D genome^24^. Comparative analysis of orthologous genes from other grass genomes revealed that rapid and dynamic evolution only occurred in the *Ae. tauschii Gli-2* region^24^. In this study, we generated contiguous sequences of α-gliadin genomic regions for the A, B, and D genomes of the hexaploid wheat, cv Chinese Spring using genome sequence contigs constructed from PacBio long reads^35^. The sequence assemblies were improved and validated by aligning them to the Chinese Spring BioNano genome maps. The high-quality sequences generated here allow a detailed comparison of the three homeologous α-gliadin regions from the wheat A, B, and D subgenomes and provide insights into the structure and evolution of this important gene family.

## Results

### Construction of hexaploid wheat optical genome map and sequence assembly of α-gliadin regions

Sequences of complex genomic regions like the wheat prolamin gene loci containing tandem gene copies are often difficult to resolve using short sequence reads, particularly in large and highly repetitive genomes. PacBio single molecule real-time (SMRT) technology, which generates long sequence reads (average 10 kb), has proven effective in sequencing such complex genomic regions^36^. The Chinese Spring (CS) hexaploid wheat genome has been sequenced and assembled using deep sequencing coverage from a combination of short illumina reads and long PacBio reads^35^. The final assembly contained 15 Gb sequence with a weighted average (N50) contig size of 232 kb. However, these large contigs were not anchored to chromosomes to build pseudomolecules. To reconstruct the sequences of the wheat α-gliadin locus regions, genes annotated from the *Ae. tauschii* α-gliadin region^24^ were used in a BLAST search to identify CS PacBio contigs. Since the CS genome was assembled using either Illumina-PacBio hybrid reads or PacBio only reads^35^, we used BLAST searches against both assemblies to identify overlapping contigs. A total of 38 contigs were identified (Table S1). Based on the initial overlapping sequence assembly and sequence mapping to the wheat chromosome-by-chromosome sequence data, 8, 18, and 12 contigs belong to the A, B, and D genomes, respectively. Contig sizes range from 12 kb to 758 kb (Table S1).

A high-resolution genome map is highly useful in validating and improving genome sequence assembly^36,37^. Here, we constructed a genome-wide enzyme restriction site-based optical BioNano (BNG) map for the hexaploid wheat cv Chinese Spring. A total of 2,947 Gb of data (>20 kb) was obtained from 196 runs (2,456 unique scans) in which 6,796,161 raw molecules (>180 kb) corresponding to 1,927 Gb, representing 113 × genome equivalents (~17 Gb), were used to *de novo* assemble a BNG map. These raw molecules were assembled into 11,727 BNG contigs, with N50 of 1.69 Mb and maximum BNG contig length of 10.52 Mb. The total length of the BNG map assembly is 14.2 Gb, covering 84% of the genome.

**Figure 1:**
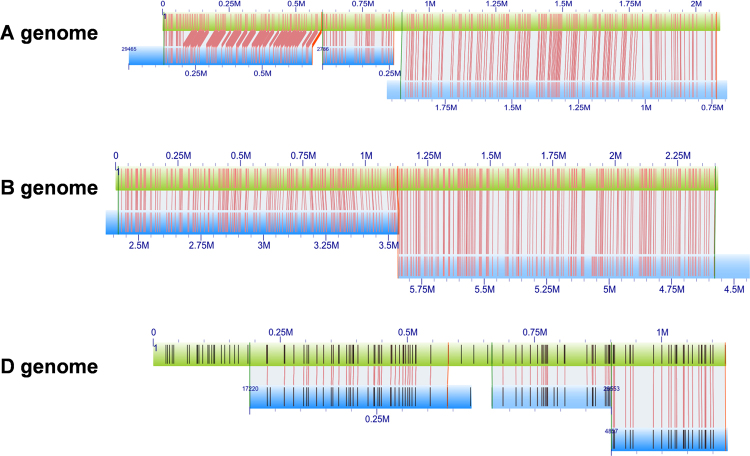
Alignment of sequence assemblies of α-gliadin genomic regions with BioNano genome map contigs. PacBio contigs of hexaploid wheat cv Chinese spring were extracted using genes annotated from the *Ae. tauschii* α-gliadin regions^24^, digested *in silico* with the restriction endonuclease, Nt.BspQ1, and aligned with the Chinese Spring BioNano genome map (blue bars). After manual editing and validation of the alignments, consensus sequences for α-gliadin regions were generated for the A,B and D genomes (green bars). Vertical lines represent agreements of sequence motifs of the endonuclease recognition site (GCTTCTTC) between the consensus sequences and BioNano map contigs.

To validate the sequence assembly and reconstruct the genomic regions spanning the entire *Gli-2* loci, *in silico* Nt.BspQ1 restriction maps of the PacBio contigs were generated and used to search against the CS BNG genome map data. Eight BNG contigs were identified for reconstructing the α-gliadin genomic regions by aligning the maps from BNG data with the sequence contigs, ordering and orientating contigs, resolving inconsistencies, and improving the final assembly (Fig. 1)^37^. We noticed that inconsistencies occasionally occurred at the end of BNG contigs, likely due to the chimeric issues of BNG contigs or inaccuracy in determining the restriction sites at the ends^36^. The final consensus sequences are 2,047,019 (NCBI accession number, MF434820), 2,412,936 (MF434819), and 1,107,773 bps (MF434818) for the A, B and D genome, respectively.

### Synteny comparison of α-gliadin regions from the homeologous wheat genomes

**Figure 2:**
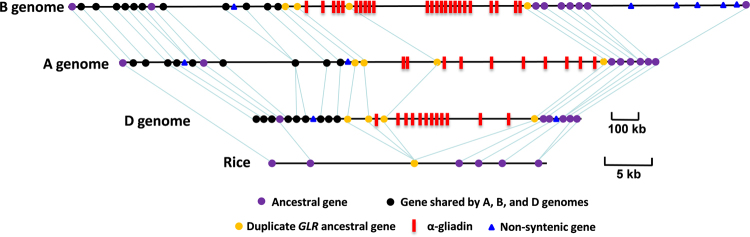
Synteny comparison of three wheat homeologous α-gliadin regions and rice orthologous region. Seven ancestral genes that are shared in grass genome^24^ are indicated as purple dots and connected by lines except the gene encoding glutamate-like receptor (yellow), which is single copy in rice, but duplicated to have four copies in the A,B and D genomes. Triticeae specific genes that are not shared with rice are shown in black dots. Non-syntentic genes that are not shared among the A,B and D genomes are represented by blue triangles. α-gliadin genes are indicated with red bars. Note that the size scale bar for wheat and rice is different.

Previously, we annotated 39 genes in the 1.7-Mb *Ae. tauschii* α-gliadin region^24^. These genes, particularly those located at the ends, were used to define the homeologous regions of the wheat A, B, and D subgenomes. In the wheat homeologous regions, we annotated 33, 54, and 33 genes in the A, B, and D genome, respectively (Fig. 2 and Table S2). When the gene content in the wheat homeologous A, B, and D regions were compared, all the seven ancestral genes identified in the previously investigated grass genomes were also conserved (Fig. 2)^24^. As in the *Ae. tauschii* genome, duplication of ancestral genes were observed in the wheat genomes, resulting in an increase of the total gene number. For example, a single copy ancestral gene encoding a glutamate-like receptor (*GLR*) in rice, *Brachypodium*, and sorghum was duplicated to have four copies in all wheat genomes (Fig. 2), suggesting that these duplications occurred before separation of the A, B, and D genomes. When the three wheat genomes were compared, gene colinearity was generally well maintained. Most non-syntenic or Triticeae-specific genes identified when compared with *Brachypodium*, rice, and sorghum were shared among the wheat genomes, indicating that their insertions into this region occurred in the Triticeae lineage. Several genes unique to one wheat genome were also observed (Fig. 2). There was one in the A, two in the D, and six in the B genome. These genes were likely inserted after the divergence of the A, B, and D genome; therefore, they are lineage-specific genes that can be used to develop markers to distinguish the α-gliadin homeologous loci. In most cases, non-syntenic genes are duplicated and then translocated to different locations at a single gene level^38^. In the B genome, there were five consecutive non-syntenic genes in a 345-kb region (Fig. 2), suggesting a large translocation event.

### Genomic organization of α-gliadin loci in wheat genomes

We observed that α-gliadin genes are highly duplicated in each genome, with 10, 24, and 11 copies in the A, B, and D genome, respectively. All the α-gliadin genes in the three genomes were located between *GRL2* and *GRL4*, suggesting that α-gliadin gene duplication occurred after the *GRL* duplication (Table S2). The α-gliadin genes appeared to have a similar clustering pattern, with no genes intermixing with these prolamin genes except *GRL3*. The number of α-gliadin gene between *GLR2* and *GLR3* varied; one copy in the D genome, two in the A, and 5 in the B genomes. We also noticed that the distance between two α-gliadin genes are quite different. Most genes in the B genome cluster close to each other (~10 kb apart), while the genes in the A genome are more dispersed (~71 kb apart) (see discussion below).

To better understand the evolution of these prolamin gene clusters, we performed an in-depth sequence analysis to depict the genomic organizations of the α-gliadin-containing regions (Figs S1, S2, and S3). In the 696-kb region containing the α-gliadin genes from the A genome, the sequences between two α-gliadin genes are mostly composed of repetitive DNA elements. In most cases, a base structure consisting of a DNA transposable element (TE) named *Manor* and two long terminal repeat (LTR) TEs named *Ada* and *Sabrina* are present (Fig. S1), suggesting the duplication of these genes occurred after the formation of this base structure between two α-gliadin genes. The major differences between two α-gliadin regions (or intergenic spaces) are caused by the differential insertion of repetitive DNA elements, indicating that these insertion events occurred after the sequence duplications (Fig. S1). The insertion of TEs greatly expanded the intergenic spaces in the A genome, with an average size of ~71 kb in the region from *α-A3* to *α-A10* (Fig. S1). The intergenic region between *α-A1* and *α-A2* is the smallest, about 37 kb, and contains the fewest TEs. The largest gap is between *α-A2* and *α-A3*, where the presence of the *GLR3* gene and massive insertions of TEs resulted in a 147-kb separation.

In the 387-kb α-gliadin region from the D genome, the base sequence structure between genes contained a *Hawi* LTR element that had been expanded by an insertion of 27-kb sequences flanked by an 8-bp direct repeat (Fig. S2). This base structure was the same as the one first identified in the *Ae. tauschii* α-gliadin region^24^, indicating that the two D genomes from hexaploid wheat and *Ae. tauschii* share the same base structure. When the α-gliadin regions from *Ae. tauschii* and wheat D genomes were compared, it was found that they shared an average of over 98% sequence identity in the alignment regions. The major differences between these two genomes were in regions where differential TE insertions occurred. For example, the sequence in the regions from *α-D10* and *α-D12* mainly consists of TEs with nested insertion structure. We identified four additional TE insertions that occurred in the region between *α-D10* and *α-D*11 only in the Chinese Spring D genome. The *Ae tauschii* D genome had two additional CACTA elements inserted into a *Sabrina* TE between *α-D*^*t*^*11* and *α-D*^*t*^12^24^. Clearly, these differential TE amplifications represent sequence changes that occurred after the divergence of the two D genomes. Another important difference observed is that *Ae. tauschii* has one more α-gliadin genes than Chinese Spring. Based on sequence organization, we identified that *α-D*^*t*^3 in *Ae. tauschii* is absent in Chinese Spring. Interestingly, this gene is the most highly expressed α-gliadin gene in *Ae. tauschii*^24^. At this point, it is unclear if this was a deletion event in Chinese Spring or new gene duplication in *Ae. tauschii*. When we named the α-gliadin genes for the Chinese Spring D genome, we omitted the use of *α-D3* to describe its absence when compared to the *Ae. tauschii* region.

The 765-kb α-gliadin region from the B genome contains 24 prolamin genes, more than double the number from the A or D genome (Fig. S3 and Table S2). Most regions between two α-gliadin genes in the B genome do not have any TE insertion. The base structure with no TE insertion is around 10 kb in size (Fig. S3). In a few cases where TEs were present between two α-gliadin genes, it was found that the insertions of these TEs were not shared in the intergenic regions, suggesting insertions occurred after the duplications (Fig. S3). Only in two regions (between *α-B1* and *α-B2* and between *α-B2* and *α-B3*), there were shared TE insertions, suggesting that their duplication occurred after the formation of the nested structure. Analysis on repetitive DNA sequences also revealed that three α-gliadin genes in the B genome were disrupted by TE insertions (Fig. S3). In the case of α*-B19* and α*-B20*, two TE insertions first occurred to one of the copies, followed by duplication. In the case of *α-B22*, a nested structure involving 6 TEs was identified in the coding region, and as a result, the start and stop codons are now separated by ~100 kb apart. We also identified 14 TE insertions in the region between *α-B10* and *α-B11*, resulting in its expansion to ~245 kb. In this case, the first insertion of TE at the bottom layer could not be identified, although the insertion site can be clearly identified by comparing with other intergenic regions (Fig. S3). One possible explanation is that this large TE nested insertion region might have been translocated from another location in the genome.

When the homeologous regions were compared, it becomes clear that genomic organizations of α-gliadin regions are quite different in the A, B and D genomes. In particular, the sequence structures of intergenic regions between the two genes have greatly diverged. Considering that most duplications of α-gliadin genes extended into the intergenic regions, we can speculate that the duplication of α-gliadin genes in the different genomes are independent or they occurred after the different genomes separated from their common ancestral species.

We also assessed if any α-gliadin genes might be present in other chromosome regions due to translocation. Only one contig, ctg006455F, was identified. This region contained two α-gliadin genes separated by a 3 kb sequence and was mapped to chromosome 6B by BLASTn search against the CS individual chromosome sequence database. Therefore, they were named *α-B25* and *α-B26*, respectively (Fig. S2).

### Characterization of α-gliadin genes and the encoded proteins

A total of 47 α-gliadin genes were identified in the CS genome (Table S2 and Fig. S4). Among them, 26 encode full-length proteins – eight from the A genome, 11 from the B genome, and seven from the D genome (Table 1). These genes encode proteins with predicted MWs that range from 30 to 36 kDa and pIs from 6.2 to 8.3. All consist of a signal peptide, an N-terminal repetitive domain with abundant glutamine and proline residues and two polyglutamine domains (polyQ I and polyQ II) that are interspersed with two non-repetitive domains. The number of glutamines in the polyQ I domain ranges from 11 to 36 amino acids with averages of 20 for the A genome, 18 for the B genome and 15 for the D genome (Table 1). In comparison, the polyQ II regions of proteins encoded by the B genome are notably larger than those of proteins from the A or D genomes. The average number of glutamines in polyQ II is 23 for B genome α-gliadins, and 9 and 11 for the A and D genome proteins, respectively.

**Table 1:** Characteristics of proteins encoded by complete α-gliadin genes from Chinese Spring.

	Predicted MW	Predicted pI	# Q in polyQ I	# Q in polyQ II	# CD Epitopes^1^						CSTT motif^2^
					DQ2.5- glia-α1a	DQ2.5- glia-α1b	DQ2.5- glia-α2	DQ2.5- glia-α3	DQ8/DQ8.5-glia-α1	33-mer	
α-A1	31440	6.37	14	9					1		x
α-A2	34470	6.5	29	14					1		x
α-A4	30506	7.12	14	7	1			1			
α-A5	33479	6.62	36	8	1			1			
α-A6	30621	7.79	16	6	1			1			
α-A8	31050	7.08	17	7	1			1			
α-A9	32181	6.53	23	12	1						
α-A10	29996	6.18	11	7	1			1			
α-B3	36206	7.76	22	45					1		x
α-B7	33968	7.12	18	23							x
α-B8	34781	8.28	23	25							x
α-B9	33977	7.16	18	22							x
α-B11	31535	7.07	17	16							x
α-B14	31413	7.02	17	16							x
α-B15	32054	6.42	15	23							x
α-B16	32039	6.42	19	18							x
α-B17	31714	7.12	19	16							x
α-B18	31828	7.12	15	13							x
α-B25	33817	7.78	12	36					1		x
α-D1	30699	6.53	13	12					1		x
α-D4	31542	6.79	16	10	1		1	1			
α-D5	33412	7.75	20	12	1	2	3	1	1	1	
α-D6	31706	6.53	13	14	1	1	2		1		
α-D8	31706	6.53	14	11	1	1	2	1	1		
α-D12	30175	6.44	11	6							x

^1^CD-relevant epitopes include DQ2.5-glia-α1a (PFPQPQLPY), DQ2.5-glia-α1b (PYPQPQLPY), DQ2.5-glia-α2 (PQPQLPYPQ), DQ2.5-glia-α3 (FRPQQPYPQ), DQ8-glia-α1/DQ8.5-glia-α1 (QGSFQPSQQ) and 33-mer toxicpeptide (LQLQPFPQPQLPYPQPQLPYPQPQLPYPQPQPF) (Sollid *et al*.^28^).

^2^Motif reported by Wang *et al*.^30^. x indicates that motif is present in sequence.

α-gliadin genes have a high rate of pseudogenization. Twenty one (45%) out of 47 α-gliadin genes were identified to be pseudogenes - two genes (20%) from the A genome, 15 (57%) from the B genome, and four (36%) from the D genome. Among the 21 pseudogenes, thirteen (59%) were caused by nucleotide substitutions, generating one or multiple premature stop codons in the coding region (Fig. S4). In most cases, stop codons were the result of C to T changes, altering CAG or CAA codons for gluatamine residue into TAG or TAA stop codons. Three pseudogenes from the B genome (*α-B4*, *α-B5* and *α-B24*) and one from the D genome (*α-D*2) contain only partial coding regions likely due to deletion events. We also found that pseudogenes could contain multiple sequence rearrangement events that can all result in pseudogenization. For example, *α-B19*, *α-B20* and *α-B22* all contained TE insertions, premature stop codons, and frame shift indels (Figs S2 and S4). In these cases, the initial mechanisms underlying pseudogenization could not be determined.

We also compared the α-gliadin pseudogenes of the D genomes from Chinese Spring and *Ae. tauschii* to assess if the mutation events in hexaploid wheat were inherited from an ancestral genome. It appears that three pseudogenes (*α-D2*and *α-D*^*t*^*2*, *α-D*10 and *α-D*^*t*^*10*, and *α-D*11and *a-D*^*t*^*11*) were shared. *α-D*2 and *α-D*^*t*^*2* were both gene fragments. *α-D10* and *α-D11* from CS all contained multiple premature stop codons and at least one or two premature stop codon mutations were shared by the homologous copy in *Ae. tauschii*. These shared mutations are likely inherited from the ancestral genome. We also identified two independent mutation events in each genome: *α-D*^*t*^4 and *α-D*^*t*^12 from *Ae. tauschii* and *α-D7* and *α-D9* from hexaploid D. These pseudogene sequences were all verified by RNA-seq data to ensure that they were not caused by sequencing errors (see discussion below).

### Phylogeny of α-gliadin genes from the homeologous wheat genomes

**Figure 3:**
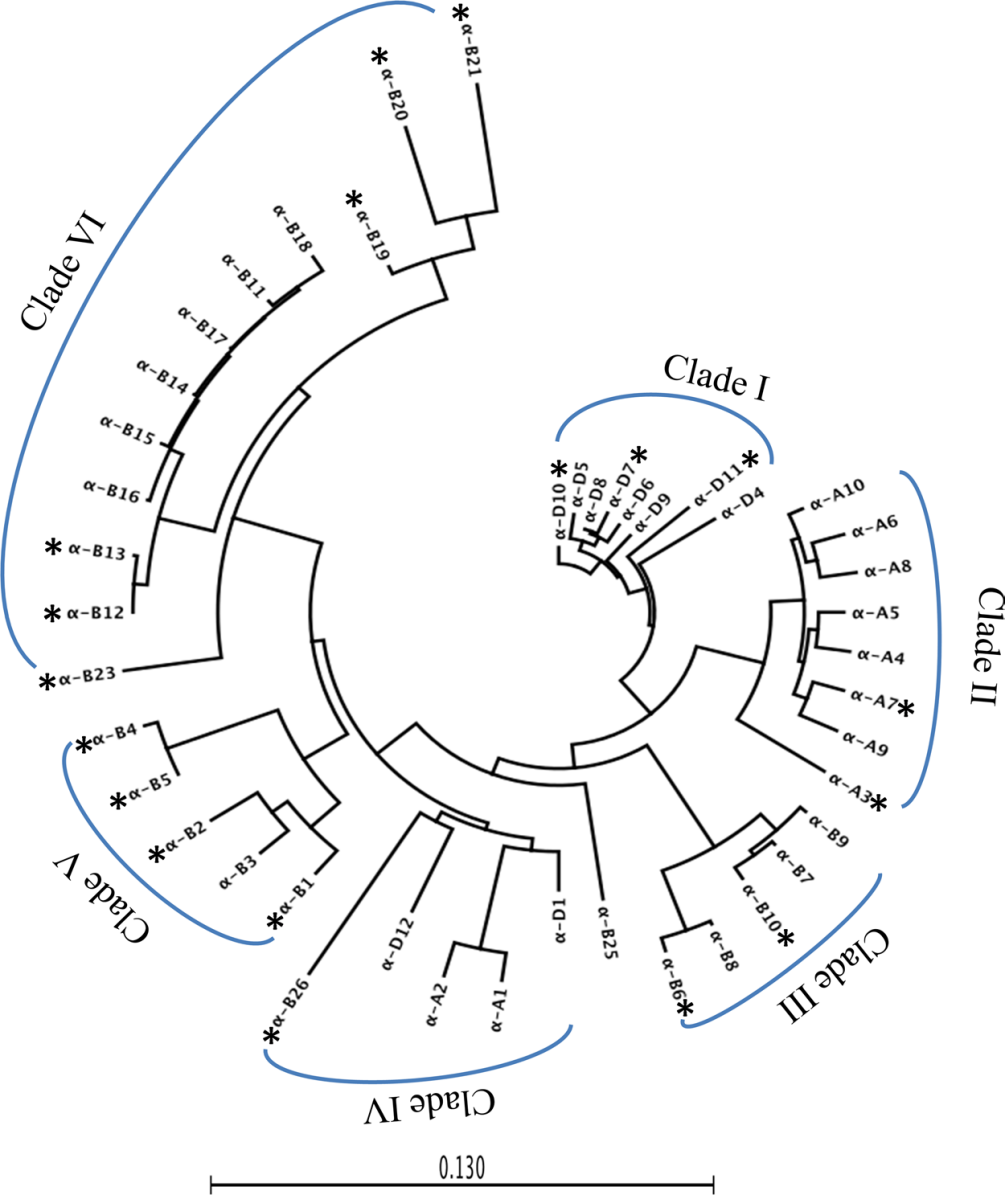
Phylogenetic analysis of α-gliadin genes from hexaploid wheat cv Chinese Spring. Nucleotide sequences of α-gliadin genes and pseudogenes were aligned and a phylogenetic tree was reconstructed using MEGA6 with the UPGMA methods. The tree was drawn to scale, with branch lengths measured in the number of substitution per site. TE sequences were removed from pseudogenes containing TE insertions before analysis. Pseudogenes are indicated by *.

To further infer the evolutionary relationship of α-gliadin genes, a phylogenetic tree was constructed using the α-gliadin genes identified from the A, B, and D genomes (Fig. 3). Six major clades were observed (I to VI), with α-gliadin genes from the same genome generally grouped together. Clade I has only eight α-gliadin genes from the D genome, clade II contains eight α-gliadin genes exclusively from the A genome, and B genome formed three separate clades (Fig. 3). The α-gliadin genes located between two *GLR* genes (*GLR2* and *GLR*3) (Fig. 2) made up Clade V, suggesting duplication of these genes occurred within this defined region. Clade III consisted of α-gliadin genes from *α-B6 to α-B10*. The largest clade, Clade VI, was composed of 12 α-gliadin genes from the B genome (Fig. 3). Only Clade IV contained α-gliadin genes from all three genomes, suggesting that these genes represent ancestral forms of the α-gliadin genes that existed before the separation of A, B, and D genomes. In this clade, *α-D1* and *α-D*12 are the first and last α-gliadin genes in the *Gli-2* locus region from the D genome. *α-B26* and *α-D*1*2* were subgrouped together in Clade IV, supporting the notion that the last α-gliadin gene in the B genome *Gli-2* locus was moved to its current position through a translocation event. In addition, we found that the first α-gliadin gene from the B genome and the last α-gliadin gene from the A genome were not grouped in this clade. It is possible that the corresponding genes have been deleted during evolution.

### Analysis of α-gliadin gene expressions during grain development

**Figure 4:**
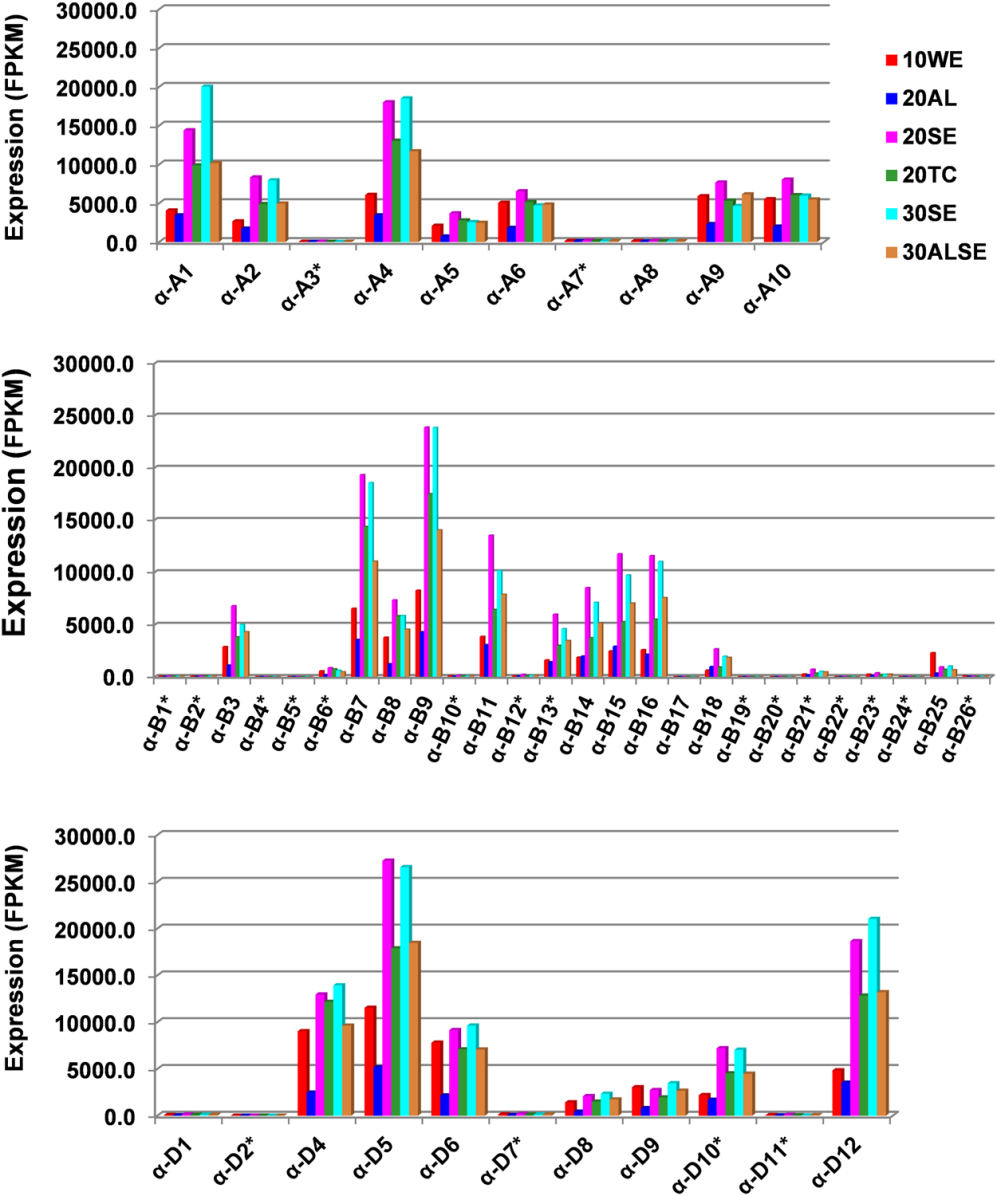
Expression of α-gliadin genes at different grain development stages and tissues. Transcriptome data generated from different grain development stages and tissues were downloaded from published results^55^. 10WE represents whole embryo at 10 days post-anthesis, 20AL, aleurone layer at 20 days, 20SE, seed endosperm at 20 days, 20TC, transfer cell at 20 days, 30SE, seed endosperm at 30 days, and 30ALSE, aleurone plus endosperm at 30 days. Using CLC genomic workbench RNA-seq analysis toolbox, the RNA-seq reads were mapped to the annotated wheat gene set^50^ except that the α-gliadin genes were replaced with the α-gliadin genes identified in this study. FPKM values were calculated using the functions in the toolbox. Pseudogenes are labeled with an * symbol.

The expression of α-gliadin genes was examined using available transcriptome data generated from different seed tissues at different seed developmental stages^9^. It can be challenging to estimate gene expression through sequence assembly of short reads in wheat prolamin genes with highly repetitive domains^30^. Here, we assessed gene expression by mapping Illumina reads to the annotated gene set. Mapping reads to well annotated α-gliadin genes provides a more accurate view of gene expressions as compared to the *de novo* assembly methods^24^. During this process, we also checked mapped Illumina reads to validate individual α-gliadin gene sequences derived from the PacBio assembly. In particular, all the mutations in the pseudogenes were confirmed. The RNA-seq transcriptome analysis result indicated that prolamin genes are highly expressed. Based on the reads mapped to the α-gliadin genes against the total reads mapped to the complete annotated gene sets in wheat, we estimated that α-gliadin transcripts account for 23% of the total mapped transcript reads in the seed endosperm (SE) at 20 day post-anthesis (Table S3). Even when total RNA-seq reads, instead of total reads mapped to the complete gene set, were used in the calculation, the α-gliadin transcripts accounted for 13.9%. Among the prolamin genes, the level of expression varies greatly. There are six genes with FPKM values above 15000 at the peak expression, while six other genes had FPKM values under 5000. As expected, the transcript levels of prolamin pseudogenes are very low compared to most intact prolamin genes. The low expression of pseudogenes is likely correlated with the instability of their transcripts during translation^39^. However, we found two pseudogenes, *α-D10* and *α-B1*3, with FPKM values over 5000 - higher than the FPKM values of some intact prolamin genes (Fig. 4). It is unclear why these two genes have relatively high expression levels. However, it is possible that mRNAs from different pseudogenes might degrade at different rates. We also compared the expression levels of α-gliadin genes from the A, B, and D genomes to assess differences among the homeologous loci. Although the B genome had the largest number of prolamin genes, it also had a high percentage of pseudogenes (54%). The B genome has 9 expressed genes with FPKM values above 5000 as compared to 6 in the D and 7 in the A genomes. This supports the idea that the B genome contributes a large portion of α-gliadins to seed protein composition in Chinese Spring.

We also examined the expression of individual α-gliadin genes in different tissues at different developmental stages (Fig. 4). Some α-gliadin genes had strong upregulation in expression from 10 to 30 days post-anthesis, while others showed similar expression levels at different developmental stages (Fig. 4), indicating differential responses of gliadin genes during seed maturation. At 20 days post-anthesis, transcriptome data was available from the aleurone layer (AL), starchy endosperm (SE), and transfer cell (TC). Although individual α-gliadin genes showed different patterns of expression, the highest overall expression of α-gliadin was in starchy endosperm, where starch granules and storage proteins accumulate during seed development. The lowest expression was in the aleurone layer, which primarily accumulates lipid bodies. Transfer cells, which transport sucrose from photosynthetic tissues to the endosperm and embryo, also had significant expression of α-gliadin genes. It is not clear if the FPKM values reflect true expression levels in TC, or if they may be the result from contamination with starchy endosperm during the process of manual tissue dissection.

We also compared the expression of the homologous α-gliadin pairs from the D genomes from Chinese Spring and *Ae. tauschii*^24^. It was found that their expression can be quite different. As previously described, the corresponding gene of *α-D*^*t*^*3*, representing the highest expression in *Ae. tauschii*^24^, was not present in the hexaploid D genome. The second highest expressed gene *α-D12* in Chinese Spring was a pseudogene (*α-D*^*t*^*12*) in *Ae. tauschii*. The homologs *α-D*^*t*^*1* and *α-D1* are both intact genes. In Chinese Spring, it is highly expressed, while in *Ae. tauschii*, it has very low expression levels for an intact gene. This data suggests that the expression patterns of individual α-gliadin genes have changed dramatically since the divergence of the two D genomes.

### Analysis of α-gliadin CD epitopes

**Figure 5:**
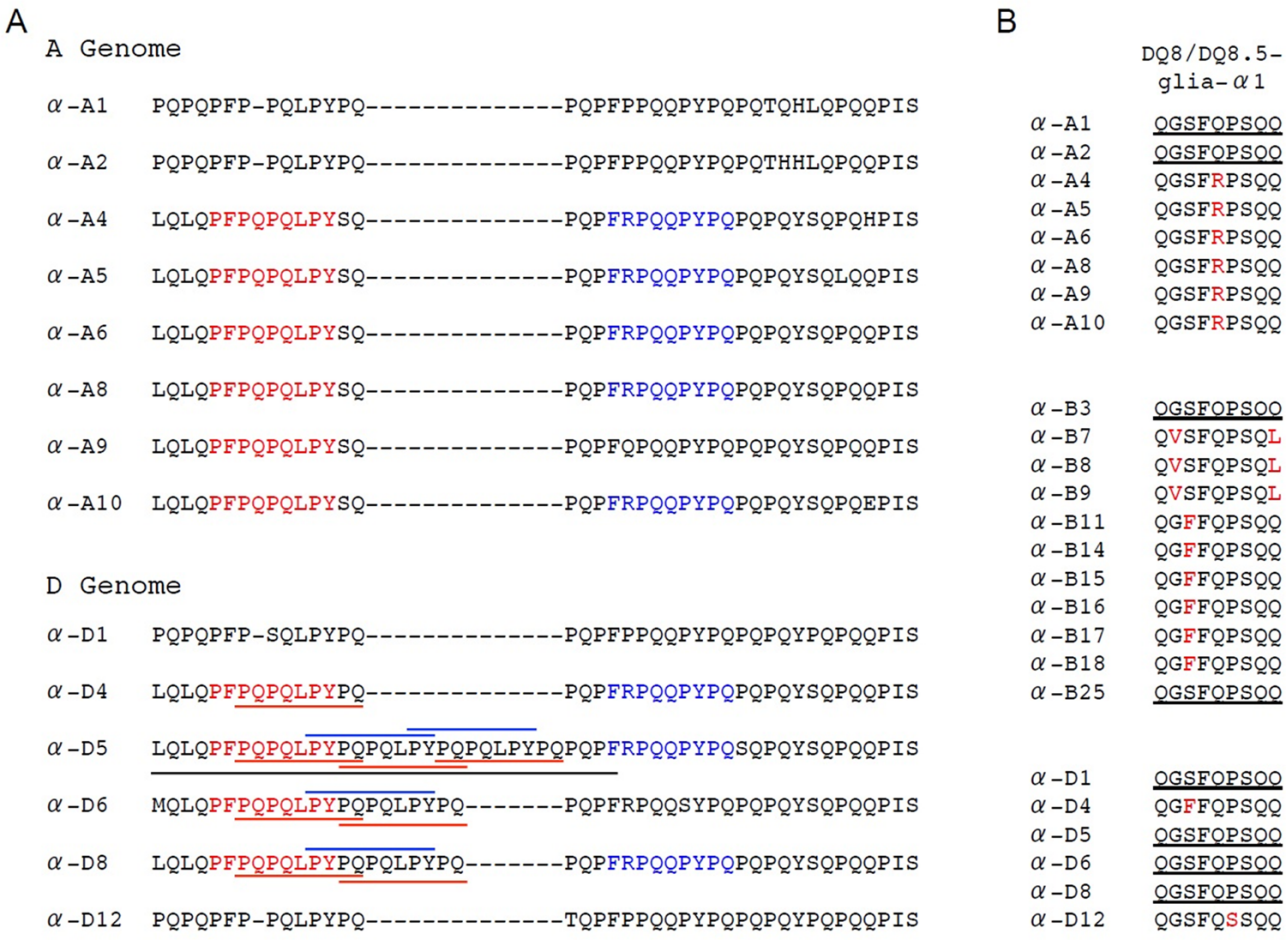
CD epitope analysis of α-gliadins in hexaploid wheat cv Chinese Spring. (**A**) Major CD epitopes in the repetitive portion of α-gliadins encoded by the A and D genomes. DQ2.5-glia-α1a and DQ2.5-glia-α3 epitopes are shown in red and blue, respectively. DQ2.5-glia-α1b epitopes are indicated by the blue lines above sequences, DQ2.5-glia-α2 epitopes are underlined in red and the 33-mer toxic peptide is underlined in black. Epitopes shown or underlined in red were found to be immunodominant by Tye-Din *et al*.^40^ α-gliadins encoded by the B genome contain none of these epitopes. (**B**) Sequence variation in regions of the DQ8/DQ8.5-glia-α1 epitope in α-gliadins encoded by the A,B and D genomes. Epitope sequence is underlined. Amino acids that differ from the canonical epitope are shown in red.

α-Gliadins are the major triggers of CD disease. Analysis of translated protein sequences indicated that fifteen of the α-gliadins (60%) contain epitopes capable of stimulating T-cells from CD patients (Table 1)^28^. Proteins encoded by the D genome contain the greatest number of CD epitopes. All of the major CD epitopes are represented in this group of proteins (Table 1, Fig. 5A,B). Four proteins contain three to eight CD epitopes (α-D4, α-D5, α-D6, α-D8) including the DQ2.5-glia-α1a and DQ2.5-glia-α2 epitopes that have been found to be immunodominant^40^. α-D5 also contains the 33-mer toxic peptide that is particularly immunogenic and has been shown to be resistant to proteolytic digestion (Shan *et al*., 2002). In comparison, the protein encoded by α-D1 contains only the DQ8/DQ8.5-glia-α1 epitope while the protein encoded by α-D12 does not contain any CD epitopes. All proteins encoded by the A genome contain either one (α-A1, α-A2, α-A9) or two (α-A4, α-A5, α-A6, α-A8, α-A10) CD epitopes. Six contain immunodominant DQ2.5-glia-α1a epitopes (shown in red in Fig. 5A), five have DQ2.5-glia-α3 epitopes (shown in blue in Fig. 5A) and two have DQ8/DQ8.5-glia-α1 epitopes (Fig. 5B). Proteins encoded by the B genome are the least immunogenic. Of the ten proteins encoded by the B genome, only two contain CD epitopes. α-B3 and α-B25 contain single copies of the DQ8/DQ8.5-glia-α1 epitope (Table A1, Fig. 5B). Of the 25 α-gliadins, two from the A genome, 11 from the B genome and two from the D genome contain CSTT near the C-terminus of the protein, a motif first noted by Wang *et al*.^30^ (Table 1). Consistent with their findings, the CSST motif is found solely in proteins without CD epitopes or those that contain only epitopes that bind the DQ8 and DQ8.5 human leukocyte antigen proteins (α-A1, α-A2, α-D1).

One interesting finding is that the α-gliadin 33-mer toxic peptide is only present in one α-gliadin gene (α-D5) from the wheat D genome, while the homologous copy (α-D^t^5) from the progenitor D genome of *Ae. tauschii* does not contain this peptide sequence (Fig. S5). Sequence alignment at both nucleotide and amino acid levels shows that there is a 21-nucleotide indel event that coincides with the position of the 33-mer, suggesting that a single indel event resulted in the formation of the specific 33-mer peptide (Fig. S5). Whether it is a deletion in *Ae. tauschii* or insertion in hexaploid wheat remains unclear. However, previous studies based on sequencing of multiple diploid and polyploid wheat lines indicated that the 33-mer epitope is only present in hexaploid wheat^31^. Therefore, we propose that the occurrence of the 33-mer epitope was caused by an insertion event that occurred in the D genome after the allohexaplodization. It is notable that α-D5 is the most highly expressed α-gliadin in Chinese Spring.

## Discussion

Wheat prolamins are an important nutrition source for humans worldwide and contribute unique functional properties that make it possible to produce a wide range of food products. Generating high-quality sequences in genomic regions harboring wheat prolamin loci for comparative analysis is challenging due to the polyploidy nature of the genome, high repetitive DNA content, and large and complex gene families. In this work, we used a BioNano genome map and the PacBio contigs to reconstruct three homeologous α-gliadin genomic regions of the A, B, and D genomes from the hexaploid wheat cv Chinese Spring. This work represents the first in-depth view of structural organization of a full complement of α-gliadin genes from the genetic background of a single hexaploid wheat species. Our detailed comparative analysis revealed dynamic evolution of the α-gliadin gene family in hexaploid wheat.

### Gene collinearity in homeologous *Gli-2* regions in hexaploid wheat

Comparative genomics studies have shown that Triticeae genome has a greater number of dispersed genes than other genomes such as rice, *Brachypodium*, and sorghum and is evolving an order of magnitude faster with regard to the structural rearrangement of its chromosomes^38,41,42^. It has been hypothesized that the exceptionally high amount of very similar TEs can result in frequent recombination errors and cause gene duplication and structural chromosome changes^43^. So far, most comparative analyses of Triticeae genomes were performed against small and compact grass genomes such as rice, *Brachypodium*, and sorghum that have diverged at least 25 million year ago. Large scale comparative analyses among Triticeae genomes could reveal recent evolution rates in Triticeae species, but only a few studies have been reported. Analyses of homeologous genomic regions of BAC insert sizes from the wheat A, B, and D genomes revealed that although gene collinearity is well maintained, the intergenic regions are completely divergent due to differential insertions and deletions of TEs^18,44^. Recently, the short arm sequences of *Ae. tauschii* 3D chromosome (At3DS) were compared with the homeologous regions from the Chinese Spring 3B chromosome (Ta3BS). In the syntenic regions, 2222 and 3101 genes were annotated for At3DS and Ta3BS, respectively. Collinearity analysis identified 1296 collinear gene pairs between the homeologous regions, representing 58.32% of genes in At3DS and 47.79% in Ta3BS. The low percentage of collinear genes between the two genomes is surprising, but supports the notion of the fast evolution of Triticeae genomes^38,42,43^. One potential problem with genome-wide syntenic analyses is that only annotated genes were used in the comparison. Gene annotation is complicated and can result in variations in gene numbers when different annotation pipelines are used because of difficulty in recognizing short protein-coding genes, distinguishing functional genes from non-functional pseudogenes, and assembling regions containing repeats and duplications^45^. For synteny comparisons, it is important to include both functional genes and pseudogenes to reveal genomic structure changes. In the homeologous α-gliadin regions, genes were annotated using a combination of the automated TriAnnot pipeline and manual annotation, since precise prediction of the full complement of wheat prolamin genes is difficult because of premature stop codons, frame shift mutations, microsatellites from repeat codons for glutamine, and gene disruptions by sequence deletions and TE insertions. Our syntenic analysis indicated that for the non-prolamin genes, in addition to the syntenic relation of ancestral genes shared among grass species, ten Triticeae-specific genes were conserved among the three homeologous genomes (Fig. 2). Non-syntenic genes for each genome were detected, but not as frequently as described in the comparison of At3DS and Ta3BS^38,42^. Therefore, in-depth comparative analyses will be needed to have a better understanding of genomic structural variations in the Triticeae genomes.

### Lineage-specific evolution of α-gliadin genes in A, B, and D genomes

Tandem gene duplication is one of the major gene duplication mechanisms in eukaryotes, as illustrated by the prevalence of gene family clusters. Tandem gene duplication arises through unequal crossing over, which often results from homologous recombination between paralogous sequences^46^. The expansion of α-gliadin genes clearly represents such an example of gene duplication. Unlike copy number increases *via* whole genome duplication, tandem gene duplication occurs more frequently and can result in rapid expansion of gene family numbers. α-gliadin genes are the youngest group of prolamins in wheat and its ancestral species^24^. Τhey are not present in some closely related species, such as rye and barley, which separated from wheat about 7 to 11 MYA^7^. Comparative analysis on the syntenic regions from several grass species indicated that the α-gliadin loci originated from translocation of a prolamin gene sequence located on the short arm of chromosome 1. This translocation event likely occurred in a lineage after the separation of wheat-related ancestral species from rye and barley^24^. The wheat A, B, and D homeologous chromosomes have an estimated divergence time of about 2.3–2.4 MYA^7^. Our analysis showed that despite the high collinearity of non-prolamin genes, most α-gliadin genes in one genome had no obvious orthologous counterparts in the other homeologous chromosomes. This observation was primarily based on genomic structural organization of α-gliadin genes in the A, B, and D genomes (Figs S1, S2, and S3). It was found that the base structure for α-gliadin gene duplication was different in size and TE insertions (Figs S1, S2, and S3), suggesting duplications in expanding the gene family members were not shared events among A, B, and D genomes. The phylogenetic tree shows that most α-gliadin genes from the same genome are grouped into the same clades, indicating that genes from the same chromosome are more closely related. Taken together, our results indicate that the duplication and expansion of α-gliadin in the three genomes occurred independently in the last ~2.4 million years.

The phylogenetic tree also identified one clade that contained α-gliadin genes from all three homeologous chromosomes (Fig. 3). These genes could represent ancestral gene forms. It appears that two copies of α-gliadin genes were present before the divergence of the A, B and D genomes. Rapid amplification of lineage-specific α-gliadin genes took place between the two ancestral copies. In the course of α-gliadin gene evolution in different genomes, sequence rearrangements have occurred to these ancestral genes. For instance, the orthologous counterpart of *α-D1* in the B genome and *α-D12* in the A genome have been deleted. The B genome counterpart (*α-B26*) of *α-D12* has been translocated to a different location. This implies dynamic changes of the ancestral copies after the formation of new prolamin copies. This observation is consistent with the studies on maize zein storage protein genes that suggested that after duplication, older copies of prolamin genes are often deleted or disrupted^47^. In maize, it was also found that once genes are duplicated, expression of the donor genes is reduced relative to new copies. Epigenetic regulation might contribute to the silencing of the older copies^48^.

### Expression regulation of tandem duplicated α-gliadin genes

One salient feature of the α-gliadin family is that the expression level of each member can be quite different (Fig. 4). One reason is pseudogenization, resulting in dramatic reduction of transcripts of α-gliadin pseudogenes that account for 46% of the total 47 genes identified in Chinese Spring. Many α-gliadin pseudogenes have promoter sequences with the same *cis*-regulatory elements as intact genes^24,34^. Therefore, the initiation of transcription might start normally. The low expression level likely results from regulation at the post-translational level by a mechanism called nonsense-mediated mRNA decay (NMD). The NMD mechanism involves degradation of premature termination codon-containing transcripts to prevent production of truncated proteins that could function in dominant-negative or other deleterious mechanisms^39^.

Even among the full-length intact genes, differences in expression were sometimes more than 50-fold (Fig. 4). Examination of α-gliadin promoter sequence regions does not provide an good explanation, as all the *cis*-regulatory elements controlling prolamin specific expression are highly conserved among the α-gliadin genes^24,34^. Likewise, our genomic sequence analysis of α-gliadin gene duplicates did not detect major sequence rearrangements in the promoter regions. Differential expression also could be the effect of subgenome dominance that causes higher expression of homeologous gene sets from the dominant subgenome in polyploid species^49^. However, in our transcriptome data analysis, differential expression was observed among the family members from each subgenome, and the levels of highly expressed genes in the three subgenomes were very similar (Fig. 5), suggesting that subgenome dominance doesn’t play significant role in regulating α-gliadin expression. Expression divergence is also a common phenomenon among the maize prolamin genes. Recently, it has been shown that epigenetic changes through DNA methylation in both promoter and coding regions contributed to expression divergence of prolamin genes^48^. The impact of epigenetics on wheat prolamin gene expression remains to be investigated. One interesting observation is that homologous α-gliadin gene pairs from the diploid *Ae. tauschii* and Chinese Spring D genome displayed significant difference in expression. Our analysis showed that except for the deletion of *α-D3* in CS and a few differential nested TE insertions, the genomic structural organization of the two homologous α-gliadin regions is highly conserved. We also noticed that while *α-D3* in CS is deleted, the homologous counterpart (*a-D*^*t*^*3*) has the highest expression in the *Ae. tauschii* genome^24^. Whether deletion of the highest expression gene could have resulted in change of DNA methylation status in the α-gliadin region is not clear. Finally, the difference in expression could be caused by reprogramming of gene regulation after the integration of the A, B, and D genomes into the same nuclei during allopolyploidization^3^.

In conclusion, our study represents a comprehensive analysis of three homeologous α-gliadin loci in a hexaploid wheat species. The rapid and dynamic duplication of α-gliadin genes, as well as the expression divergence among each copy and frequent mutations resulting in pseudogenes, could provide an explanation to the great genetic diversity in this important genomic region^32^. The identification of a full complement of α-gliadin genes from a single genetic background not only allows us to understand their evolutionary relationship but also provides a better view of CD epitope distribution and expression in wheat. For instance, we identified that a single indel event in the hexaploid wheat D genome resulted in generation of the 33-mer highly toxic CD epitope. The knowledge gained from this study will facilitate proteomic studies that provide insights into the roles of the α-gliadins in flour functionality and human health as well as the development of novel strategies for breeding elite wheat varieties with improved end-use traits and reduced immunogenic potential for human consumption.

## Materials and Methods

### *De novo* BioNano genome map assembly and analysis

High molecular weight (HMW) DNA was isolated from young leaves (grown in darkness) of hexaploid wheat (*Triticum aestivum L*.) genotype ‘Chinese Spring’ by Amplicon Express (Pullman, WA). The nicking endonuclease Nt.*Bsp*QI (New England BioLabs, Ipswich, MA) was chosen to label high-quality HMW DNA molecules at specific sequence motifs (GCTCTTC) based on sequences of the publicly available hexaploid wheat genome^50^. The nicked DNA molecules were then stained according to the instructions of IrysPrep Reagent Kit (BioNano Genomics, San Diego, CA). The stained DNA sample was loaded onto the nanochannel array of IrysChip (BioNano Genomics) and was automatically imaged by Irys system (BioNano Genomics). Raw DNA molecules >20 kb were collected and converted into BNX files by AutoDetect software to obtain basic labeling and DNA length information. The filtered raw DNA molecules in BNX format were aligned, clustered, and assembled into the BioNano genome (BNG) map by using the BioNano Genomics assembly pipeline as described in previous publications^51,52^. The *P* value thresholds used for pairwise assembly, extension/refinement, and merge stages were 1 × 10^−10^, 1 × 10^−11^, and 1 × 10^−15^, respectively. The initial BNG map was then checked for potential chimeric BNG contigs and was further refined.

### Sequence analysis and gene annotation

To identify the homeologous α-gliadin gene regions from hexaploid wheat cv Chinese Spring, genes previously annotated in the *Ae. tauschii* α-gliadin region^24^ were used for BLASTN search against the genomic sequence contigs of Chinese Spring generated using PacBio read-only assembly and hybrid assembly of BacBio and Illumina reads^35^. Sequence contigs with high stringent matches (*E* value less than 1e^−150^) were downloaded. To compare the sequences with the BNG map, the extracted sequences were digested *in silico* according to the restriction site of Nt.*Bsp*QI by using Knickers. The alignment of sequence assemblies with the BNG map was computed with RefAligner, and the visualization of the alignment was performed with snapshot in IrysView. Software and packages used can be obtained from BioNano Genomics (http://www.bionanogenomics.com/support/software-updates/). Manual check and editing are involved to improve the final assembly by aligning, merging, and reorienting contigs^37^.

For sequence annotation, the final assembled α-gliadin genomic sequences for the A, B, and D genomes were first submitted to TriAnnot pipeline for automated gene annotation^53^. The annotated genes were them compared with the gene contents from the *Ae. tauschii* α-gliadin regions^24^. In addition, a homology search was performed against the NCBI nonredundant databases using BLASTN, BLASTX, and TBLASTX algorithm to verify annotated genes and identify missed genes and pseudogenes. Because gene annotation often includes transposable elements, only genes that have homology in other monocots were included. DNA repetitive elements were annotated with DNAstar MegAlign dotplot analysis and by comparison with the TREP database (http://botserv2.uzh.ch/kelldata/trep-db/index.html).

### Transcriptome data analysis

A total of 176.5 Gbp Chinese Spring RNA-seq data derived from 3 time points (10, 20 and 30 days post-anthesis) and 3 main cell types/combinations (Seed endosperm, transfer cell and aleurone layer) were downloaded from NCBI (ERP004505). The Chinese Spring coding sequences (CDS) (TGAC v1.0) were downloaded from EnsemblPlants (ftp://ftp.ensemblgenomes.org/pub/plants/release-37/fasta/triticum_aestivum/cds/). The annotated α-gliadin gene sequences along with the TGAC CDS (minus the α-gliadin genes) were used as reference for RNA-Seq analysis using the CLC Genomic Workbench (v8.5) RNA-Seq Analysis Toolbox. Because of the high nucleotide similarities of α-gliadin genes, stringent mapping parameters with mismatch cost 2, insertion and deletion cost 3, length fraction 0.9, similarity 0.99 were employed in read mapping. The FPKM values were also calculated using the function in the CLC Toolbox. RNA-seq alignment was manually reviewed to confirm the assembly of α-gliadin gene sequences, including mutation sites causing pseudogenization.

### Phylogenetic tree analysis

Nucleotide sequences of α-gliadin gene coding regions were extracted and aligned using MUSCLE using default settings, with manual modification. Pseudogenes that were disrupted by TE insertions but contained the full-length gene sequences were included by removing the TE sequences. Phylogenetic analysis were constructed using the UPGMA method in the MEGA6 program^54^. The tree was draw to scale, with branch lengths measured by the bootstrap method with 1000 replications.

### Data availability

All the sequence data set and analysis results obtained in this work are available from the corresponding authors on reasonable request.

## Electronic supplementary material

Supplementary information
