# Supplementary material for: Dynamic Evolution of α-Gliadin Prolamin Gene Family in Homeologous Genomes of Hexaploid Wheat
Source: Sci Rep. 2018 Mar 26;8:5181. doi: 10.1038/s41598-018-23570-5 (PMC5980091; doi:10.1038/s41598-018-23570-5)
Supplement: Supplementary file 1 — Supplementary information [file 41598_2018_23570_MOESM1_ESM.pdf]

Supplementary Information

Dynamic Evolution of α-gliadin Prolamin Gene Family in Homeologous Genomes of Hexaploid Wheat

Naxin Huo[1,2*], Tingting Zhu[2*], Susan Altenbach[1], Lingli Dong[3], Yi Wang[1], Toni Mohr[1], Zhiyong Liu[3], Jan Dvorak[2], Ming-Cheng Luo[2], Yong Q. Gu[1]

[1]United States Department of Agriculture-Agricultural Research Service, Western Regional Research Center, Albany, California 94710, U.S.A.

[2]Department of Plant Sciences, University of California, Davis, CA 95616, U.S.A.

[3]State Key Laboratory of Plant Cell and Chromosome Engineering, Institute of Genetics and Developmental Biology, Chinese Academy of Sciences, Beijing 100101, China

*These authors contributed equally to the work

Corresponding authors:

Yong Q. Gu
USDA-ARS, Western Regional Research Center
800 Buchanan Street
Albany, CA 94710, U.S.A.
Phone: (510)559-5732 Fax: (510)559-5818
E-mail: yong.gu@ars.usda.gov

Ming-Cheng Luo
Department of Plant Sciences,
University of California, Davis
Davis CA 95616, U.S.A.
Phone: (530)754-6235
E-mail: mluo@ucdavis.edu

Table S1 Chinese Spring genome sequence contigs in α-gliadin regions

Genome	FALCON Trit 1.0	seqlength(bp)	Triticum1.0	seqlength(bp)
A Genome	000887F	758692	scf7180004654949	29177
	047975F	78715	scf7180004978732	200255
	001925F	632550		
	012734F	283772		
	069750F	25153		
	003013F	536353		
B Genome	021496F	205976	scf7180004963015	196592
	017729F	237923	scf7180004947566	315253
	050439F	60131	scf7180004993810	114424
	042920F	95938	scf7180004934336	18128
	001809F	651181	scf7180004866629	31491
	048447F	67769	scf7180004469127	45100
	005515F	434302		
	007786F	376564		
	068739F	26815		
	041981F	98458		
	001873F	608954		
	006455F	384913		
D Genome	009235F	335483	scf7180004912608	28315
	024844F	172037	scf7180004992456	105122
	078736F	12137	scf7180004818604	26410
	045628F	86322		
	067696F	28741		
	030968F	147003		
	010991F	311522		
	022739F	196559		
	003775F	492209		

Note: FALCON Trit 1.0- PacBio only contigs, Triticum1.0- PacBio and Illumina hybrid contigs

Table S2 Annotation of Chinese Spring *a* -gliadin gene regions and comparion with *Ae. tauschii*

	Ae . tauschii			Chiese spring D			Chiese spring A			Chiese spring B		
Annotation	Gene Symol	Start	End	Gene Symol	start	end	Gene Symol	start	end	Gene Symol	start	end
protein canopy-1		92557	95727					409	3466		299	3686
F-box domain		104395	108735					3974	173415		7714	12006
60S ribosomal protein		109769	111335					9036	11928		12339	17053
hypothetical protein F775_01656		497767	501940		5225	7805		210539	216931		186306	198290
hypothetical protein TRIUR3_28025		521953	525063		33850	36982		225417	252483		203378	236032
E3 ubiquitin-protein ligase UPL4		525188	527750					241435	241830			
Minor histocompatibility antigen H13											245724	250223
Minor histocompatibility antigen H13		545148	551190		45134	51394		292703	298764		272102	277989
Glucan endo-1,3-beta-glucosidase 3		633728	638943		82825	87061		385619	390993		308802	312640
pentatricopeptide repeat-containing protein		640747	642820		87834	90669		391220	393671		314256	330245
F-box/FBD/LRR-repeat protein		667099	669661		114039	116600					352384	357710
RPM1											576772	583876
F-box/FBD/LRR-repeat protein		675390	677475		122376	126946					639698	643840
F-box/FBD/LRR-repeat protein		678045	680271		184946	186036						
F-box/FBD/LRR-repeat protein		751389	755472					669622	672868			
Adenosylhomocysteinase		755551	777421		202363	205691						
F-box/FBD/LRR-repeat protein		836363	840228		266734	271961		733298	736457		717334	720972
Chloroplastic		849400	852898		287670	289442		791123	793138		762820	767710
chaperone protein DnaJ								794801	795671			
Glutamate receptor 3.1	GLR-D^{t}1	855554	859160	GLR-D1	290816	297037	GLR-A1	801093	805487	GLR-B1	768386	772142
Glutamate receptor 3.1	GLR-D^{t}2	966212	969981	GLR-D2	421554	427327	GLR-A2	814531	820275	GLR-B2	832947	838631
hypothetical protein TRIUR3_04005								897112	903671			
α-gliadin	α-D^{t}1	1001409	1002271	α-D1	458540	459403	α-A1	995875	996759	α-B1	870320	871228
α-gliadin							α-A2	1034005	1034961	α-B2	921475	922359
α-gliadin										α-B3	959814	960806
α-gliadin										α-B4	965651	966196
α-gliadin										α-B5	966197	966633
Glutamate receptor 3.1	GLR-D^{t}3	1007729	1011532	GLR-D3	463781	468764	GLR-A3	1128596	1133528	GLR-B3	983220	986860
α-gliadin	α-D^{t}2	1055096	1055342	α-D2	512055	512305	α-A3	1182025	1182889	α-B6	1004951	1005907
α-gliadin	α-D^{t}3	1061229	1062082				α-A4	1218752	1219601	α-B7	1016169	1017110
α-gliadin	α-D^{t}4	1095454	1096297	α-D4	518180	519055	α-A5	1314262	1315182	α-B8	1042234	1043184
α-gliadin	α-D^{t}5	1137283	1138187	α-D5	553251	554177	α-A6	1423738	1424592	α-B9	1053446	1054387
α-gliadin	α-D^{t}6	1163469	1164361	α-D6	578406	579290	α-A7	1517352	1518224	α-B10	1064645	1065586
α-gliadin	α-D^{t}7	1186283	1187124	α-D7	602940	603791	α-A8	1581439	1582302	α-B11	1310666	1311544
α-gliadin	α-D^{t}8	1210543	1211405	α-D8	627665	628543	α-A9	1633882	1634775	α-B12	1332444	1333340
α-gliadin	α-D^{t}9	1242375	1243652	α-D9	659232	660092	α-A10	1689164	1690003	α-B13	1369120	1370001
α-gliadin	α-D^{t}10	1265184	1266037	α-D10	684038	684907				α-B14	1381550	1382425
α-gliadin	α-D^{t}11	1396535	1397817	α-D11	814221	815366				α-B15	1403073	1403945
α-gliadin	α-D^{t}12	1547554	1548412	α-D12	904919	905767				α-B16	1418791	1419681
α-gliadin										α-B17	1429202	1430092
α-gliadin										α-B18	1439610	1440494
α-gliadin										α-B19	1465984	1466683
α-gliadin										α-B20	1494099	1494914
α-gliadin										α-B21	1503116	1503928
α-gliadin										α-B22	1523686	1606266
α-gliadin										α-B23	1630120	1630998
α-gliadin										α-B24	1635342	1635572
Glutamate receptor 3.1	GLR-D^{t}4	1686662	1691883	GLR-D4	1039153	1042705	GLR-A4	1739408	1742963	GLR-B4	1667279	1672684
FKBP-type peptidyl-prolyl cis-trans isomerase 1		1692096	1693651		1043561	1044874		1743941	1745281		1672813	1674320
UTP--glucose-1-phosphate uridylyltransferase		1693974	1698030		1045218	1049278		1745624	1749592		1674684	1678697
LRR receptor-like serine/threonine-protein kinase		1706689	1709614		1056743	1060256						
autophagy-related protein 5b		1709869	1714699					1784221	1788290		1769246	1796599
autophagy-related protein 5b		1715457	1719914		1064533	1070359		1789079	1795854		1804110	1809147
autophagy-related protein 5b											1845256	1848786
protein trichome birefringence-like 12		1723285	1728123		1081013	1085814		1798743	1802183		1851349	1854749
RNA-binding protein 7											2039193	2044420
heat shock 70kDa protein 8, isoform CRA_c											2108931	2119210
nuclear cap-binding protein subunit 1											2222692	2234574
calcium-binding protein CML49											2368020	2369396
protein yippee-like At4g27745											2384604	2391732
protein trichome birefringence-like 12		1737536	1740991		1095117	1107589		1863321	1868273		2392699	2396187

Table S3. Estimation of α-gliadin transcript abundances in different developmental stages and tissues in Chinese Spring

	10WE	20AL	20SE	20 TC	30SE	30ALSE
Total α-gliadin gene reads	13392050	10936016	31592896	20866018	29630536	20236351
Total reads mapped to complete gene set	135836843	219235733	134302445	133453380	132547092	130940454
Total RNA-seq reads	221603358	375865316	227982060	218525346	227067144	225584234
% α-gliadin reads in total mapped gene reads	9.9	5.0	23.5	15.6	22.4	15.5
% α-gliadin reads in total RNA-seq reads	6.0	2.9	13.9	9.5	13.0	9.0

Note: RNA-seq data were downloaded from Pfeiffer *et al.*, (2014). 10WE –whole embryo at 10 days post anthesis, 20AL – Aleurone layer at 20 days, 20SE-seed endosperm at 20 days, 20TC- Transfer cells at 20 days, 30SE- seed endosperm at 30 days, 30ALSE- Aleurone + seed endosperm at 30 days

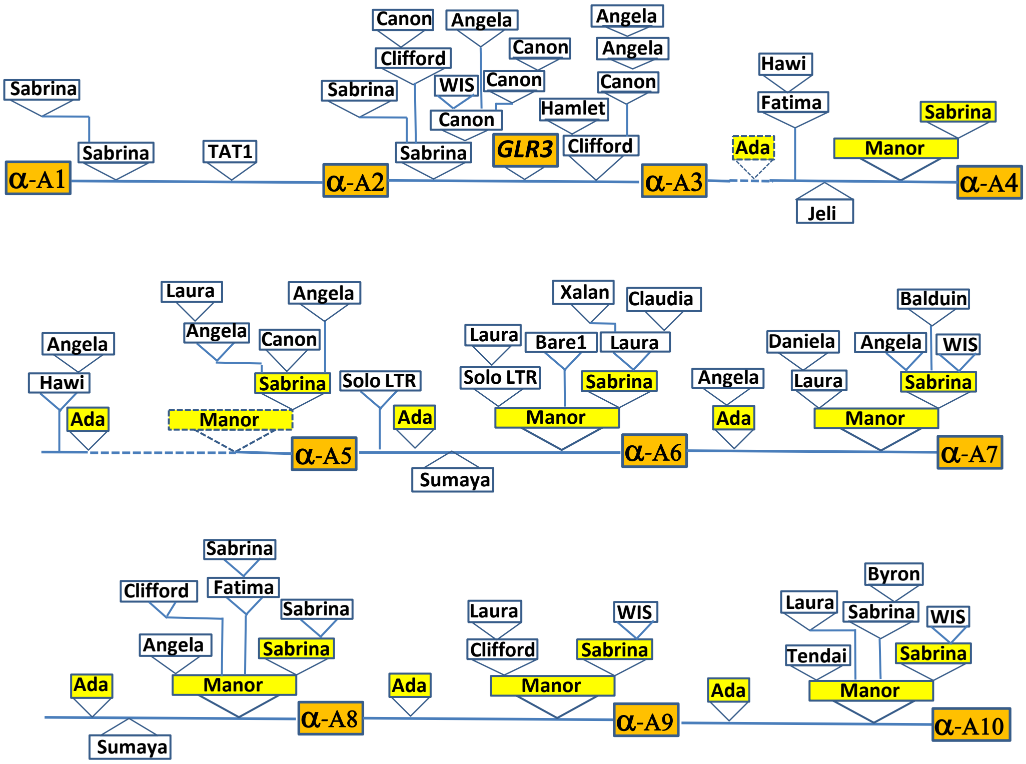

Figure S1. Genomic organization of the α-gliadin locus region in Chinese Spring A genome. The genomic sequence containing α-gliadin genes was annotated as described in Materials and Methods. α-gliadin genes are represented by boxes filled with color. Retroelements are boxed with their names, and their insertion positions are indicated by triangles or solid vertical lines. Retroelements shared in the intergenic regions between two α-gliadin genes are highlighted with yellow. Dashed lines indicate deleted sequences.

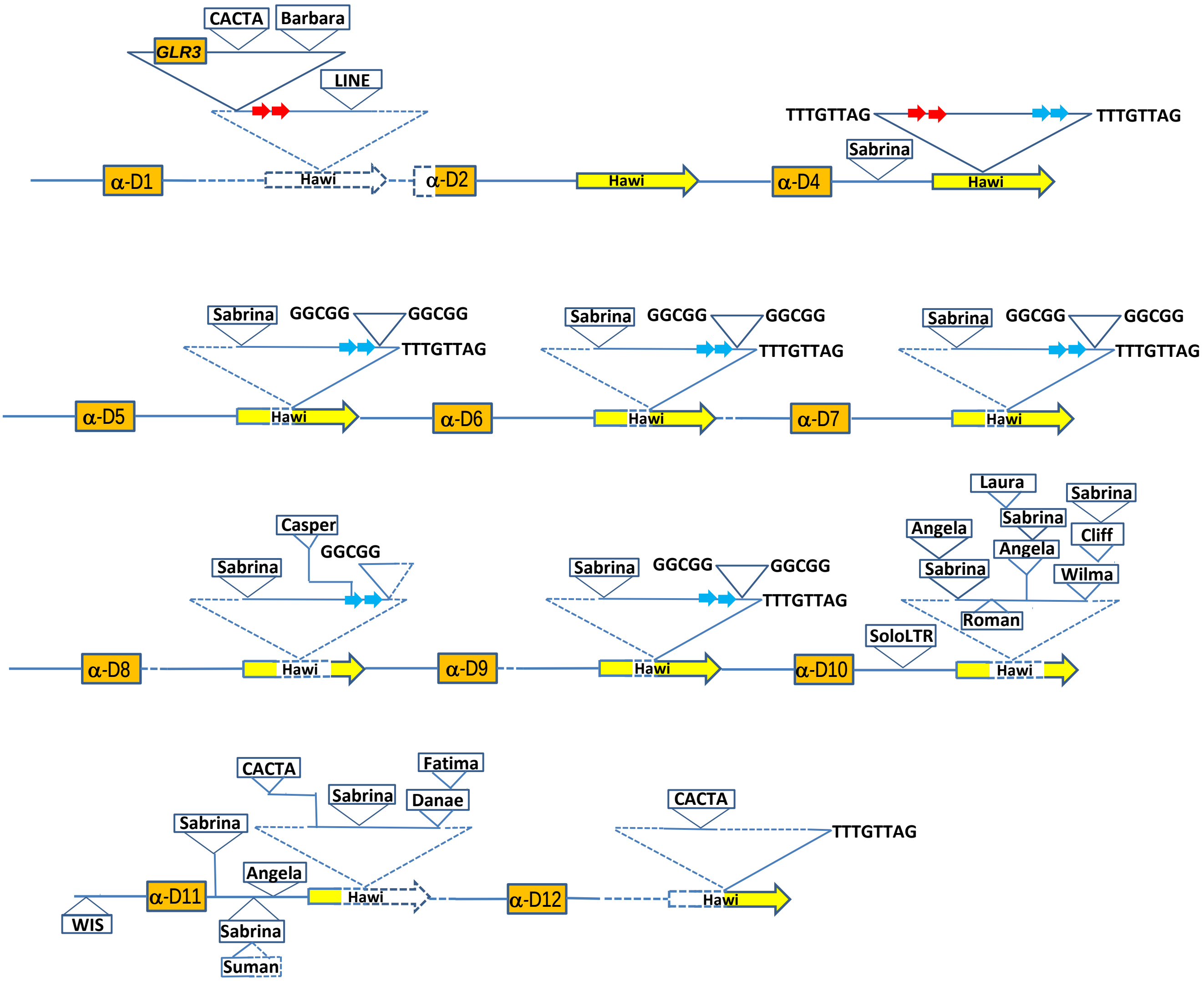

Figure S2. Genomic organization of the α-gliadin locus region in Chinese Spring D genome. The genomic sequence containing α-gliadin genes was annotated as described in Materials and Methods. α-gliadin genes are represented by boxes filled with color. *Hawi* repetitive DNA sequences are represented by yellow arrows. Two different tandem repeats in an inserted sequence and not found in the TREP repetitive element database, are indicated by the red- and blue-colored arrows. Dashed lines indicate deleted sequences. Retroelements are boxed with their names, and their insertion positions are indicated by triangles or solid vertical lines.

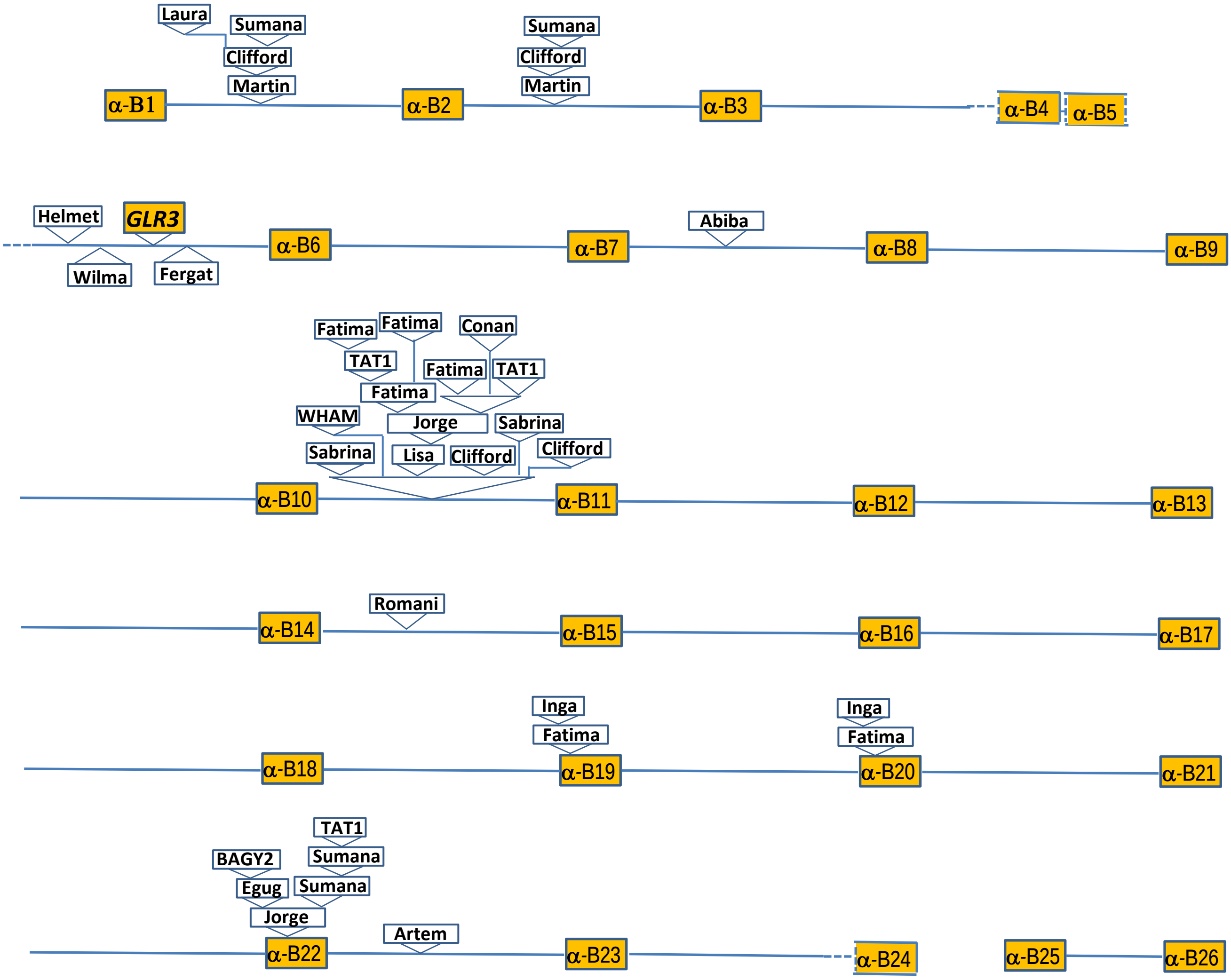

Figure S3. Genomic organization of the α-gliadin locus region in Chinese Spring B genome. The genomic sequence containing α-gliadin genes was annotated as described in Materials and Methods. α-gliadin genes are represented by boxes filled with color. Retroelements are boxed with their names, and their insertion positions are indicated by triangles or solid vertical lines. Dashed lines indicate deleted sequences.

Figure S4. Nucleotide and amino acid sequences of α-gliadin genes in Chinese Spring

α-A1 Nucleotide 885 bp

ATGAAGACTTTTCTCATCCTTGCCCTCCTTGCTATTGTGGCGACCACTGCCACAACTGCAGTTAGAGTTCCAGTGCCACAATTGCA
GCCGCAAAATCCATCTCTGCAGCAACCACAAGAGCAAGTTCCATTGGTGCAACAACAACAATTTCCAGGGCAGCAACAAACATTTC
CACCACAACAGCCATATCCGCAGCCGCAACCATTTCCAGCACAACAACCATATCCGCAGCCGCAACTATTTCCACAGCCGCAACCT
TTTCCGCCACAACTTCCATATCCGCAGCCGCAACCATTTCCCCCACAACAACCATATCCACAACCGCAAACACAGCATCTGCAACC
ACAACAACCAATTTCGCAGCAACAAGCACAACAACAACAACAACAACAACAACAACAACAAATCCTTCAACAAATTCTGCAACAAC
AACAACTGATTCCATGCAGGGATGTCATCGTCTTGCAACAACACAACATAGCGCATGAAAGCTCACAAGTATTGCAGCAAAGTAGT
TACCAAGTGTTGCAACAATTATGTTGTCAGCAGCTGCGGCAGATCCCCGAGCAGTCGCGGTGCCAAGCCATCAACAATGTCGTTCA
TGCTATTATTCTGCATCAACAACAACAACAACAAGGACAACATCAACAGTCGAGCCAGGTCTCCTACCAGCAGCCTCAGCAACAAT
ATCCATCAGGCCAGGGCTCCTTCCAGCCATCTCAGCAAAATCCACAGGCCCAGGGCTTTGTCCAACCTCAACATCTGCCCCAACTC
GAGGAAATAAGTAACCTAGCGCTGCAGACGCTACCAGCAATGTGCAATGTCTACATCCCTCCATATTGCTCGACCACCATTGCGCC
ATTTGGCATCTTCGGTACTAACTGA

α-A1 amino acid 295 aa

MKTFLILALLAIVATTATTAVRVPVPQLQPQNPSLQQPQEQVPLVQQQQFPGQQQTFPPQQPYPQPQPFPAQQPYPQPQLFPQPQP
FPPQLPYPQPQPFPPQQPYPQPQTQHLQPQQPISQQQAQQQQQQQQQQQILQQILQQQQLIPCRDVIVLQQHNIAHESSQVLQQSS
YQVLQQLCCQQLRQIPEQSRCQAINNVVHAIILHQQQQQQGQHQQSSQVSYQQPQQQYPSGQGSFQPSQQNPQAQGFVQPQHLPQL
EEISNLALQTLPAMCNVYIPPYCSTTIAPFGIFGTN.

α-A2 nucleotide 957 bp

ATGAAGACATTTCTCATCATTTCCCTCCTTGCTATCGTGGCGACCACCGCCACAACTGCAGTTAGAGTTCCAGTGCCACAATTGCA
GCCGCAAAATCCATCTCTGCAACAACCACAAGAGCAAGTTCCATTGGTGCAACAACAACAATTTCCAGGGCAGCAACAAACATTTC
CACCACAACAACCATATCCGCAGCCGCAACCATTTCCAGGACAACAACCATATCCGCAGCCGCAACTATTTCCACAGCCGCAACCT
TTTCCGCCACAACTTCCATATCCGCAGCCGCAACCATTTCCCCCACAACAACCATATCCACAACCGCAAACACATCATCTGCAACC
ACAACAACCAATTTCGCAGCAACAAGTACAACAACAACAACAACAACAACAACAACAACAACAACAACAACAACAACAACAACAAC
AACAACAACAACTACTACTACAACAACAAATCCTTCAACAAATTCTGCAACAACAACAACTGATTCCATGCAGGGATGTCATCGTC
TTGCAACAACACAACATAGCGCATGAAAGCTCACAAGTATTGCAGCAAAGTAGTTACCAAGTGTTGCAACAATTATGTTGTCAGCA
GCTGCGGCTGATCCCCGAGCAGTCGCGGTGCCAAGCCATCCACAATGTCGTTCATGCTATTATTCTACATCAACAACAACAACAAC
AACAACAACAACAACAACAAGGACAACATCAACCGTCGAGCCAGGTCTCCTACCAGCAGCCTCAGCAACAATATCCATCAGGCCAG
GGCTCCTTCCAGCCATCTCAGCAAAATCCACAGGCCCAGGGCTTTGTCCAACCTCAACATCTGCCCCAACTCGAGGAAATAAGTAA
CCTAGCGCTGCAGACGCTACCAGCAATGTGCAATGTCTACATCCCTCCATATTGCTCGACCACCATTGCGCCATTTGGCATCTTCG
GTACTAACTGA

α-A2 amino acid 319 aa

MKTFLIISLLAIVATTATTAVRVPVPQLQPQNPSLQQPQEQVPLVQQQQFPGQQQTFPPQQPYPQPQPFPGQQPYPQPQLFPQPQP
FPPQLPYPQPQPFPPQQPYPQPQTHHLQPQQPISQQQVQQQQQQQQQQQQQQQQQQQQQQQLLLQQQILQQILQQQQLIPCRDVIV
LQQHNIAHESSQVLQQSSYQVLQQLCCQQLRLIPEQSRCQAIHNVVHAIILHQQQQQQQQQQQQGQHQPSSQVSYQQPQQQYPSGQ
GSFQPSQQNPQAQGFVQPQHLPQLEEISNLALQTLPAMCNVYIPPYCSTTIAPFGIFGTN.

α-A3 nucleotide 865 bp

ATGAAGACCTTTCTCATCCTTTCCCTCCTTGCTATCGTGGCAACCACCGCCACAACTGCATTTAGAATTCTAGTGCCACAATTGCA
GCCACAAAATCCATCTTAGCAACAACCACAAGAGCAAGTTCCATTGGTACAACAACAGCAATTTCTAGGGCAGCAACAACCATTTC
CACCACAACAACCATATCCACAGCCGCAACCATTTCCATCACAACAACCATATCTGCAGCTGCAACCATTTCCGCAGCCGCAACCA
CCATATTCGCAGCCGCAACCATTTCGACCACAATAACCATATCTCCACAACCGCAACCACAGTATTCGCAACCACAATAACCAATT
TCGCAGCAGCAACAACAACAACAACAACAACAACAACAACAAATCCTTCAACAAATTTTGCAACAACAACTGATTCCATGCATGGA
TGTTGTATTGCAGCAACACAACATAGCGCATGGAAGATCACAAGTTTTGCAACAAAGTAGTTACCAGCTGTTGCAAGAATTGTGTT
TTCAGCACCTATGACAGATCCCTGAGCAGTCGCAGTGCCAAGCCATCCACAATGTTGTCCATGCTATTATTCTGCATCAACAACAA
AAACAACAACAACAACAACAACAAAAACAACAACCATCGAGCCAGGTCTCCTTCCAACAGCCCCAGCAACAATATCCATCAGGCCA
GGGCTCCTTCCGGCCATCTCTGCAAAATCCACAGGCCCAGGGCTCGGTCCAGCCTCAACAGCTGCCCAGTTCGAGGAAATAAGGAA

CCTAGCGCTACAGACGCTACCTGCAATGTGCAATCTCTACATCCCTCCATATTGCACCATCGCGCCATTTGGCATCTTCGGTACTA
ACTGA

α-A3 amino acid 295 aa

MKTFLILSLLAIVATTATTAFRILVPQLQPQNPS.QQPQEQVPLVQQQQFLGQQQPFPPQQPYPQPQPFPSQQPYLQLQPFPQPQP
PYSQPQPFRPQ.PYL...QPQPQYSQPQ.PISQQQQQQQQQQQQQQILQQILQQQLIPCMDVVLQQHNIAHGRSQVLQQSSYQLLQE
LCFQHL.QIPEQ...SQCQAIHNVVHAIILHQQQKQQQQQQQKQQPSSQVSFQQPQQQYPSGQGSFRPSLQNPQAQGSVQPQQLPS
S...IRNLALQTLPAMCNLYIPPYCTIAPFGIFGTN.

α-A4 nucleotide 852 bp

ATGAAGACATTTCTCATCCTTGCCCTCCTTGCTATCGTGGCGACCACCGCCACAACTGCAGTTAGAGTTCCAGTGCCACAATTGCA
GCCACAAAATCCATCTCAGCAACAGTCACAAGAGCAAGTTCCATTGGTACAACAACAACAATTTCTAGGGCAGCAACAACCATTTC
CACCACAACAACCATATCCACAGCCGCAACCATTTCCATCACAACAACCATATCTGCAGCTGCAACCATTTCCGCAGCCGCAACTA
CCATATTCGCAGCCACAACCATTTCGACCACAACAACCATATCCACAACCGCAACCACAATATTCGCAACCACAACATCCAATTTC
ACAGCAGCAGCAGCAGCAACAACAACAACAACAACAAGAACAACAAATACTTCAACAAATTTTGCAACAACAACTGATTCCATGCA
TGGATGTTGTATTGCAGCAACACAACATAGCGCATGGAAGATCACAAGTTTTGCAACAAAGTACTTACCAGCTGTTGCAAGAATTG
TGTTGTCAGCACCTATGGCAGATCCCTGAGCAGTCGCAGTGCCAGGCCATCCACAATGTTGTTCATGCTATTATTCTGCGTCAACA
ACAAAAACAACAACAACAACCATCGAGCCAGGTCTCCTTCCAACAGCCTCAGCAACAATATCCATTAGGCCAGGGCTCCTTCCGGC
CATCTCAGCAAAACCCACAGGCCCAGGGCTCTGTCCAGCCTCAACAACTGCCCCAGTTCGAGGAAATAAGGAACCTAGCGCTACAG
ACGCTACCTGCAATGTGCAATGTCTACATCCCTCCATATTGCACCATCGCGCCATTTGGCATCTTTGGTACTAACTAA

α-A4 amino acid 284 aa

MKTFLILALLAIVATTATTAVRVPVPQLQPQNPSQQQSQEQVPLVQQQQFLGQQQPFPPQQPYPQPQPFPSQQPYLQLQPFPQPQL
PYSQPQPFRPQQPYPQPQPQYSQPQHPISQQQQQQQQQQQQEQQILQQILQQQLIPCMDVVLQQHNIAHGRSQVLQQSTYQLLQEL
CCQHLWQIPEQSQCQAIHNVVHAIILRQQQKQQQQPSSQVSFQQPQQQYPLGQGSFRPSQQNPQAQGSVQPQQLPQFEEIRNLALQ
TLPAMCNVYIPPYCTIAPFGIFGTN.

α-A5 nucleotide 921 bp

ATGAAGACATTTCTCATCCTTGCCCTCCTTGCTATCGTGGCGACCACCGCCACAACTGCAGTTAGAGTTCCAGTGCCACAATTGCA
GCCACAAAATCCATCTCAGCAACAGCCACAAGAGCAAGTTCCATTGGTACAACAACAACAATTTCTAGGGCAGCAACAACCATTTC
CACCACAACAACCATATCCACAGCCGCAACCATTTCCATCACAACAACCATATCTGCAGCTGCAACCATTTCCGCAGCCGCAACTA
CCATATTCGCAGCCACAACCATTTCGACCACAACAACCATATCCACAACCGCAACCACAGTATTCGCAACTACAACAACCAATTTC
ACAGCAGCAGCAGCAGCAGCAACAACAACAACAACAACAACAACAACAACAACAACAACAACAACAACAACAACAACAACAACAAC
AACAACAACAACAACAAGAACAACAAATCCTTCAACAAATTTTGCAACAACAACTAATTCCATGCATGGATGTTGTATTACAGCAA
CACAACATAGCGCATGGAAGATCACAAGTTTTGCAACAAAGTACTTACCAGCTGTTGCAAGAATTGTGTTGTCAGCACCTATGGCA
GATCCCTGAGCAGTCGCAGTGCCAGGCCATCCACAATGTTGTTCATGCTATTATTCTGCATCAACAACAAAAACAACAACAACAAC
AACCATCGAGCCAGGTCTCCTTCCAACAGCCTCAACAACAATATCCATTAGGCCAGGGCTCCTTCCGGCCATCTCAGCAAAACCCA
CAGGCCCAGGGCTCTGTCCAGCCTCAACAACTGCCCCAGTTCGAGGAAATAAGGAACCTAGCGCTACAGACGCTACCTGCAATGTG
CAATGTCTACATCCCTCCATATTGCACCATCGTGCCATTTGGCATCTTCGGTACTAACTGA

α-A5 amino acid 307 aa

MKTFLILALLAIVATTATTAVRVPVPQLQPQNPSQQQPQEQVPLVQQQQFLGQQQPFPPQQPYPQPQPFPSQQPYLQLQPFPQPQL
PYSQPQPFRPQQPYPQPQPQYSQLQQPISQQQQQQQQQQQQQQQQQQQQQQQQQQQQQQQQQQEQQILQQILQQQLIPCMDVVLQQ
HNIAHGRSQVLQQSTYQLLQELCCQHLWQIPEQSQCQAIHNVVHAIILHQQQKQQQQQPSSQVSFQQPQQQYPLGQGSFRPSQQNP
QAQGSVQPQQLPQFEEIRNLALQTLPAMCNVYIPPYCTIVPFGIFGTN.

α-A6 nucleotide 855 bp

ATGAAGACCTTTCTCATCCTTGCCCTCCTTGCTATCGTGGCGACCACCGCCACAACTGCAGTTAGAGTTCCAGTGCCACAATTGCA
GCCACAACATCCATCTCAGCAACAGCCACAAGAGCAAGTTCCATTGGTACAACAACAACAATTTCTAGGGCAGCAACAACCATTTC
CACCACAACAACCATATCCACAGCCGCAACCATTTCCATCACAACAACCATATCTGCAACTACAACCATTTCCGCAGCCGCAACTA

CCATATTCGCAGCCACAACCATTTCGACCACAACAACCATATCCACAACCGCAACCACAGTATTCGCAACCACAACAACCAATTTC
ACAGCAGCAGCAGCAGCAGCAACAACAACAACAACAACAACAACAACAAATCCTTCAACAAATTTTGCAACAACAACTGATTCCAT
GCATGGATGTTGTATTGCAGCAACACAACATAGCGCATGGAAGATCACAAGTTTTGCAACAAAGTACTTACCAGCTGTTACAAGAA
TTGTGTTGTCAGCACCTATGGCAGATCCCTGAGCAGTCGCAGTGCCAGGCCATCCACAATGTTGTTCATGCTATTATTCTGCATCA
ACAACAAAAACCACAACAACAACCATCGAGCCAGGTCTCCTTCCAACAGCCTCTGCAACAATATCCATTAGGCCAGGGCTCCTTCC
GGCCATCTCAGCAAAACCCACAGGCCCGGGGCTCTGTCCAGCCTCAACAACTGCCCCAGTTCGAGGAAATAAGGAACCTAGCGCTA
CAGACGCTACCCGCAATGTGCAATGTCTACATCCCTCCATATTGCACCATCGCGCCATTTGGCATCTTCGGTACTAACTGA

α-A6 amino acid 285 aa

MKTFLILALLAIVATTATTAVRVPVPQLQPQHPSQQQPQEQVPLVQQQQFLGQQQPFPPQQPYPQPQPFPSQQPYLQLQPFPQPQL
PYSQPQPFRPQQPYPQPQPQYSQPQQPISQQQQQQQQQQQQQQQQQILQQILQQQLIPCMDVVLQQHNIAHGRSQVLQQSTYQLLQE
LCCQHLWQIPEQSQCQAIHNVVHAIILHQQQKPQQQPSSQVSFQQPLQQYPLGQGSFRPSQQNPQARGSVQPQQLPQFEEIRNLAL
QTLPAMCNVYIPPYCTIAPFGIFGTN.

α-A7 nucleotide 873 bp

ATGAAGACCTTTCTCATCCTTGCCCTCCTTGCTATCGTGGCGACCACCGCCACAACCGCAGTTAGAGTTCCAGTGCCACAATTGCA
GCCACAAAATCCATCTCAGCAACAGCCACAAGAGCAAGTTCCATTGGTACAACAACAACAATTTCTAGGGCAGCAACAAACATTTC
CACCACAACAACCATATCCACAGCTGCAACCATTTCCATCACAACAACCATATCTACAGCTGCAACCATTTCCGCAGCCGCAACTA
CCATATTCGCAGCCACAACCATTTCGACCACAACAACCATATCCACAACCGCAACCACAGTATTCGCAACCACAACAACCAATTTC
ACAGCATCAACAGCAGCAGCAGCAACAACAACAACAACAACAACAACAACAAATCCTTCAACAAATTTTGCAACAACAACTGATTC
CATGCATGGATGTTGTATTGCAGCAACACAACATAGCGCAAGGAAGATCACAAGTTTTGCAACAAAGTACTTACCAGCTGTTGCAA
GAATTGTGTTGTCAGCACCTATGGCAGATCCCTGAGCAGTCGCAGTGCCAGGCCATCCACAATGTTGTTCATGCTATTATTCTGCA
TCAACAACAACAAAAACAACAACAACAACAACAACAACAACCATCGAGCCAGGTCTCCTTCCAACAGCCTCAGCAACAATATCCAT
TAGGCTAGGGCTCCTTCCGGCCATCTCAGCAAAACCCACAGGCCCAGGGCTCTGTCCAGCCTCAACAACTGCCCCAGTTCGAGGAA
ATAAGGAACCTAGCGCTACAGACACTACCTGCAATTTGCAATGTCTACATCCCTCCATATTGCACCATCGCGCCATTTGGCATCTT
CGGTACTAACTGA

α-A7 amino acid 291 aa

MKTFLILALLAIVATTATTAVRVPVPQLQPQNPSQQQPQEQVPLVQQQQFLGQQQTFPPQQPYPQLQPFPSQQPYLQLQPFPQPQL
PYSQPQPFRPQQPYPQPQPQYSQPQQPISQHQQQQQQQQQQQQQQQQILQQILQQQLIPCMDVVLQQHNIAQGRSQVLQQSTYQLLQ
ELCCQHLWQIPEQSQCQAIHNVVHAIILHQQQQKQQQQQQQQPSSQVSFQQPQQQYPLG.GSFRPSQQNPQAQGSVQPQQLPQFEE
IRNLALQTLPAICNVYIPPYCTIAPFGIFGTN.

α-A8 nucleotide 864 bp

ATGAAGACCTTTCTCATCCTTGCCCTcCCTGCTATCGTGGCGACCACCGCCACAACTGCAGTTAGAGTTCCAGTGCCACAATTGCA
GCCACAAAATCCATCTCAGCAACAGCCACAAGAGCAAGTTCCATTGGTACAACAACAACAATTTCTAGGGCAGCAACAACCATTTC
CACCACAACAACCATATCCACAGCCGCAACCATTTCCATCACAACAACCATATCTGCAGCTGCAACCATTTCCGCAGCCGCAACTA
CCATATTCGCAGCCACAACCATTTCGACCACAACAACCATATCCACAACCGCAACCACAGTATTCGCAACCACAACAACCAATTTC
ACAGCAGCAGCAGCAGCAGCAACAACAACAACAACAACAACAACAACaacaAAAGCAAATCCTTCAACAAATTTTGCAACAACAAC
TGATTCCATGCATGGATGTTGTATTGCAGCAACACAACATAGCGCATGGAAGATCACAAGTTTTGCAACAAAGTACTTACCAGCTG
TTGCAAGAATTGTGTTGTCAGCACCTATGGCAGATCCCTGAGCAGTCGCAGTGTCAGGCCATCCAAAATGTTGTTCATGCTATTAT
TCTGCATCAACAACAAAAACAACAACAACAACCATCGAGCCAGGTGTCCTTCCAACAGCCTCTGCAACAATATCCATTAGGCCAGG
GCTCCTTCCGGCCATCTCAGCAAAACCCACAGGACCAGGGCTCTGTCCAGCCTCAACAACTGCCCCAGTTCGAGGAAATAAGGAAC
CTAGCGCTACAGATGCTACCTGCAATGTGCAATGTCTACATCCCTCCATATTGCACAATCGCGCCATTTGGCATCTTCGGTACTAA
CTGA

α-A8 amino acid 288 aa

MKTFLILALPAIVATTATTAVRVPVPQLQPQNPSQQQPQEQVPLVQQQQFLGQQQPFPPQQPYPQPQPFPSQQPYLQLQPFPQPQL
PYSQPQPFRPQQPYPQPQPQYSQPQQPISQQQQQQQQQQQQQQQQQQKQILQQILQQQLIPCMDVVLQQHNIAHGRSQVLQQSTYQL
LQELCCQHLWQIPEQSQCQAIQNVVHAIILHQQQKQQQQPSSQVSFQQPLQQYPLGQGSFRPSQQNPQDQGSVQPQQLPQFEEIRN
LALQMLPAMCNVYIPPYCTIAPFGIFGTN.

α-A9 nucleotide 894 bp

ATGAAGACCTTTCTCATCCTTGCCCTCCTTGCTATCGTGGCGACCACCGCCACAACTGCAGTTAGAGTTCCAGTGCCACAATTGCA
GCCACAAAATCCATCTCAGCAACAGCCACAGGAGCAAGTTCCAGTGGTACAACAACAACAATTTCTAGGGCAGCAACAACCATTTC
CACCACAACAACCATATCCACAGCCGCAACCATTTCCATCACAACAACCATATCTGCAGCTGCAACCATTTCCGCAGCCGCAACTA
CCATATTCGCAGCCACAACCATTTCAACCACAACAACCATATCCACAACCGCAACCACAGTATTCGCAACCACAACAACCAATTTC
ACAGCAGCAGCAGCAGCAGCAGCAGCAGCAACAACAACAACAACAACAACAACAACAACAACAACCACAACAAATCCTTCAACAAA
TTTTGCAACAACAACTGATTCCATGCATGGATGTTGTATTGCAGCAACACAACATAGCGCAAGGAAGATCACAAGTTTTGCAACAA
AGTACTTACCAGCTGTTGCAAGAATTGTGTTGTCAGCACCTATGGCAGATCCCTGAGCAGTCGCAGTGCCAGGCCATCCACAATGT
TGTTCATGCTATTATTCTGCATCAACAACAAAAACAACAACAACAACAACAACAACAACAACCATCGAGTCAGGTCTCCTTCCAAC
AGCCTCAGCAACAATATCCATTAGGCCAGGGCTCCTTCCGGCCATCTCAGCAAAACCCACAGGCCCAGGGCTCTGTCCAGCCTCAA
CAACTGCCCCAGTTCGAGGAAATAAGGAACCTAGCGCTACAGACACTACCTGCAATTTGCAATGTCTACATCCCTCCATATTGCAC
CATCGCGCCATTTGGCATCTTCGGTACTAACTGA

α-A9 amino acid 298 aa

MKTFLILALLAIVATTATTAVRVPVPQLQPQNPSQQQPQEQVPVVQQQQFLGQQQPFPPQQPYPQPQPFPSQQPYLQLQPFPQPQL
PYSQPQPFQPQQPYPQPQPQYSQPQQPISQQQQQQQQQQQQQQQQQQQQPQQILQQILQQQLIPCMDVVLQQHNIAQGRSQVLQQ
STYQLLQELCCQHLWQIPEQSQCQAIHNVVHAIILHQQQKQQQQQQQQQPSSQVSFQQPQQQYPLGQGSFRPSQQNPQAQGSVQPQ
QLPQFEEIRNLALQTLPAICNVYIPPYCTIAPFGIFGTN.

α-A10 nucleotide 840 bp

ATGAAGACCTTTCTCATCCTTGCCCTCCTTGCTATCGTGGCGACCACCGCCACAACTGCAGTTAGAGTTCCAGTGCCACAATTGCA
GCCACAAAATCCATCTCAGCAACAGCCACAAGAGCAAGTTCCATTGGTACAACAACAACAATTTCTAGGGCAGCAACAACCATTTC
CACCACAACAACCATATCCACAGCCGCAACCATTTCCATCACAACAACCATATCTGCAGCTGCAACCATTTCCGCAGCCGCAACTA
CCATATTCGCAGCCACAACCATTTCGACCACAACAACCATATCCACAACCGCAACCACAGTATTCGCAACCACAAGAACCAATTTC
ACAGCAGCAGCAACAACAACAACAACAACAACAAATCCTTCAACAAATTTTGCAACAACAACTGATTCCATGCATGGATGTTGTAT
TGCAGCAACACAACATAGCGCATGGAAGATCACAAGTTTTGCAACAAAGTACTTACCAGCTATTGCAAGAATTGTGTTGTCAGCAC
CTATGGCAGATCCCTGAGCAGTCGCAGTGCCAGGCCATCCAAAATGTTGTTCATGCTATTATTCTGCATCAACAACAAAAACAACA
ACAACAACCATCGAGCCAGGTCTCCTTCCAACAGCCTCTGCAACAATATCCATTAGGCCAGGGCTCCTTCCGGCCATCTCAGCAAA
ACCCACAGGACCAGGGCTCTGTCCAGCCTCAACAACTGCCCCAGTTCGAGGAAATAAGGAACCTAGCGCTACAGACGCTACCTGCA
ATGTGCAATGTCTACATCCCTCCATATTGCACCATCGCGCCATTTGGCATCTTCGGTACTAACTGA

α-A10 amino acid 280 aa

MKTFLILALLAIVATTATTAVRVPVPQLQPQNPSQQQPQEQVPLVQQQQFLGQQQPFPPQQPYPQPQPFPSQQPYLQLQPFPQPQL
PYSQPQPFRPQQPYPQPQPQYSQPQEPISQQQQQQQQQQQILQQILQQQLIPCMDVVLQQHNIAHGRSQVLQQSTYQLLQELCCQH
LWQIPEQSQCQAIQNVVHAIILHQQQKQQQQPSSQVSFQQPLQQYPLGQGSFRPSQQNPQDQGSVQPQQLPQFEEIRNLALQTLPA
MCNVYIPPYCTIAPFGIFGTN.

α-B1 Nucleotide 909 bp

ATGAAGACCTTTCTCATCTTTTCCCTCCTTGCTATCGTGGCGACCACTGCAACAACTGCAGTTAGAGTTCCAGTGCCACAATTGCA
GCCGCAAAATCCATCTCAGCAACAACCACAAGAGCAAGTTCCGTTGGTGCAACAACAACAATATCCAGGGCAGCAACAACCATTTC
CACCACAACAACCATATCCGCAGCCGCAACCATTTCCATCACAACAACCATTTCTGCAGCCGCAACCATTTCTGCTACAACTACCA
TATCCGCAGCCACAACCATTTCCACCACAACAACCATATCCACAACCGCAACCACAGTATCCGCAACCACAACAACCAATTTCACA
GCAACAAGCGCAACAAGCACAACAACAACAACAACAACAACAACAACAATAACAATAACAACAACAACAAATCCTTCAACAAATTC
TGCAACAACAACAACTGATTCCATGCAGGGATGTCGTCTTGCAACAACACAACATAGCGCATGCAAGCTCACAAGTATTGCAACAA
AGTAGTTACCAACTGTTGCAACAATTATGTTGTCAACGGTTGTGGCAGATCCCCGAGCAGTCGCGGTGCCAAGCCATCCACAATGT
CGTTCATGCTATTATTCTGCAACAACAACAACAACAACAACAACAACAACAACAACAACAACAACAACAACCGTCGAGCCAGGTCT
CCTACCAGCAGCCTCAGCAACAATATCCATCGGGCCAGGGCTCCTTCCAGCCATCTCAGCAGAACCCATAGGCCCAGGGCTCTGTC
CAGTCTCAACAACTTCCCCAGTTCGAGGAAATAAGGAACCTAGTGCTGCAGACGCTACCGGCAATGTGCAATGTCTACATCCCTCC
ATATTGCTCGACCACCATTGCGCCATCTGGCATCTTCGGTACTAACTGA

α-B1 amino acid 303 aa

MKTFLIFSLLAIVATTATTAVRVPVPQLQPQNPSQQQPQEQVPLVQQQQYPGQQQPFPPQQPYPQPQPFPSQQPFLQPQPFLLQLP
YPQPQPFPPQQPYPQPQPQYPQPQQPISQQQAQQAQQQQQQQQQQ.Q.QQQQILQQILQQQQLIPCRDVVLQQHNIAHASSQVLQQ
SSYQLLQQLCCQRLWQIPEQSRCQAIHNVVHAIILQQQQQQQQQQQQQQQQQPSSQVSYQQPQQQYPSGQGSFQPSQQNP.AQGSV
QSQQLPQFEEIRNLVLQTLPAMCNVYIPPYCSTTIAPSGIFGTN.

α-B2 nucleotide 885 bp

ATGAAGACCTTTCTCATCTTTTCCCTCCTTGCTATCGTGGCGACCACTCCAACAACTGCAGTTAGATTTCCAGTGCCACAATTGCA
GCCGCAAAATCCATCTCAGCAACAACCACAAGAGCAAGTTCCGTTGGTGCAACAACTACAATATCCAAGGCAACAACAACCATTTC
CACCACAACAGCCATATCCGCAGCCGCAACCATTTCCATCACAACAACCATTGCCGCAACGGCAACCATTTCTGCCACAACTACCA
TATCCGCAGCCACAACCATTTCCACCACAACAACCATATCCACAACCGCAACCACAGTATCCACAACCACAACAACCAATTTCACA
GCAACAAGCACAATAAGCACAACAACAACAACAACAACAACAACAACAACAACAACAACAAATCCTTCAACAAATTCTGCAACAAC
AACAACTAATTCCATGCAGGGATGTCGTCTTGCAACAACACAACATAGCGCATGCAAGCTCACAAGTATTGCAACAAAGTAGTTAC
CAACTGTTGCAACAATTATGTTGTCAACGGTTGTGGCAGATCCCCGAGCAGTCGCGGTGCCAAGCCATCCACAATGTTGTTCATGC
TATTATTCTGCAACAACAACAACAACAACAACAACAACAACAACAACCGTCGAGCCAGGTCTCCTACCAGCAGCCTCAGCAACAAT
ATCCATCGGGCCAGGGCTCCTTCCAGCCATCTCAGCAGAACCCATAGGCCTAGGGCTCTGTCCAGTCTCAACAACTTCCCCAGTTC
GAGGAAATAAGGAACCTAGCGCTGCAGACGCTACCGGCAATGTGCAATGTCTACATCCCTCCATATTGCTCGACCACCATTGCGCC
ATCTGGCATCTTCGGTACTAACTGA

α-B2 amino acid 295 aa

MKTFLIFSLLAIVATTPTTAVRFPVPQLQPQNPSQQQPQEQVPLVQQLQYPRQQQPFPPQQPYPQPQPFPSQQPLPQRQPFLPQLP
YPQPQPFPPQQPYPQPQPQYPQPQQPISQQQAQ.AQQQQQQQQQQQQQQILQQILQQQQLIPCRDVVLQQHNIAHASSQVLQQSSY
QLLQQLCCQRLWQIPEQSRCQAIHNVVHAIILQQQQQQQQQQQQPSSQVSYQQPQQQYPSGQGSFQPSQQNP.A.GSVQSQQLPQF
EEIRNLALQTLPAMCNVYIPPYCSTTIAPSGIFGTN.

α-B3 nucleotide 993 bp

ATGAAGACCTTTCTCATCCTTTCCCTCCTTGCTATCGTGGCGACCACTGCCACAACTGCAGTTAGATTTCCAGTGCCACAATTGCA
GCCGCAAAATCCATCTCAGCAACAACCACAAGAGCAAGTTCCGTTGGTGCAACAACTACAATATCCAGGGCAACAACAACCATTTC
CACCACAACAGCCATATCCGCAGCCGCAACCATTTCCATCACAACAACCATTGCCGCAACCGCAACCATTTCTGCCACAACTACCA
TATCCGCAGCCACAACCATTTCCACCACAACAACCATATCCACAACCGCAACCACAGTATCCGCAACCACAACAACCAATTTCACA
GCAACAAGCACAACAAGCACAACAACAACAACAACAACAACAACAACAACAACAACAACAACAACAACAAATCCTTCAACAAATTC
TGCAACAACAACAACTGATTCCATGCAGGGATGTCGTCTTGCAACAACACAACATAGCGCATGCAAGCTCACAAGTATTGCAACAA
AGTAGTTACCAACTGTTGCAACAATTATGTTGTCAACGTTTGTGGCAGATCCCCGAGCAGTCGCGGTGCCAAGCCATCCACAATGT
CGTTCATGCTATTATTCTGCAACAACAACAACAACAACAACAACAACAACAACAACAACAACAACAACAACAACAACAACAACAAC
AACAACAACAACAACAACAACAACAACAACAACAACAACAACAACAACAACAACAACAACAACAACAACAACCGTCGAGCCAGGTCTCC
TACCAGCAGCCTCAGCAACAATATCCATCGGGCCAGGGATCCTTCCAGCCATCTCAGCAGAACCCACAGGCCCAGGCCTCTGTCCA
GCCTCAACAACTTCCCCAGTTCGAGGAAATAAGGAATCTAGCACGGCAGACGCTACCGGCAATGTGCAATGTCTACATCCCTCCAT
ATTGCTCGACCACCATTGCGCCATCTGGCATCTTTGGTACTAACTGA

α-B3 amino acid 331 aa

MKTFLILSLLAIVATTATTAVRFPVPQLQPQNPSQQQPQEQVPLVQQLQYPGQQQPFPPQQPYPQPQPFPSQQPLPQPQPFLPQLP
YPQPQPFPPQQPYPQPQPQYPQPQQPISQQQAQQAQQQQQQQQQQQQQQQQQILQQILQQQQLIPCRDVVLQQHNIAHASSQVLQQ
SSYQLLQQLCCQRLWQIPEQSRCQAIHNVVHAIILQQQQQQQQQQQQQQQQQQQQQQQQQQQQQQQQQQQQQQQQQQQQQPSSQVS
YQQPQQQYPSGQGSFQPSQQNPQAQASVQPQQLPQFEEIRNLARQTLPAMCNVYIPPYCSTTIAPSGIFGTN.

α-B4 nucleotide 546 bp

CAACAACAACAACAACAACAAATCCTTCAACAAATTCTTCAACAAATTCTGCAACAACAACAACAACTGATTCCATGCAGGGATGT
CGTCTTGAAACAACACAACATAGCGCATGCAAGCTCACAAGTATTGCAACAAAGTAGTTACCAACTATTGCAACAATTATGTTGTC
AACGGTTGTGGCAGATCCCCGAGCAGTCGCGGTGCCAGGCCATCCACAATGTCGTCCATGCTATTATTCTGCAACAACAACAACAA
CAACAACAACAACAACAACAACAACAACAACAACAACAACAACAACAACAACCGTCGAGCCAGATCTCCCTCCAGCAGCCTCAACA
ACAGTATCCATCAGGCCAGGGCTCCTTCCAGCCATCTCAGCAAAACCCACAGGCCCAGGGCTCTGTCCAGCCTCAACAACTGCCCC
AGTTCGAGGAAATAAAGAACCTAGCGCTGCAGACGCTACCGGCAATGTGCAATTTCTACATCCCTCCATATTGCTCGACCACCATT
GCGCCATTTGGCATCTTCGGTACTAACTGA

α-B4 amino acid 182 aa

QQQQQQQILQQILQQILQQQQLIPCRDVVLKQHNIAHASSQVLQQSSYQLLQQLCCQRLWQIPEQSRCQAIHNVVHAIILQQQQQ
QQQQQQQQQQQQQQQQQPSSQISLQQPQQQYPSGQGSFQPSQQNPQAQGSVQPQQLPQFEEIKNLALQTLPAMCNFYIPPYCSTTI
APFGIFGTN.

α-B5 nucleotide 437 bp

TGATTCCATGCAGGGATGTCGTCTTGAAACAACACAACATAGCGCATGCAAGCTCACAAGTATTGCAACAAAGTAGTTACCAACTA
TTGCAACAATTATGTTGTCAACGGTTGTGGCAGATCCCCGAGCAGTCGCGGTGCCAGGCCATCCACAATGTCGTCCATGCTATTAT
TCTGCAACAACAACAACAACAACAACAACAACAACAACAACAACAACAACAACAACAACAACAACAACAACCGTCGAGCCAGATCT
CCCTCCAGCAGCCTCAACAACAGTATCCATCAGGCCAGGGCTCCTTCCAGCCATCTCAGCAAAACCCACAGGCCCAGGGCTCTGTC
CAGCCTCAACAACTGCCCCAGTTCGAGGAAATAAAGAACCTAGCGCTGCAGACGCTACCGGCAATGTGCAATTTCTACATCCCTCC
ATATTGC

α-B5 Amino acid 145 aa

IPCRDVVLKQHNIAHASSQVLQQSSYQLLQQLCCQRLWQIPEQSRCQAIHNVVHAIILQQQQQQQQQQQQQQQQQQQQQPSSQIS
LQQPQQQYPSGQGSFQPSQQNPQAQGSVQPQQLPQFEEIKNLALQTLPAMCNFYIPPYC

α-B6 nucleotide 957 bp

ATGAAGACCTTTCTCATCCTTGCCCTCGTGGCGACCACCGCCACAACTGCAGTTAGAGTTCCAGTGCCACAATTGCAGCCACAAAA
TCCATCTCAGCAACAGCCACAAGAGCAAGTTCCATTGGTACAACAACAACAATTTCCAGGGCAGCAACAACAATTTCCACCACAAC
AGCCATATCCGCAGCCGCAACCATTTCCATCACAACAACCATATCTGCAGCTGCAACCATTTCCGCAGCCGCAACCATTTCTGCCA
CAACTACCATATCCGCAGCCGCAATCATTTCCACCACAACAACCATATCCACAACAGCGACCAAAGTATCTACAACCACAACAACC
AATTTCGCAGCAACAAGCACAACAACAACAACAACAACAACAACAACAACAACAACAACAACAACAACAACAACAAATCCTTCAAC
AAATTTTGCAACAACAACTGATTCCATGCAGGGATGTTATCTTGCAACAACACAACATAGCGCATGCAAGCTCACAAGTTTTGCAA
CAAAGTACTTACCAGCTATTGCAACAATTGTGTTGTCAACAACTGTTGCAGATCCCTGAGCAGTCGAGGTGCCAAGCCATACATAA
TGTTGTTCATGCTATTATTATGCATCAACAAGAACAACAACAACAGTTGCAACAACAACAACAACAGCAACTGCAACAACAACAAC
AACAACAACAACAACAACAACAACAACAACAACCGTCAAGCCAGGTCTCCTTCCAACAGCCTCAGCAGCAATATCCATCAAGCCAG
GTCTCCTTCCAGCCATCTTAGCTAAACCCACAAGCTCAGGGCTCCGTCCAACCTCAACAACTGCCCCAGTTCGCGGAAATAAGGAA
CCTAGCGCTACAGACGCTACCTGCAATGTGCAATGTCTACATCCCTCCACATTGCTCGACCACCATTGCGCCATTTGGCATCTTTG
GTACCAACTGA

α-B6 amino acid 319 aa

MKTFLILALVATTATTAVRVPVPQLQPQNPSQQQPQEQVPLVQQQQFPGQQQQFPPQQPYPQPQPFPSQQPYLQLQPFPQPQPFLP
QLPYPQPQSFPPQQPYPQQRPKYLQPQQPISQQQAQQQQQQQQQQQQQQQQQQQILQQILQQQLIPCRDVILQQHNIAHASSQVLQ
QSTYQLLQQLCCQQLLQIPEQSRCQAIHNVVHAIIMHQQEQQQQLQQQQQQQLQQQQQQQQQQQQQQQPSSQVSFQQPQQQYPSSQ
VSFQPS.LNPQAQGSVQPQQLPQFAEIRNLALQTLPAMCNVYIPPHCSTTIAPFGIFGTN.

α-B7 nucleotide 942 bp

ATGAAGACCTTTCTCATCCTTGCCCTCCTTGCTATCGTGGCGACCACCGCCACAACTGCAGTTAGAGTTCCAGTGCCACAATTGCA
GCCACAAAATCCATCTCAGCAACAGCCACAAGAGCAAGTTCCATTGGTACAACAACAACAATTTCCAGGGCAGCAACAACAATTTC
CACCACAACAGCCATATCCGCAGCCGCAACCATTTCCATCACAACAACCATATCTGCAACTGCAACCATTTCCGCAGCCGCAACCA
TTTCCGCCACAACTACCATATCCGCAGCCGCAATCATTTCCACCACAACAACCATATCCACAACAGCAACCACAGTATCTACAACC
ACAACAACCAATTTCGCAGCAACAAGCACAACAACAACAACAACAACAACAACAACAACAACAACAACAACAACAAATCCTTCAACAAA
TTTTGCAACAACAACTGATTCCATGCAGGGATGTTGTCTTGCAACAACACAACATAGCGCATGCAAGCTCACAAGTTTTGCAACAA
AGTACTTACCAGCTATTGCAACAATTGTGTTGTCAACAACTGTTGCAGATCCCTGAGCAGTCGAGGTGCCAAGCCATCCATAATGT
TGCTCATGCTATTATTATGCATCAACAACAACAACAACAACAAGAACAACAACAACAGTTGCAACAACAACAACAGCAGCAACTGC
AACAACAACGACAACAACCGTCGAGCCAGGTCTCCTTCCAACAGCCTCAGCAGCAATATCCATCAAGCCAGGTCTCCTTCCAGCCA
TCTCAGCTAAACCCACAGGCTCAGGGCTCCGTCCAACCTCAACAACTGCCCCAGTTCGCGGAAATAAGGAACCTAGCGCTACAGAC
GCTACCTGCAATGTGCAATGTCTACATCCCTCCACATTGCTCGACCACCATTGCGCCATTTGGCATCTTTGGTACCAACTGA

α-B7 amino acid 314 aa

MKTFLILALLAIVATTATTAVRVPVPQLQPQNPSQQQPQEQVPLVQQQQFPGQQQQFPPQQPYPQPQPFPSQQPYLQLQPFPQPQP
FPPQLPYPQPQSFPPQQPYPQQQPQYLQPQQPISQQQAQQQQQQQQQQQQQQQILQQILQQQLIPCRDVVLQQHNIAHASSQVLQQ
STYQLLQQLCCQQLLQIPEQSRCQAIHNVAHAIIMHQQQQQQQEQQQQLQQQQQQQLQQQRQQPSSQVSFQQPQQQYPSSQVSFQP
SQLNPQAQGSVQPQQLPQFAEIRNLALQTLPAMCNVYIPPHCSTTIAPFGIFGTN.

α-B8 nucleotide 951 bp

ATGAAGACCTTTCTCATCCTTGCCCTCGTGGCGACCACCGCCACAACTGCAGTTAGAGTTCCAGTGCCACAATTGCAGCCAAAAAA
TCCATCTCAGCAACAGCCACAAGAGCAAGTTCCATTGGTACAACAACAACAATTTCCAGGGCAGCAACAACAATTTCCACCACAAC
AGCCATATCCGCAGCCGCAACCATTTCCATCACAACAACCATATCTGCAGCTGCAACCATTTCCGCAGCCGCAACCATTTCTGCCA
CAACTACCATATCCGCAGCCGCAATCATTTCCACCACAACAACCATATCCACAACAGCGACCAAAGTATCTACAACCACAACAACC
AATTTCGCAGCAACAAGCACAACAACAACAACAACAACAACAACAACAACAACAACAACAACAACAACAACAACAACAAATCCTTC
AACAAATTTTGCAACAACAACTGATTCCATGCAGGGATGTTGTCTTGCAACAACACAACATAGCGCATGCAAGCTCACAAGTTTTG
CAACAAAGTACTTACCAGCTATTGCAACAATTGTGTTGTCAACAACTGTTGCAGATCCCTGAGCAGTCGAGGTGCCAAGCCATCCA
TAATGTTGTTCATGCTATTATTATGCATCAACAAGAACAACAACAACAGTTGCAACAACAACAACAACAGCAACTGCAACAACAAC
AACAACAACAACAACAACAACAACAACCGTCAAGCCAGGTCTCCTTCCAACAGCCTCAGCAGCAATATCCATCAAGCCAGGTCTCC
TTCCAGCCATCTCAGCTAAACCCACAAGCTCAGGGCTCCGTCCAACCTCAACAACTGCCCCAGTTCGCGGAAATAAGGAACCTAGC
GCTACAGACGCTACCTGCAATGTGCAATGTCTACATCCCTCCACATTGCTCGACCACCATTGCGCCATTTGGCATCTTTGGTACCA
ACTGA

α-B8 amino acid 317 aa

MKTFLILALVATTATTAVRVPVPQLQPKNPSQQQPQEQVPLVQQQQFPGQQQQFPPQQPYPQPQPFPSQQPYLQLQPFPQPQPFLP
QLPYPQPQSFPPQQPYPQQRPKYLQPQQPISQQQAQQQQQQQQQQQQQQQQQQQQILQQILQQQLIPCRDVVLQQHNIAHASSQVL
QQSTYQLLQQLCCQQLLQIPEQSRCQAIHNVVHAIIMHQQEQQQQLQQQQQQQLQQQQQQQQQQQQPSSQVSFQQPQQQYPSSQVS
FQPSQLNPQAQGSVQPQQLPQFAEIRNLALQTLPAMCNVYIPPHCSTTIAPFGIFGTN.

α-B9 nucleotide 942 bp

ATGAAGACCTTTCTCATCCTTGCCCTCCTTGCTATCGTGGCGACCACCGCCACAACTGCAGTTAGAGTTCCAGTGCCACAATTGCA
GCCACAAAATCCATCTCAGCAACAGCCACAAGAGCAAGTTCCATTGGTACAACAACAACAATTTCCAGGGCAGCAACAACAATTTC
CACCACAACAGCCATATCCGCAGCCGCAACCATTTCCATCACAACAACCATATCTGCAACTGCAACCATTTCCGCAGCCGCAACCA
TTTCCGCCACAACTACCATATCCGCAGCCGCAATCATTTCCACCACAACAACCATATCCACAACAGCAACCACAGTATCTACAACC
ACAACAACCAATTTCGCAGCAACAAGCACAACAACAACAACAACAACAACAACAACAACAACAACAACAACAACAAATCCTTCAACAAA
TTTTGCAACAACAACTGATTCCATGCAGGGATGTTGTCTTGCAACAACACAACATAGCGCATGCAAGCTCACAAGTTTTGCAACAA
AGTACTTACCAGCTATTGCAACAATTGTGTTGTCAACAACTGTTGCAGATCCCTGAGCAGTCGAGGTGCCAAGCCATCCATAATGT
TGCTCATGCTATTATTATGCATCAACAACAACAACAACAACAAGAACAACAACAACAGTTGCAACAACAACAACAGCAGCAACTGC
ATCAACAACGACAACAACCATCGAGCCAGGTCTCCTTCCAACAGCCTCAGCAGCAATATCCATCAAGCCAGGTCTCCTTCCAGCCA
TCTCAGCTAAACCCACAGGCTCAGGGCTCTGTCCAACCTCAACAACTGCCCCAGTTCGCGGAAATAAGGAACCTAGCGCTACAGAC
GCTACCTGCAATGTGCAATGTCTACATCCCTCCACATTGCTCGACCACCATTGCGCCATTTGGCATCTTTGGTACCAACTGA

α-B9 amino acid 314 aa

MKTFLILALLAIVATTATTAVRVPVPQLQPQNPSQQQPQEQVPLVQQQQFPGQQQQFPPQQPYPQPQPFPSQQPYLQLQPFPQPQP
FPPQLPYPQPQSFPPQQPYPQQQPQYLQPQQPISQQQAQQQQQQQQQQQQQQQILQQILQQQLIPCRDVVLQQHNIAHASSQVLQQ
STYQLLQQLCCQQLLQIPEQSRCQAIHNVAHAIIMHQQQQQQQEQQQQLQQQQQQLHQQRQQPSSQVSFQQPQQQYPSSQVSFQP
SQLNPQAQGSVQPQQLPQFAEIRNLALQTLPAMCNVYIPPHCSTTIAPFGIFGTN.

α-B10 nucleotide 942 bp

ATGAAGACCTTTCTCATCCTTGCCCTCCTTGCTATCGTGGCGACCACCGCCACAACTGCAGTTAGAGTTCCAGTGCCACAATTGCA
GCCACAAAATCCATCTCAGCAACAGCCACAAGAGCAAGTTCCATTGGTACAACAACAACAATTTCCAGGGCAGCAACAACAATTTC
CACCACAACAGCCATATCCGCAGCCGCAACCATTTCCATCACAACAACCATATCTGCAACTGCAACCATTTCCGCAGCCGCAACCA
TTTCCGCCACAACTACCATATCCGCAGCCGCAATCATTTCCACCACAACAACCATATCCACAACAGCAACCACAGTATCTACAACC
ACAACAACCAATTTCGCAGCAACAAGCACAACAACAACAACAACAACAACAACAATAACAACAACAACAACAAATCCTTCAACAAA
TTTTGCAACAACAACTGATTCCATGCAGGGATGTTGTCTTGCAACAACACAACATAGCGCATGCAAGCTCACAAGTTTTGCAACAA
AGTACTTACCAGCTATTGCAACAATTGTGTTGTCAACAACTGTTGCAGATCCCTGAGCAGTCGAGGTGCCAAGCCATCCATAATGT
TGCTCATGCTATTATTATGCATCAACAACAACAACAACAACAAGAACAACAACAACAGTTGCAACAACAACAGCAGCAGCAACTGC
AACAACAATGACAACAACCGTCGAGCCAGGTCTCCTTCCAACAGCCTCAGCAGCAATATCCATCAAGCCAGGTCTTCTTCCAGCCA
TCTCAGCTAAACCCACAGGCTCAGGGCTCCGTCCAACCTCAACAACTGCCCCAGTTCGCGGAAATAAGGAACCTAGCGCTACAGAC
GCTACCTGCAATGTGCAATGTCTACATCCCTCCACATTGCTCGACCACCATTGCGCCATTTGGCATCTTTGGTACCAACTGA

α-B10 amino acid 314 aa

MKTFLILALLAIVATTATTAVRVPVPQLQPQNPSQQQPQEQVPLVQQQQFPGQQQQFPPQQPYPQPQPFPSQQPYLQLQPFPQPQP
FPPQLPYPQPQSFPPQQPYPQQQPQYLQPQQPISQQQAQQQQQQQQ.QQQQQILQQILQQQLIPCRDVVLQQHNIAHASSQVLQQ

STYQLLQQLCCQQLLQIPEQSRCQAIHNVAHAIIMHQQQQQQQEQQQQLQQQQQQQLQQQ.QQPSSQVSFQQPQQQYPSSQVFFQP
SQLNPQAQGSVQPQQLPQFAEIRNLALQTLPAMCNVYIPPHCSTTIAPFGIFGTN.

α-B11 nucleotide 879 bp

ATGAAGACCTTTCTCATCCTTGCCCTCCTTGCTATCGTGGCGACCACCACCACAACTGCAGTTAGAGTTCCAGTGCCACAATTGCA
GCCACAAAATCCATCTCAGCAACAACCACAAGAGCAAGTTCCATTGGTGCAACAACAACAATTTCTAGGGCAGCAACAACAACAAT
TTCCAGGGCAACAACAACCATTTCCACCACAACAGCCATATCCGCAGCCGCAACCATTTCTGCCACAACTACCATATCCGCAGCCG
CAACCATTTCCACCACAACAATCATATCCACAACCACAACCACAATATCCGCAACCACAACAACCAATTTCGCAGCAACAAGCACA
ACTACAACAACAACAACAACAACAACAACAACAACAACAACAAATCCTTCAACAAATTCTGCAACAACAACTGATTCCATGCAGGG
ATGTCGTCTTGCAACAACCCAATATAGCACATGCAAGCTCAAAAGTATCGCAACAAAGTTACCAACTGTTGCAACAATTATGTTGT
CTGCAACTGTGGCAGACCCCCGAGCAGTCACGGTGCCAAGCCATCCACAATGTCATTCATGCTATTATTTTGCATCATCAACAACA
ACAACAACAACAACAACAACAACAACAACAACAACAACAACCGTCGAGCCAGGTCTCCTACCAGCAGCCTCAGCAACAATATCCAT
CAGGCCAGGGCTTCTTCCAGCCATCTCAGCAAAACCCACAGGCCCAGGGCTTTGTCCAACCTCAGCAACTGCCGCAGTTCGAGGAA
ATAAGGAACCTAGCGCTGCAGACGCTACCAGCAATGTGCAATGTCTACATCCCTCCATATTGCTCGACCACCATTGCGCCATTTGG
CATCATGAGTACTAACTGA

α-B11 amino acid 293 aa

MKTFLILALLAIVATTTTTAVRVPVPQLQPQNPSQQQPQEQVPLVQQQQFLGQQQQQFPGQQQPFPPQQPYPQPQPFLPQLPYPQP
QPFPPQQSYPQPQPQYPQPQQPISQQQAQLQQQQQQQQQQQQQILQQILQQQLIPCRDVVLQQPNIAHASSKVSQQSYQLLQQLCC
LQLWQTPEQSRCQAIHNVIHAIILHHQQQQQQQQQQQQQQQQPSSQVSYQQPQQQYPSGQGFFQPSQQNPQAQGFVQPQQLPQFEE
IRNLALQTLPAMCNVYIPPYCSTTIAPFGIMSTN.

α-B12 nucleotide 897 bp

ATGAAGACCTTTCTCATCCTTGCCCTCCTTGCTATCGTGGCGACCACCACCACAACTGCAGTTAGAGTTCCAGTGCCATAATTGCA
GCCACAAAATCCATCTCAGCAACAACCACAAGAGCAAGTTCCATTGGTGCAACAACAACAATTTCTAGGGCAGCAACAACAACAAT
TTCCAGGGCAACAACAACCATTTCCACCACAACAGCCATATCCGCAGCCGCAACCATTTCTGCCACAACTACCATATCCGCAGCCG
CAACCATTTCCACCACAACAATCATATCCACAACCACAACCACAATATCCGCAACCACAACAACCAATTTCGCAGCAACAAGCACA
ACTACAACAACAACAACAACAACAACAACAACAACAACAACAACAACAACAACAACAACAAATCCTTCAACAAATTCTGCAACAACAAC
TGATTCCATGYAGGGATGTCGTCTTGCAACAACCCAACATAGCACATGCAAGCTCACAAGTATYGCAACAAAGTTACCAACTGTTG
CAACAATTATGTTGTCAGCAACTGTGGCAGACCCCCGAGCAGTCACGGTGCCAAGCCATCCACAATGTCATTCATGCTATTATTTT
GCATCAACAACAACAACAACAACAACAACAACAACAACAACAACAACAACAACAACAACCGTCGAGCCAGGTCTCCTACCAGCAGC
CTCAGCAACAATATCCATCAGGCCAGGGCTCGTTCCAGCCATCTCAGCAAAACCCACAGGCCCAGGGCTTTGTCCAACCTCAGCAA
CTGCCGCAGTTCGAGGAAATAAGGAACCTAGCGCTACAGACGCTACCAGCAATGTGCAATGTCTACATCCCTCCATATTGCTCGAC
CACCATTGCGCCATTTGGCATCATGAGTACTAACTGA

α-B12 amino acid

MKTFLILALLAIVATTTTTAVRVPVP.LQPQNPSQQQPQEQVPLVQQQQFLGQQQQQFPGQQQPFPPQQPYPQPQPFLPQLPYPQP
QPFPPQQSYPQPQPQYPQPQQPISQQQAQLQQQQQQQQQQQQQQQQQQILQQILQQQLIPCRDVVLQQPNIAHASSQVXQQSYQLL
QQLCCQQLWQTPEQSRCQAIHNVIHAIILHQQQQQQQQQQQQQQQQQQQPSSQVSYQQPQQQYPSGQGSFQPSQQNPQAQGFVQPQQ
LPQFEEIRNLALQTLPAMCNVYIPPYCSTTIAPFGIMSTN.

α-B13 nucleotide 882 bp

ATGAAGACCTTTCTCATCCTTGCCCTCCTTGCTATCGTGGCGACCACCACCACAACTGCAGTTAGAGTTCCAGTGCCATAATTGCA
GCCACAAAATCCATCTCAGCAACAACCACAAGAGCAAGTTCCATTGGTGCAACAACAACAATTTCTAGGGCAGCAACAACAACAAT
TTCCAGGGCAACAACAACCATTTCCACCACAACAGCCATATCCGCAGCCGCAACCATTTCTGCCACAACTACCATATCCGCAGCCG
CAACCATTTCCACCACAACAATCATATCCACAACCACAACCACAATATCCGCAACCACAACAACCAATTTCGCAGCAACAAGCACA
ACTACAACAACAACAACAACAACAACAACAACAACAACAACAAATCCTTCAACAAATTCTGCAACAACAACTGATTCCATGTAGGG
ATGTCGTCTTGCAACAACCCAACATAGCACATGCAAGCTCACAAGTATCGCAACAAAGTTACCAACTGTTGCAACAATTATGTTGT
CAGCAACTGTGGCAGACCCCCGAGCAGTCACGGTGCCAAGCCATCCACAATGTCATTCATGCTATTATTTTGCATCAACAACAACA
ACAACAACAACAACAACAACAACAACAACAACAACAACAACCGTCGAGCCAGGTCTCCTACCAGCAGCCTCAGCAACAATATC
CATCAGGCCAGGGCTCGTTCCAGCCATCTCAGCAAAACCCACAGGCCCAGGGCTTTGTCCAACCTCAGCAACTGCCGCAGTTCGAG
GAAATAAGGAACCTAGCGCTACAGACGCTACCAGCAATGTGCAATGTCTACATCCCTCCATATTGCTCGACCACCATTGCGCCATT
TGGCATCATGAGTACTAACTGA

α-B13 amino acid 294 aa

MKTFLILALLAIVATTTTTAVRVPVP.LQPQNPSQQQPQEQVPLVQQQQFLGQQQQQFPGQQQPFPPQQPYPQPQPFLPQLPYPQP
QPFPPQQSYPQPQPQYPQPQQPISQQQAQLQQQQQQQQQQQQQILQQILQQQLIPCRDVVLQQPNIAHASSQVSQQSYQLLQQLCC
QQLWQTPEQSRCQAIHNVIHAIILHQQQQQQQQQQQQQQQQQQPSSQVSYQQPQQQYPSGQGSFQPSQQNPQAQGFVQPQQLPQFE
EIRNLALQTLPAMCNVYIPPYCSTTIAPFGIMSTN.

α-B14 nucleotide 876 bp

ATGAAGACCTTTCTCATCCTTGCCCTCCTTGCTATCGTGGCGACCACCACCACAACTGCAGTTAGAGTTCCAGTGCCACAATTGCA
GCCACAAAATCCATCTCAGCAACAACCACAAGAGCAAGTTCCATTGGTGCAACAACAACAATTTCTAGGGCAGCAACAACAACAAT
TTCCAGGGCAACAACAACCATTTCCACCACAACAGCCATATCCGCAGCCGCAACCATTTCTGCCACAACTACCATATCCGCAGCCG
CAACCATTTCCACCACAACAATCATATCCACAACCACAACCACAATATCCGCAACCACAACAACCAATTTCGCAGCAACAAGCACA
ACTACAACAACAACAACAACAACAACAACAACAACAACAACAAATCCTTCAACAAATTCTGCAACAACAACTGATTCCATGCAGGG
ATGTCGTCTTGCAACAACCCAATATAGCACATGCAAGCTCAAAAGTATCGCAACAAAGTTACCAACTGTTGCAACAATTATGTTGT
CAGCAACTGTGGCAGACCCCCGAGCAGTCACGGTGCCAAGCCATCCACAATGTCATTCATGCTATTATTTTGCATCAACAACAACA
ACAACAACAACAACAACAACAACAACAACAACAACAACCGTCGAGCCAGGTCTCCTACCAGCAGCCTCAGCAACAATATCCATCAG
GCCAGGGCTTCTTCCAGCCATCTCAGCAAAACCCACAGGCCCAGGGCTTTGTCCAACCTCAGCAACTGCCGCAGTTCGAGGAAATA
AGGAACCTAGCGCTGCAGACGCTACCAGCAATGTGCAATGTCTACATCCCTCCATATTGCTCGACCACCATTGCGCCATTTGGCAT
CATGAGTACTAACTGA

α-B14 amino acid 292 aa

MKTFLILALLAIVATTTTTAVRVPVPQLQPQNPSQQQPQEQVPLVQQQQFLGQQQQQFPGQQQPFPPQQPYPQPQPFLPQLPYPQP
QPFPPQQSYPQPQPQYPQPQQPISQQQAQLQQQQQQQQQQQQQILQQILQQQLIPCRDVVLQQPNIAHASSKVSQQSYQLLQQLCC
QQLWQTPEQSRCQAIHNVIHAIILHQQQQQQQQQQQQQQQQQPSSQVSYQQPQQQYPSGQGFFQPSQQNPQAQGFVQPQQLPQFEEI
RNLALQTLPAMCNVYIPPYCSTTIAPFGIMSTN.

α-B15 nucleotide 873 bp

ATGAAGACCTTTCTCATCCTTGCCCTCCTTGCTATCATGGCGACCACCACCACAACTGCAGTTAGAGTTCCAGTGCCACAATTGCA
GCCACAAAATCCATCTCAGCAACAACCACAAGAGCAAGTTCCATTGGTGCAACAACAACAATTTCTAGGGCAGCAACAACAACAAT
TTCCAGGGCAACAACAACCATTTCCACCACAACAGCCATATCCGCAGCCGCAACCATTTCTGCCACAACTACCATATCCGCAGCCG
CAACCATTTCCACCACAACAATCATATCCACAACCACAACCACAATATCCGCAACCACAACAACCAATTTCGCAGCAACAAGCACA
ACTACAACAACAACAACAACAACAACAACAACAACAAATCCTTCAACAAATTCTGCAACAACAACTGATTCCATGCAGGGATGTCG
TCTTGCAACAACCCAATATAGCACATGCAAGCTCACAAGTATCGCAACAAAGTTACCAACTGTTGCAACAATTATGTTGTCAGCAA
CTGTGGCAGACCCCCGAGCAGTCACGGTGCCAAGCCATCCACAATGTCATTCATGCTATTATTTTGCATCAACAACAACAACAACA
ACAACAACAACAACAACAACAACAACAACAACAACCGTCGAGCCAGGTCTCCTACCAGCAGCCTCAGCAACAATATCCATCAGGCC
AGGGCTTCTTCCAGCCATCTCAGCAAAACCCACAGGCCCAGGGCTTTGTCCAACCTCAGCAACTGCCGCAGTTCCAGGAAATAAGG
AACCTAGCGCTGCAGACGCTACCAGCAATGTGCAATGTCTACATCCCTCCATATTGCTCGACCACCATTGCGCCATTTGGCATCAT
GAGTACTAACTGA

α-B15 amino acid 291 aa

MKTFLILALLAIMATTTTTAVRVPVPQLQPQNPSQQQPQEQVPLVQQQQFLGQQQQQFPGQQQPFPPQQPYPQPQPFLPQLPYPQP
QPFPPQQSYPQPQPQYPQPQQPISQQQAQLQQQQQQQQQQQILQQILQQQLIPCRDVVLQQPNIAHASSQVSQQSYQLLQQLCCQQ
LWQTPEQSRCQAIHNVIHAIILHQQQQQQQQQQQQQQQQQPSSQVSYQQPQQQYPSGQGFFQPSQQNPQAQGFVQPQQLPQFQEIR
NLALQTLPAMCNVYIPPYCSTTIAPFGIMSTN.

α-B16 nucleotide 891 bp

ATGAAGACCTTTCTCATCCTTGCCCTCCTTGCTATCGTGGCGACCACCACCACAACTGCAGTTAGAGTTCCAGTGCCACAATTGCA
GCCACAAAATCCATCTCAGCAACAACCACAAGAGCAAGTTCCATTGGTGCAACAACAACAATTTCTAGGGCAGCAACAACAACAAT
TTCCAGGGCAACAACAACCATTTCCACCACAACAGCCATATCCGCAGCCGCAACCATTTCTGCCACAACTACCATATCCGCAGCCA
CAACCATTTCCACCACAACAATCATATCCACAACCACAACCACAATATCCGCAACCACAACAACCAATTTCGCAGCAACAAGCACA
ACTACAACAACAACAACAACAACAACAACAACAACAAATCCTTCAACAAATTCTGCAACAACAACTGATTCCATGCAGGGATGTCG
TCTTGCAACAACCCAATATAGCACATGCAAGCTCACAAGTATCGCAACAAAGTTACCAACTGTTGCAACAATTATGTTGTCAGCAA
CTGTGGCAGACCCCCGAGCAGTCACGGTGCCAAGCCATCCACAATGTCATTCATGCTATTATTTTGCATCAACAACAACAACAACA
ACAACAACAACAACAACAACAACAACAACAACAACAACAACAACAACAACAACCGTCGAGCCAGGTCTCCTACCAGCAGCCTCAGC
AACAATATCCATCAGGCCAGGGCTTCTTCCAGCCATCTCAGCAAAACCCACAGGCCCAGGGCTTTGTCCAACCTCAGCAACTGCCG
CAGTTCGAGGAAATAAGGAACCTAGCGCTGCAGACGCTACCAGCAATGTGCAATGTCTACATCCCTCCATATTGCTCGACCACCAT
TGCGCCATTTGGCATCATGAGTACTAACTGA

α-B16 amino acid 297 aa

MKTFLILALLAIVATTTTTAVRVPVPQLQPQNPSQQQPQEQVPLVQQQQFLGQQQQQFPGQQQPFPPQQPYPQPQPFLPQLPYPQP
QPFPPQQSYPQPQPQYPQPQQPISQQQAQLQQQQQQQQQQQILQQILQQQLIPCRDVVLQQPNIAHASSQVSQQSYQLLQQLCCQQ
LWQTPEQSRCQAIHNVIHAIILHQQQQQQQQQQQQQQQQQQQQQQQPSSQVSYQQPQQQYPSGQGFFQPSQQNPQAQGFVQPQQLP
QFEEIRNLALQTLPAMCNVYIPPYCSTTIAPFGIMSTN.

α-B17 nucleotide 891 bp

ATGAAGACCTTTCTCATCCTTGCCCTCCTTGCTATCGTGGCGACCACCACCACAACTGCAGTTAGAGTTCCAGTGCCACAATTGCA
GCCACAAAATCCATCTCAGCAACAACCACAAGAGCAAGTTCCATTGGTGCAACAACAACAATTTCTAGGGCAGCAACAACAACAAT
TTCCAGGGCAACAACAACCATTTCCACCACAACAGCCATATCCGCAGCCGCAACCATTTCTGCCACAACTACCATATCCGCAGCCG
CAACCATTTCCACCACAACAATCATATCCACAACCACAACCACAATATCCGCAACCACAACAACCAATTTCGCAGCAACAAGCACA
ACTACTACAACAACAACAACAACAACAACAACAACAACAACAACAACAACAACAAATCCTTCAACAAATTCTGCAACAACAACTGATTC
CATGCAGGGATGTCGTCTTGCAACAACCCAATATAGCACATGCAAGCTCACAAGTATCGCAACAAAGTTACCAACTGTTGCAACAA
TTATGTTGTCAGCAACTGTGGCAGACCCCCGAGCAGTCACGGTGCCAAGCCATCCACAATGTCATTCATGCTATTATTTTGCATCA
ACAACAACAACAACAACAACAACAACAACAACAACAACAACAACAACAACAACAACCGTCGAGCCAGGTCTCCTACCAGCAGCCTCAGC
AACAATATCCATCAGGCCAGGGCTTCTTCCAGCCATCTCAGCAAAACCCACAGGCCCAGGGCTTTGTCCAACCTCAGCAACTGCCG
CAGTTCGAGGAAATAAGGAACCTAGCGCTGCAGACGCTACCAGCAATGTGCAATGTCTACATCCCTCCATATTGCTCGACCACCAT
TGCGCCATTTGGCATCATGAGTACTAACTGA

α-B17 amino acid 297 aa

MKTFLILALLAIVATTTTTAVRVPVPQLQPQNPSQQQPQEQVPLVQQQQFLGQQQQQFPGQQQPFPPQQPYPQPQPFLPQLPYPQP
QPFPPQQSYPQPQPQYPQPQQPISQQQAQLLQQQQQQQQQQQQQQQILQQILQQQLIPCRDVVLQQPNIAHASSQVSQQSYQLLQQ
LCCQQLWQTPEQSRCQAIHNVIHAIILHQQQQQQQQQQQQQQQQQQPSSQVSYQQPQQQYPSGQGFFQPSQQNPQAQGFVQPQQLP
QFEEIRNLALQTLPAMCNVYIPPYCSTTIAPFGIMSTN.

α-B18 nucleotide 885 bp

ATGAAGACCTTTCTCATCCTTGCCCTCCTTGCTATCGTGGCGACCACCACCACAACTGCAGTTAGAGTTCCAGTGCCACAATTGCA
GCCACAAAATCCATCTCAGCAACAACCACAAGAGCAAGTTCCATTGGTGCAACAACAACAATTTCTAGGGCAGCAACAACAACAAT
TTCCAGGGCAACAACAACCATTTCCACCACAACAGCCATATCCGCAGCCGCAACCATTTCTGCCACAACTACCATATCCGCAGCCG
CAACCATTTCCACCACAACAATCATATCCACAACCACAACCACAATATCCGCAACCACAACAACCAATTTCGCAGCAACAAGCACA
ACTACTACAACAACAACAACAACAACAACAACAACAACAACAACAACAACAACAAATCCTTCAACAAATTCTGCAACAACAACTGATTC
CATGCAGGGATGTCGTCTTGCAACAACCCAATATAGCACATGCAAGCTCACAAGTATCGCAACAAAGTTACCAACTGTTGCAACAA
TTATGTTGTCAGCAACTGTGGCAGACCCCCGAGCAGTCACGGTGCCAAGCCATCCACAATGTCATTCATGCTATTATTTTGCATCA
TCGTCATCAACAACAACAACAACAACAACAACAACAACAACAACAACAACCGTCGAGCCAGGTCTCCTACCAGCAGCCTCAGCAACAAT
ATCCATCAGGCCAGGGCTTCTTCCAGCCATCTCAGCAAAACCCACAGGCCCAGGGCTTTGTCCAACCTCAGCAACTGCCGCAGTTC
GAGGAAATAAGGAACCTAGCGCTGCAGACGCTACCAGCAATGTGCAATGTCTACATCCCTCCATATTGCTCGACCACCATTGCGCC
ATTTGGCATCATGAGTACTAACTGA

α-B18 amino acid 295 aa

MKTFLILALLAIVATTTTTAVRVPVPQLQPQNPSQQQPQEQVPLVQQQQFLGQQQQQFPGQQQPFPPQQPYPQPQPFLPQLPYPQP
QPFPPQQSYPQPQPQYPQPQQPISQQQAQLLQQQQQQQQQQQQQQQILQQILQQQLIPCRDVVLQQPNIAHASSQVSQQSYQLLQQ
LCCQQLWQTPEQSRCQAIHNVIHAIILHHRHQQQQQQQQQQQQQPSSQVSYQQPQQQYPSGQGFFQPSQQNPQAQGFVQPQQLPQF
EEIRNLALQTLPAMCNVYIPPYCSTTIAPFGIMSTN.

α-B19 nucleotide 744 bp

ATGAAGACCTTTCTCATCCTTGCCCTCCTTGCTATCGTGGCGATGCGACCACCGCCACAACTGCAGTTAGAGTTCCAGTGCCACAA
TTGCAGCCACAAAATCCATCTCAGCAACAACCACAAGAGCAAGTTCCATTGGTGCAACAACAACAATTTCTAGGGCAGCAATAACC
ATTTCGACCACAACAACCATATCCACAGCCGCAACCATTTCCTCCACAACTACCATGTCCGCAGCCGCAACCATTTCCACCACAAC
AATCATATCCACAACCACAACCACAGTATCCGCAACCACAACAAACAATTTCGCAGCAACAAGCACAACAACAACAATGACAACAA
CAACAACAAATCCTTCAACAAATTCTACAACAACAACTGATTCCATGCAGGGATGTCGTGTTGCAACAACCCCGAGCTGTCACGGT
GCCAAGTCATCCACAATGTCGTTCATGCTATTATTCTCCATCAACAACAACAACAAAAACAACAACAACAACAACAACCGTCGAGC
CAGGTCTTCTACCAGCAGCCTCAGCAACAATATCCATCAGGCCAGGGTTCCTTCCAGCCATCTCAGCAAAACCCACAGGCCCATGG
CTTTGTCCAACCTCAGCAACTGCCACAGTTTGAGGAAATAAGGAACCTAGCGCTACAGACACTACCAGCAATGTGCAATGTCTACA
TCCCTCCATATTGCTCGACCACCATTGCGCCATTTGGCATCGTGAGTACTAACTGA

α-B19 amino acid 298 aa

MKTFLILALLAIVAMRPPPQLQL...VPVPQLQPQNPSQQQPQEQVPLVQQQQFLGQQ.PFRPQQPYPQPQPFPPQLPCPQPQPFP
PQQSYPQPQPQYPQPQQTISQQQAQQQQ.QQQQQILQQILQQQLIPCRDVVLQQPRAVTVPSHPQCRSCYYS...QQQQQKQQQQQ
QPSSQVFYQQPQQQYPSGQGSFQPSQQNPQAHGFVQPQQLPQFEEIRNLALQTLPAMCNVYIPPYCSTTIAPFGIVSTN.

α-B20 nucleotide 860 bp

ATGAAGACCTTTCTCATCCTTGCCCTCCTTGCTATCATGGCAATGCGACCACCGCCACAACTGCAGTTAGAGTTCCAGTGCCACAA
TTGCAACCAGAAAATCCATCTCAGCAACAACCACAAGAGCAAGTTCCATTGGTGCAACAACAACAATTTCTAGGGCAGCAACAACA
ACAATTTCCAGGGCAGCAATAACCATTTCGACCACAACAGCCATATCCGCAGCCGCAACCATTTCCTCCACAACTACCATGTCCGC
AACCGCAACCATTTCCACCACAACAATCATATCCACAACCACAACCACAGTATCCGCAACCACAACAAACAATTTCGCAGCAACAA
GCACAACAACAACAATGACAACCACAACAACAAATCCTTCAACAAATTCTACAAAAACAACTGATTCCATGCAGGGATGTCGTCTT
GCAACAACCCAACATAGCACATGCAAGCTCACAAGTATCACAACAAAGTTACCATCTGTTGCAACAATTATGTTGTTAGCAACTGT
GGCAGACCCCCGAGCTGTCACGGTGCCAAGTCATCCACAATGTCGTTCATGCTATTATTCTCCATCAACAACAACAACAAAAACAA
CAACAACAACAACAACAACAACCGTCGAGCCAGGTCTTCTAGCAGCAACCTCAGCAACAATATCCATCAGCCCAGGGCTCCTTCCA
GCCATCTGAGCAAAACCCACAGGCCGATGGCTTTGTCCAACCTCAGCAACTGCCGCAGTTTGAGTAAATAAGGAACCTAGCGCTGC
AGACACTACCAGCAATGTGCAATGTCTACATCCCTCCATATTGCTCGACCACCATTGCGCCATTTGGCATCGTGAGTACTAATTGA

α-B20 amino acid 295 aa

MKTFLILALLAIMAMRPPPQLQLEFQCHNC...PVPQLQPENPSQQQPQEQVPLVQQQQFLGQQQQQFPGQQ.PFRPQQPYPQPQP
FPPQLPCPQPQPFPPQQSYPQPQPQYPQPQQTISQQQAQQQQ.QPQQQILQQILQKQLIPCRDVVLQQPNIAHASSQVSQQSYHLL
QQLCC.QLWQTPELSRCQVIHNVVHAIILHQQQQQKQQQQQQQQPSSQVF.QQPQQQYPSAQGSFQPSEQNPQADGFVQPQQLPQF
E.IRNLALQTLPAMCNVYIPPYCSTTIAPFGIVSTN.

α-B21 nucleotide 834 bp

ATGAACCTTCTCCTTGCCCTCGCCCTCCTTGCTATCGTGGCGACCACCGCCACAACTGCAGTTAGAGTTCCAGTGCCACAATTGCA
GCCACAAAATCCATCTCAGCAACAACCACAAGAGCAAGTTCTATTGGTGCAACAACAACCATTTCTAGGGCAGCAACAACAACAAT
TTCCAGGGCAGCAATAACCATTTCGACCACAACAGCCATATCCGCAGCCGCAACTATTTCCTCCAAAACTACCATATCCGCAGCCG
CAACCATTTACACCACATCAATCATATCCACAACCACAACCACAGTATCCGCAACCACAACAAGCAATTTCGCAGCAACAAGGACA
ACAACAACAACAACAACAAATCCTTCAACAAATTCTGCAACAACAACTGATTCCATGCAGGGATGTCGTCTTGCAACAACCCAACA
TAGCACATGCAAGCTCACAAGTATCTCAACAAAGTTACCATCTATTGCAACAATTATGTTGTCAGCAACTGTGGCAGACCCCCGAG
CTGTCACAGTGCCAAGTCATCCACAATGTCGTTCATGCTATTATTCTGCATCATCAATAACAACAAAAACAACAACAACAACCGTC
GAGCCAGGTCTTCTACCAGCAGCCTCAGCAACAATATCCATCAGGCCACGGCTCCTTCCAGCCATCTCAGCAAAACCCACAGACCC
AGGGCTTTGTCCAACCTCAGCAACTGCTGCAGTTTGAGGAAATAAGGAACGTAGCGCTGCAGACACTACCAGCAATGTGCAATGTC
TACATCCCTCCATATTGCTCGACCACCATTGCGCCATTTGGCATCGTGAGTACTAACTGA

α-B21 amino acid 278 aa

MNLLLALALLAIVATTATTAVRVPVPQLQPQNPSQQQPQEQVLLVQQQPFLGQQQQQFPGQQ.PFRPQQPYPQPQLFPPKLPYPQP
QPFTPHQSYPQPQPQYPQPQQAISQQQGQQQQQQILQQILQQQLIPCRDVVLQQPNIAHASSQVSQQSYHLLQQLCCQQLWQTPE
LSQCQVIHNVVHAIILHHQ.QQKQQQQPSSQVFYQQPQQQYPSGHGSFQPSQQNPQTQGFVQPQQLLQFEEIRNVALQTLPAMCNV
YIPPYCSTTIAPFGIVSTN.

α-B22 nucleotide 2087 bp

ATGAAGACCTTTCTCATCCTTGCCCTCCTTGCTATCGCGGTGACCACCGCCACTACTGCAGTGAGAGTTCCAGTGCCATAATTGCA
GCCACAAAATCCATCTCAGCAACAACCACAAGAGCAAGTTCCATTGGTGCAACAACAACAATTTCTAGGGCAGCAACAACAACACT
TTCCAGGGCAGCAACAACCATTTCCACCACAACAGCCATATCCGCAGCCGCAACCATTTCCACCACAACTACCATATCTGCAGCCG
CAACCATTTCCACCACAACAATCATATCCACAACCACAACCACAGTATCCGCAATCACAACAACAACCAATTTCACAGCAACAACA
ACAACAACAACAACAACAACAACAACAACAACAACAACAACAAATCCTTCAACAAATTCTGCAACAACAATTGATTCCATGCA
GGGATGTCATCTTGCAACAACCCAACATAGCACATGCAAGCTCACAAGTATCGCAACAAAGTTACCAACTTTTGCAACAATTATGT
TGTCAGCAACTGTGGCAGACCCCTGAGCAGTCACGGTGCCAAGCCATCCACAATGTCGTTCATGCTATTATTCTGCATCATCATCA
TCATCATCATCAACAACAACAACAACAACAACAACAACAACAACAACAACAACAACAACAACAACAACAACAACACCAAC
AACAACAACAGCAACAACAACAACAGCAACAACAACAGCAGCAACAACAACAGCAGCAACAACAACAGCAGCAACAACAACAGCAA
CAACAACAGCAACAACAACAGCAACAACAACAGCAACAACAACAGCAGCAACAACAGCAGCAGCAGCAACAACAGCAGCAACAACA
GCAGCAACAACAACAACAACAACAACAACAACAACAACAACAACAACAACAACAACAACAACAACAACAACAACAACAACAACAAC
AACAACAACAACAACAACAACAACAACAACAACAACAACAACAACAACAACAACAACAACAACAACAACAACAACAACAACAACAA
CAACAACAACAACAACAACAACAACAACAACAACAACAACAACAACAACAACAACAACAACAACAACAACAACAACAACAACAACA
ACAACAACAACAACGACGACAACAACAACAACGACAACAACAACAACGACAACAACAACAACGACCACAACAACAACAACGACAAC

AACAACAACGACAACAACGACAACAACAACGACGACAACAACAACGACGACAACAACAACGACGACAACAACGACGACGACAACAA
CGACGACGACAACAACGACGACGACAACAACAACGACGACAACAACAACGACGACAACAACAACGACGACAACAACAACGACGACA
ACAACAACGACGACAACAACAACGACGACAACAACGACGACGACGACAACGACGACGACGACGACAACGACGACGACAACGACGAC
GACAACAACGACGACAACGACAACAACGACGACGACAACAACGACAACAACGACGACGACAACGACGACGACGACGACAACAACGA
CGACGACAACAACGACGACGACAACAACGACGACAACAACAGCGACGACGACAACAACGACGACGACAACAACGACGACGACAACA
ACGACGACGACAACAACGACGACGACAACAACGACGACGACAACAACGACGACGACAACAACGACGACAACAACGACAACGACAAC
AACGACAACAACAACAACGACAACAACAACAACGACAACAACAACAACAACAACGACAACAACAACAACAATAACAACAACAACAA
CAATAACAACAACAACAACAACAACAACAACAACAACAACAACAACGGTCGAGCGATGTCTCCTACCAGCAGCCTCAGCAAAAATA
TCCATCAGGCCAGGGCTCCTTCCAGCCATCTCAGCAAAACCCACAGGCCCAGGGCTTTGTCCAACCTCAGCAACTGCCCCAATTCG
AGAAAATAAGGAACCTAGCATTGCAGACGCTACCAGCAATGTGCAATGTCTACATCCCTCCATATTCTCGACCACCATTGCGCCAT
TTGGCATCGTCGGCACTAACTGA

α-B22 amino acid 695 aa

MKTFLILALLAIAVTTATTAVRVPVP.LQPQNPSQQQPQEQVPLVQQQQFLGQQQQHFPGQQQPFPPQQPYPQPQPFPPQLPYLQP
QPFPPQQSYPQPQPQYPQSQQQPISQQQQQQQQQQQQQQQQQQILQQILQQQLIPCRDVILQQPNIAHASSQVSQQSYQLLQQLC
CQQLWQTPEQSRCQAIHNVVHAIILHHHHHHHQQQQQQQQQQQQQQQQQQQQQQQHQQQQQQQQQQQQQQQQQQQQQQQQQQQQQ
QQQQQQQQQQQQQQQQQQQQQQQQQQQQQQQQQQQQQQQQQQQQQQQQQQQQQQQQQQQQQQQQQQQQQQQQQQQQQQQQQQQQQ
QQQQQQQQQQQQQQQQQQQQQQQQQQQQQQQQQRRQQQRQQQQRQQQQRPQQQQRQQQQRQQRQQQRRQQQRRQQQRRQQRRRQQ
RRRQQRRRQQQRRQQQRRQQQRRQQQRRQQQRRQQQRRQQRRRRQRRRRRQRRRQRRRQQRRQRQQRRRQQRQQRRRQRRRRRQQR
RRQQRRRQQRRQQQRRRQQRRRQQRRRQQRRRQQRRRQQRRRQQRRRQQRRRQQRRQQRQRQQRQQQQRQQQQRQQQQQQRQQQQQ.QQQQ
Q.QQQQQQQQQQQQQRSSDVSYQQPQQKYPSG.GLLPAISAKPTGPGLCPTSATAPIRENKEPSIADATSNVQCLHPSIFSTTIAP
FGIVGTN

α-B23 nucleotide 879 bp

ATGAAGTCATTTCTCATCCTTGCCCTCCTTGCTATCGTGGCGACCACCGCCACAACTGCAGTTAGAGTTCCAGTGCCACAATTGCA
GCCACAAAATCCATCTCAGCAACAACCACAAGAGCAAGTTCCATTGGTGCAACAACAACAATTTCTAGGGCAGCAACAACAAAAAT
TTCCAGGGCAGCAGCAACCATTTCCACCACAACAGCCATATCCGCAGCCGCAACCATTTCTGCCACAACTACCATTTCCGCAGCCG
CAACCATTTCCACCACAACAATCATATCCACAACCGCAACCACAGTATCCACAACCACAACAACCAATTTCGCAGCAACAAGCACA
ACAACAACAACAACAACAACAACAAATCCTTCAACAAATTCTGCAACAACAACTGATTCCATGCAAGGATGTCGTCTTGCAACAAC
CCAACATAGCACATGCTAGCTCACAAGTATCGCAACAAAGTTACCAACTGTTGCAACAATTATGTTGTCAGCAATTGTGGCAGACC
CCCGAACAGTCACGGTGCCAAGCCATCCACAATGTCGTGCATGCTATTATTCTGCATCATCAACAACAACAACAACAACAACAACA
ACAACAACAACAACAACAACAACAACAACAACAACAACAACCGTCGAGCCAGGTCTCCTACCAGCAGCCTCAACAATAATATCCAT
CGGCCCAGGGCTCCTTCCAGCCATCTCAGCAAAACCCACAGGCCCAGGGCTTTGTCCAACCTCAACAACTGCCCCAGTTCGAGGAA
ATAAGGAACCTAGCACTGCAGACGCTACCTGCAATGTGCAATGTCTACATCCCTCCATATTGCTCGACCACCATTGTGCCATTTGG
CATCATCGGTACTAACTGA

α-B23 amino acid 293 aa

MKSFLILALLAIVATTATTAVRVPVPQLQPQNPSQQQPQEQVPLVQQQQFLGQQQQKFPGQQQPFPPQQPYPQPQPFLPQLPFPQP
QPFPPQQSYPQPQPQYPQPQQPISQQQAQQQQQQQQILQQILQQQLIPCKDVVLQQPNIAHASSQVSQQSYQLLQQLCCQQLWQT
PEQSRCQAIHNVVHAIILHHQQQQQQQQQQQQQQQQQQQQQPSSQVSYQQPQQ.YPSAQGSFQPSQQNPQAQGFVQPQQLPQFEE
IRNLALQTLPAMCNVYIPPYCSTTIVPFGIIGTN.

α-B24 nucleotide 231 bp

AGCCAGTTCTCCTACCAGCAGCCCCATAACCAATATCCATCAGGCCAAGGCTCCTTCCAGCCATCTCAGCAAAACCCACAGGCCTA
GGGCTCTGTCCAGCCTCAACAACTGCCCCAGTTCGAGGAAATAAGGAACCTAGCGCTGCAGACTCTACCAACAATGTGCAATGTCT
ACATCCCTCCATATTGCTCGGCCACCATTGCGCCATTTGGCATCGTCGGTAGTAACTGA

α-B24 amino acid 77 aa

SQFSYQQPHNQYPSGQGSFQPSQQNPQA.GSVQPQQLPQFEEIRNLALQTLPTMCNVYIPPYCSATIAPFGIVGSN.

α-B25 nucleotide 933 bp

ATGAAGACCTTTATCATCCTTGCCCTCCTTGCTATTGTGGCGACCACCGCCACAACTGCAGTTACAGTTCCAGTGCCACAATTGCA
GCCACAAAATCCATCTCAGCAACAACCACAAAAGCAAGTTCCATTGGTGCAACAACAACAATTTCTAGGGCAGCAACAACCATTTC
CACCACAACAGCCATATCCGCAGCCGCAACCATTTCCATCACAACAACCATATCCACAGCCACAACCATTTCTGCCACAACTACCA
TATCCGCGTCCGCAACCATTTCTACCACAACAACCATATCCACAACCGGAACCACAGTATCCGCAACCACAACAACCAATTTCGCA
ACAGCAGCAGCAGCAACAACAACAACAACAACAAATCCTTCAACAAATTTTGCAACAACAACTGATTCCATGCAGGGATGTCATCT

TGCAACAACACAACATAGCGCATGCAAGCTCACAAGTTTTGCAACAAAGTACTTACCAGTTGTTGCAACAATTGTGTTGTCAGCAG
CTGTGGCAGATCCCCGAGCAGTCGCGGTGCCAAGCCATCCAGAATGTTGTTCATGCTATTATTCTGCATCAACAACAACAACGACA
ACAACAACAACAACAACAACAACAACAACAACAACAACAACAACAACAACAACAACAACAACAACAACAACAACAACAACAACAGC
AGCAACAACCGTCGAGCCAGGTCTCCTTCCAACAGCCTCAGCAACAATATCCATCAGGCCAGGGCTCCTTCCAGCCATCTCAGCAA
AACCCACAGGCCCAGAGCTCTGTCCAACATCAACAACTACCCCAATTTGAGGAAATAAGGAACCTAGCACTGCAGACGCTACCAGC
AGTGTGCAATGTCTACATCCCTCCATATTGCTCGACCACCATTGCGCCATTTGGCATCTTCGGTACTAACTGA

α-B25 amino acid 311 aa

MKTFIILALLAIVATTATTAVTVPVPQLQPQNPSQQQPQKQVPLVQQQQFLGQQQPFPPQQPYPQPQPFPSQQPYPQPQPFLPQLP
YPRPQPFLPQQPYPQPEPQYPQPQQPISQQQQQQQQQQQQQILQQILQQQLIPCRDVILQQHNIAHASSQVLQQSTYQLLQQLCCQQ
LWQIPEQSRCQAIQNVVHAIILHQQQQRQQQQQQQQQQQQQQQQQQQQQQQQQQQQQQQQPSSQVSFQQPQQQYPSGQGSFQPSQQ
NPQAQSSVQHQQLPQFEEIRNLALQTLPAVCNVYIPPYCSTTIAPFGIFGTN.

α-B26 nucleotide 852 bp

ATGAAGACCTTTCTCATCCTTGCCCTCCTTGCTATCGCGGCGACCACCGCCACAACTGCAGTTAGAGTTCCAGTGCCACAATTGTA
GCCGCAAAATCCATCTCAACAACGACCACAATAGTAATTTCCGTTGGTGCAACAACAACAATTTCCAGGGCAGCAACAACCATTCC
CACCACAACAGCCATATCCGCAGCCGCAACCGTTTCTGTCACAACAACCATATTCGCAACCGTAACCATTTCCGCCACAACTACCA
TATCCACAAGCGCAACCATTTCCAACACAACAACCATATCCACAACCGCAACCACAGTATCCGCAACCACAACAACCAATTTCGCA
GCAGCAGCAGCAGCAACAACAACAACAACAACAACAACAACAACAAATCCTTCAACAAATTCTGCAACAACAAATGATTCCATGCA
GGGATGTCGTCTTGCAACAACACAACATTGCATATGCAAGCTCACAAGTATTGCAACAAAGTAGTTACCAACTGTTGCAACAATTA
TGTTGTCAGCAGCTGTGGCAGATCCCCGAGCAGTCGCGGCGCCAAGCCATCCACAGTGTCGTTCATGCTATTATTTTCCATCATCA
TCAACAACAACAACCGTCGAGCCAGGTCTCCTACCAGCAGCCTCAACAACAATATCCATCAAGCCAGGGCTCCTTCTAGCCATCTC
AGCTAAACCCACAGGCCCAGGGCTCTGTCCAGCCTCAACAACTGCCCCGGTTCAAGGAAATAAGTAACCTAGCGCTGCAGATGCTG
CCAGCAATGTGCAATGTCTACATCCCTCCATATTGCTTGAGCACCATTGTGCCATTTGGCATCTTCGGTACTAACTAA

α-B26 amino acid 284 aa

MKTFLILALLAIAATTATTAVRVPVPQL.PQNPSQQRPQ..FPLVQQQQFPGQQQPFPPQQPYPQPQPFLSQQPYSQP.PFPPQLP
YPQAQPFPTQQPYPQPQPQYPQPQQPISQQQQQQQQQQQQQQQQILQQILQQQMIPCRDVVLQQHNIAYASSQVLQQSSYQLLQQL
CCQQLWQIPEQSRRQAIHSVVHAIIFHHHQQQQPSSQVSYQQPQQQYPSSQGSF.PSQLNPQAQGSVQPQQLPRFKEISNLALQML
PAMCNVYIPPYCLSTIVPFGIFGTN.

α-D1 nucleotide 864 bp

ATGAAGACtTTTCTCATCCTTGCCCTCCTTGCTATtGTGGCGACCACTGCCACAACTGCAGTTAGAGTTCCAGTGCCACAATTGCA
GCCGCAAAATCCATCTCAGCAACAACCACAAGAGCAAGTTCCATTGGTGCAACAACAACAATTTCCAGGGCAGCAACAACCATTTC
CACCACAACAGCCATATCCGCAGCCGCAACCATTTCCATCACAACAACCATATCCGCAGCCGCAACCATTTCCGTCACAACTACCA
TATCCGCAGCCGCAACCATTTCCACCACAACAACCCTATCCACAACCGCAACCACAATATCCGCAACCACAACAACCAATTTCGCA
GCAACAAGCACAACAACAACAACAACAACAACAACAACAAATCCTTCAACAAATTcTGCAACAACAACTGATTCCATGCAGGGATG
TCGTCTTGCAACAACACAACATAGCGCATGCAAGCTCACAAGTATTGCAACAAAGTAGTTACCAACTGTTGCAACAATTATGTTGT
CAGCAGCTGTGGCAGATCCCCGAGCAGTCGCGGTGCCAAGCCATCCACAATGTCGTTCATGCTATTATTCTGCATCAACAACAACA
ACAACAACAACAACAACAACAACAACCATCGAGCCAGGTTTCCTACCAGCAGCCTCAGCAACAATATCTATCAGGCCAGGGCTCCT
TCCAGCCATCTCAGCAAAACCCACAGGCCCAGGGCTCTGTCCAGCCTCAACAACTGCCCCAGTTCGAGGAAATAAGGAACCTAGCG
CTACAGACGCTACCAGCAACCTGCAATGTCTACATCCCTCCATATTGCTCGACCACCATTGCGCCATTTGGCATCTTCGGTACTAA
CTGA

α-D1 amino acid 288 aa

MKTFLILALLAIVATTATTAVRVPVPQLQPQNPSQQQPQEQVPLVQQQQFPGQQQPFPPQQPYPQPQPFPSQQPYPQPQPFPSQLP
YPQPQPFPPQQPYPQPQPQYPQPQQPISQQQAQQQQQQQQQQILQQILQQQLIPCRDVVLQQHNIAHASSQVLQQSSYQLLQQLCC
QQLWQIPEQSRCQAIHNVVHAIILHQQQQQQQQQQQQPSSQVSYQQPQQQYLSGQGSFQPSQQNPQAQGSVQPQQLPQFEEIRNLA
LQTLPATCNVYIPPYCSTTIAPFGIFGTN.

α-D2 nucleotide 251 bp

AACAACAACAACAACAACAACAACCGTTGAGCCAGGTCTCCTTCCAGCAGCCTCAGCAACAATATCCATCAGGCCAGGGCTCCTTCCAG
CCATCTCAGCAAAACCCACAGGCCCAGGGCTCTTTCCAGCCTCAACAACTGCCCCAGTTTGAGGAAATAAGGAACCTAGCGCTACA
GACGCTACTTGCAATGTGCGATGTCTATATCCCTCCATATTGCACCATTGCTCCATTTGGCATCTTTGGTACTAACTGA

α-D2 amino acid 83 aa

QQQQQQPLSQVSFQQPQQQYPSGQGSFQPSQQNPQAQGSFQPQQLPQFEEIRNLALQTLLAMCDVYIPPYCTIAPFGIFGTN.

α-D4 nucleotide 876 bp

ATGAAGACCTTTCTCATCCTTGCCCTCCTTGCTATTGTGGCGACCACCGCCACAACTGCAGTTAGAGTTCCAGTGCCACAATTGCA
GCTACAAAATCCATCTCAGCAACAGCCACAAGAGCAAGTTCCATTGGTACAAGAACAACAATTTCCAGGGCAGCAACAACCATTTC
CACCACAACAGCCATATCCGCAGCCGCAACCATTTCCATCACAACAACCATATCTGCAGCTGCAACCATTTCCACAGCCGCAACTA
CCATATCCGCAGCCGCAACCATTTCGACCACAACAACCATATCCACAGCCGCAACCACAGTATTCGCAACCACAACAACCAATTTC
GCAGCAGCAGCAGCAGCAGCAGCAACAACAACAACAACAACAACAAATCCTACAACAAATTTTGCAACAACAACTGATTCCATGCA
GGGATGTTGTATTGCAACAACACAACATAGCGCATGGAAGCTCACAAGTTTTGCAAGAAAGTACTTACCAGCTGGTGCAACAATTG
TGTTGTCAGCAGCTGTGGCAGATCCCCGAGCAGTCGCGGTGCCAAGCCATCCACAATGTTGTTCATGCTATTATTCTGCATCAACA
ACACCACCACCACCAACAACAACAACAACAACAACAACAACAACCGTTGAGCCAAGTCTCCTTCCAACAGCCTCAGCAACAATATC
CATCAGGCCAGGGCTTCTTCCAACCATCTCAGCAAAACCCACAGGCCCAGGGCTCTTTCCAGCCTCAACAACTGCCCCAGTTTGAG
GCAATAAGGAACCTAGCGCTACAGACGCTACCTGCAATGTGCAATGTGTATATCCCTCCATATTGCACCATTGCTCCATTTGGCAT
CTTCGGTACTAACTGA

α-D4 amino acid 292 aa

MKTFLILALLAIVATTATTAVRVPVPQLQLQNPSQQQPQEQVPLVQEQQFPGQQQPFPPQQPYPQPQPFPSQQPYLQLQPFPQPQL
PYPQPQPFRPQQPYPQPQPQYSQPQQPISQQQQQQQQQQQQQQQILQQILQQQLIPCRDVVLQQHNIAHGSSQVLQESTYQLVQQL
CCQQLWQIPEQSRCQAIHNVVHAIILHQQHHHHQQQQQQQQQQPLSQVSFQQPQQQYPSGQGFFQPSQQNPQAQGSFQPQQLPQFE
AIRNLALQTLPAMCNVYIPPYCTIAPFGIFGTN.

α-D5 nucleotide 927 bp

ATGAAGACCTTTCTCATCCTTGCCCTCCTTGCTATTGTAGCAACCACCGCCACAATTGCAGTTAGAGTTCCAGTGCCACAATTGCA
GCCACAAAATCCATCTCAGCAACAACCACAAGAGCAAGTTCCATTGGTACAACAACAACAATTTCCAGGGCAGCAACAACCATTTC
CACCACAACAGCCATATCCGCAGCCGCAACCATTTCCATCACAACAACCATATCTGCAGCTGCAACCATTTCCGCAGCCGCAACTA
CCATATCCGCAGCCGCAACTACCATATCCGCAGCCGCAACTACCATATCCGCAGCCGCAACCATTTCGACCACAACAACCATATCC
ACAATCGCAACCACAGTATTCGCAACCACAACAACCAATTTCGCAGCAGCAGCAGCAACAACAACAACAACAACAAAAACAACAAC
AACAACAACAACAACAACAGATCCTTCAACAAATTTTGCAACAACAACTGATTCCATGCAGGGATGTTGTATTGCAACAACACAGC
ATAGCGTATGGAAGCTCACAAGTTTTGCAACAAAGTACTTACCAGCTGGTGCAACAATTGTGTTGTCAGCAGCTGTGGCAGATCCC
CGAGCAGTCGCGGTGCCAAGCCATCCACAATGTTGTTCATGCTATTATTCTGCATCAACAGCAACAACAACAACAACAACAACAAC
AAAAACAACCATTGAGCCAGGTCTCCTTCCAACAGCCTCAACAACAATATCCATCAGGCCAGGGCTCCTTCCAGCCATCTCAGCAA
AACCCACAGGCCCAGGGCTCTGTCCAGCCTCAACAACTGCCCCAGTTTGAGGAAATAAGGAACCTAGCGCTAGAGACGCTACCTGC
AATGTGCAATGTCTATATCCCTCCATATTGCACCATTGCTCCAGTTGGCATCTTCGGTACTAACTGA

α-D5 amino acid 309 aa

MKTFLILALLAIVATTATIAVRVPVPQLQPQNPSQQQPQEQVPLVQQQQFPGQQQPFPPQQPYPQPQPFPSQQPYLQLQPFPQPQL
PYPQPQLPYPQPQLPYPQPQPFRPQQPYPQSQPQYSQPQQPISQQQQQQQQQQKQQQQQQQQQILQQILQQQLIPCRDVVLQQHS
IAYGSSQVLQQSTYQLVQQLCCQQLWQIPEQSRCQAIHNVVHAIILHQQQQQQQQQQKQPLSQVSFQQPQQQYPSGQGSFQPSQQ
NPQAQGSVQPQQLPQFEEIRNLALETLPAMCNVYIPPYCTIAPVGIFGTN.

α-D6 nucleotide 885 bp

ATGAAGACCTTTCTCATCCTTGCCCTCCTTGCTATTGTAGCAACCACCGCCACAATTGCAGTTAGAGTTCCAGTGCCACAATTGCA
GCCACAAAATCCATCTCAGCAACAACCACAAGAGCAAGTTCCATTGGTACAACAACAACAATTTCCAGGGCAGCAACAACCATTTC

CACCACAACAGCCATATCCGCAGCTGCAACCATTTCCATCACAACAACCATATATGCAGCTGCAACCATTTCCGCAGCCGCAACTA
CCATATCCGCAGCCGCAACTACCATATCCGCAGCCGCAACCATTTCGACCACAACAATCATATCCACAACCGCAACCACAGTATTC
GCAACCACAACAACCAATTTCGCAGCAGCAGCAGCAGCAGCAGCAACAACAACAACAACAGATCCTTCAACAAATTTTGCAACAAC
AACTGATTCCATGCAGGGATGTTGTATTGCAACAACACAGCATAGCGCATGGAAGCTCACAAGTTTTGCAACAAAGTACTTACCAG
CTGGTGCAACAATTGTGTTGTCAGCAGCTGTGGCAGATCCCCGAGCAGTCGCGGTGCCAAGCCATCCACAATGTTGTTCATGCTAT
TATTCTGCATCAACAACAACAACAACAACAACAACAACAACAACAACAACAACCGTTGAGCCAGGTCTGCTTCCAACAGTCTCAAC
AACAATATCCATCAGGCCAGGGCTCCTTCCAGCCATCTCAGCAAAACCCACAGGCCCAGGGCTCTGTCCAGCCTCAACAACTGCCC
CAGTTTGAGGAAATAAGGAACCTAGCGCTAGAGACGCTACCTGCAATGTGCAATGTCTATATCCCTCCATATTGCACCATTGCTCC
AGTTGGCATCTTCGGTACTAACTGA

α-D6 amino acid 295 aa

MKTFLILALLAIVATTATIAVRVPVPQLQPQNPSQQQPQEQVPLVQQQQFPGQQQPFPPQQPYPQLQPFPSQQPYMQLQPFPQPQL
PYPQPQLPYPQPQPFRPQQSYPQPQPQYSQPQQPISQQQQQQQQQQQQQQILQQILQQQLIPCRDVVLQQHSIAHGSSQVLQQSTYQ
LVQQLCCQQLWQIPEQSRCQAIHNVVHAIILHQQQQQQQQQQQQQQQPLSQVCFQQSQQQYPSGQGSFQPSQQNPQAQGSVQPQQLP
QFEEIRNLALETLPAMCNVYIPPYCTIAPVGIFGTN.

α-D7 nucleotide 852 bp

ATGAAGACCTTTCTCATCCTTGCCCTCCTTGCTATTGTAGCAACCACCGCCACAATTGCAGTTAGAGTTCCAGTGCCACAATTGCA
GCCACAAAATCCATCTCAGCAACAACCACAAGAGCAAGTTCCATTGGTACAACAACAGCAATTTCCAGGGCAGCAACAACCATTTC
CACCACAACAGCCATATCCGCAGCCGCAACCATTTCCATCACAACAACCATATCTGCAGCTGCAACCATTTCCGCAGCCGCAACTA
CCATATCCGCAGCCGCAACCATTTCGACCACAACAACCATATCCACAACCGCAACCACAGTATTCGCAACCACAACAACCAATTTC
ACAGCAGCAGCAGCAGCAGCAACAACAACAACAACAACAACAACAGATCCTTCAACAAATTTTGCAACAATAACTGATTCCATGCA
GGGATGTTGTATTGCAACAACACAGCATAGCGCATGGAAGCTCACAAGTTTTGCAACAAAGTACTTACCAGCTGGTGCAACAATTG
TGTTGTCAACAGCTGTGGCAGATCCCCGAGCAGTCGCGGTGCCAAGCCATCCACAATGTTGTTCATGCTATTATTCTGCATCAACA
ACAACAACAACAACAACAACCGTTGAGCCAGGTCTGCTTCCAACAGCCTCAACAACAATATCCATCAGGCCAGGGCTCCTTCCAGC
CATCTTAGCAAAACCCACAGGCCCAGGGCTCTGTCCAGCCTCAACAACTGCCCCAGTTTGAGGAAATAAGGAACCTAGCGCTAGAG
ACGCTACCTGCAATGTGCAATGTCTATATCCCTCCATATTGCACCATTGCTCCAGTTGGCATCTTCGGTACTAACTGA

α-D7 amino acid 284 aa

MKTFLILALLAIVATTATIAVRVPVPQLQPQNPSQQQPQEQVPLVQQQQFPGQQQPFPPQQPYPQPQPFPSQQPYLQLQPFPQPQL
PYPQPQPFRPQQPYPQPQPQYSQPQQPISQQQQQQQQQQQQQQQILQQILQQ.LIPCRDVVLQQHSIAHGSSQVLQQSTYQLVQQL
CCQQLWQIPEQSRCQAIHNVVHAIILHQQQQQQQQPLSQVCFQQPQQQYPSGQGSFQPS.QNPQAQGSVQPQQLPQFEEIRNLALE
TLPAMCNVYIPPYCTIAPVGIFGTN.

α-D8 nucleotide 879 bp

ATGAAGACCTTTCTCATCCTTGCCCTCCTTGCTATTGTAGCAACCACCGCCACAATTGCAGTTAGAGTTCCAGTGCCACAATTGCA
GCCACAAAATCCATCTCAGCAACAACCACAAGAGCAAGTTCCATTGGTACAACAACAACAATTTCCAGGGCAGCAACAACCATTTC
CACCACAACAGCCATATCCGCAGCCGCAACCATTTCCATCACAACAACCATATCTGCAGCTGCAACCATTTCCGCAGCCGCAACTA
CCATATCCGCAGCCGCAACTACCATATCCGCAGCCGCAACCATTTCGACCACAACAACCATATCCACAACCGCAACCACAGTATTC
GCAACCACAACAACCAATTTCGCAGCAGCAGCAGCAGCAGCAACAACAACAACAACAACAACAGATCCTTCAACAAATTTTGCAAC
AACAACTGATTCCATGCAGGGATGTTGTATTGCAACAACACAGCATAGCGCATGGAAGCTCACAAGTTTTGCAACAAAGTACTTAC
CAGCTGGTGCAACAATTGTGTTGTCAGCAGCTGTGGCAGATCCCCGAGCAGTCGCGGTGCCAAGCCATCCACAATGTTGTTCATGC
TATTATTCTGCATCAACAACAACAACAACAACAACAACAACAACAACCGTTGAGCCAGGTCTGCTTCCAACAGCCTCAACAACAAT
ATCCATCAGGCCAGGGCTCCTTCCAGCCATCTCAGCAAAACCCACAGGCCCAGGGCTCTGTCCAGCCTCAACAACTGCCCCAGTTT
GAGGAAATAAGGAACCTAGCGCTAGAGACGCTACCTGCAATGTGCAATGTCTATATCCCTCCATATTGCACCATTGCTCCAGTTGG
CATCTTCGGTACTAACTGA

α-D8 amino acid 293 aa

MKTFLILALLAIVATTATIAVRVPVPQLQPQNPSQQQPQEQVPLVQQQQFPGQQQPFPPQQPYPQPQPFPSQQPYLQLQPFPQPQL
PYPQPQLPYPQPQPFRPQQPYPQPQPQYSQPQQPISQQQQQQQQQQQQQQILQQILQQQLIPCRDVVLQQHSIAHGSSQVLQQSTY

QLVQQLCCQQLWQIPEQSRCQAIHNVVHAIILHQQQQQQQQQQQPLSQVCFQQPQQQYPSGQGSFQPSQQNPQAQGSVQPQQLPQF
EEIRNLALETLPAMCNVYIPPYCTIAPVGIFGTN.

α-D9 nucleotide 861 bp

ATGAAGACCTTTCTCATCCTTGCCCTCCTTGCTATTGTAGCAACCACCGCCACAATTGCAGTTAGAGTTCCAGTGCCACAATTGCA
GCCACAAAATCCATCTCAGCAACAACCACAAGAGCAAGTTCCATTGGTACAACAACAGCAATTTCCAGGGCAGCAACAACCATTTC
CACCACAACAACCATATCCGCAGCCGCAACCATTTCCATCACAACAACCATATCTGCAGCTGCAACCATTTCCACAGCCGCAACTA
CCATATCCGCAGCCGCAACTACCATATCCGCAGCCGCAACCATTTCGACCACAACAACCATATCCACAACCGCAACCACAGTATTC
GCAACCACAACAACCAATTTCGCAGCAGCAACAACAACAACAACAACAAATCCTTCAACAAATTTTGCAACAACAACTGATTCCAT
GCAGGGATGTTGTATTGCAACAACACAACATAGCGCATGGAAGGTCACAAGTTTTGCAACAAAGTACTTACCAGCTGGTGCAACAA
TTGTGTTGTCAGCAGCTGTGGCAGATCCCCGAGCAGTCGCGGTGCCAAGCCATCCACAATGTTGTTCATGCTATTATTCTGCATCA
TCATCAACAACAACAACAACAACAACAACCGTTGACCCAGGTCTCCTTCCAACAGCCTCAACAACAATATCCATCAGGCCAGGGCT
CCTTCCAGCCATCTCAGCAAAACCCACAGGCCCAGGGCTCTGTCCAGCCTCAACAACTGCCCCAGTTTGAGGAAATAAGGAACCTA
GCGCTAGAGACGCTACCTGCAATGTGCAATGTCTATATCCCTCCATATTGCACCATTGCTCAAGTTGGCATCTTCGGTACTAACTG
A

α-D9 amino acid 287 aa

MKTFLILALLAIVATTATIAVRVPVPQLQPQNPSQQQPQEQVPLVQQQQFPGQQQPFPPQQPYPQPQPFPSQQPYLQLQPFPQPQL
PYPQPQLPYPQPQPFRPQQPYPQPQPQYSQPQQPISQQQQQQQQQILQQILQQQLIPCRDVVLQQHNIAHGRSQVLQQSTYQLVQQ
LCCQQLWQIPEQSRCQAIHNVVHAIILHHHQQQQQQQQPLTQVSFQQPQQQYPSGQGSFQPSQQNPQAQGSVQPQQLPQFEEIRNL
ALETLPAMCNVYIPPYCTIAQVGIFGTN.

α-D10 nucleotide 870 bp

ATGAAGACCTTTCTCATCCTTGCCCTCCTTGCTATTGTAGCAACCACCGCCACAATTGCAGTTAGAGTTCCAGTGCCACAATTGCA
GCCACAAAATCCATCTCAGCAACAACCACAAGAGCAAGTTCCATTGGTACAACAACAACAATTTCCAGGGCAGCAACAACCATTTC
CACCACAACAGCCATATCCGCAGCTGCAACCATTTCCATCACAACAACCATATATGCAGCTGCAACCATTTCCGCAGCCGCAACTA
CCATATCCGCAGCCGCAACTACCATATCCGCAGCCGCAACCATTTCGACCACAACAATCATATCCACAACCGCAACCACAGTATTC
GCAACCACAACAACCAATTTCGCAGCAGCAGCAACAACAACAACAACAACAACAACAACAACAACAAATCCTTCAACAAATTTTGC
AACAACAACTGATTCCATGCAGGGATGTTGTATTGCAACAACACAACATAGCGCATGGAAGCTCACAAGTTTTGCAACAAAGTACT
TACCAGCTGGTGCAACAATTGTGTTGTCAGCAGCTGCGGTAGATCCCCAAGCAGTCGCGGTGCCAAGCCATCCACAATGTTGTTCA
TGCTATTATTCTGCATCAACAACAACAACAACAACAACCGTTGAGCCAGGTCTCCTTCCAACAGCCTCAACAACAATATCCATCAG
GCCAGGGCTCCTTCCAGCCATCTCAGCAAAACCCACAGGCCCAGGGCTCTGTCCAGCCTCAACAACTGCCCCAGTTTGAGGAAATA
AGGAACCTAGCGCTAGAGACGCTACCTGCAATGTGCAATGTCTATATCCCTCCATATTGCACCATTGCTCAAGTTGGCATCTTTGG
TACTAACTGA

α-D10 amino acid 290 aa

MKTFLILALLAIVATTATIAVRVPVPQLQPQNPSQQQPQEQVPLVQQQQFPGQQQPFPPQQPYPQLQPFPSQQPYMQLQPFPQPQL
PYPQPQLPYPQPQPFRPQQSYPQPQPQYSQPQQPISQQQQQQQQQQQQQQQILQQILQQQLIPCRDVVLQQHNIAHGSSQVLQQST
YQLVQQLCCQQLR.IPKQSRCQAIHNVVHAIILHQQQQQQQPLSQVSFQQPQQQYPSGQGSFQPSQQNPQAQGSVQPQQLPQFEEI
RNLALETLPAMCNVYIPPYCTIAQVGIFGTN.

α-D11 nucleotide 1146 bp

ATGAAGACCTTTCTCATCCTTGCCCTCCTTGCTATTGTAGCAACCACCGCCACAATTGCAGTTAGAGTTCCAGTGCCACAATTGCA
GCCACAAAATCCATCTCAGCAACAGCCACAAGAGCAAGTTCCATTGGTACAACAACAACAATTTCCAGGGCAGCAACAACCATTTC
CACCACAACAGCCATATCCGCGGCCGCAACCATTTTCATCACAACAACCATATCTGCAGCTGCAACCATTTCCGCAGCCGCAACTA
CCATATCCGCAGCCGCAACCATTTCGACCACAACAACCATATCCACAACCGCAACCACAGTATTAGCAACCACAACAACCAATTTC
GCAGCTGCAGCAGCAACAACAACAACAATAGCAACAACAACAGCAACAAAAACAGCAGCAACAACAACAGCAGCAGCAACAACAAC
AACAACAGCAACAACACCAGCAACAACAGCACCAACAGCAGTAGCAGCAGCAGCAACAACAACAACAACAACAACAACAGCAGCAG
CAGCAGCAGCAGCAGCAGCAGCAGCAGCAGCAACAACAACAACAACAACAACAACAACAACAACAacACAACAACAACAAACAGCA
GCAGCAGCAGCAGCAGCAGCAACAACAACAACAACAATAACAACAACAAATTTTGCAACAACAACAACAACAAATTTTGCAAC
AACAACAACAACAACAAATTTTGCAACAACAACTGATTCCATGCAGGGATGTTGTATTGCAACAACACAACATAGCGCATGGAAGC
TCACAAGTTTTGCAACAAAGTACTTACCAGCTGGTGTAACAATTGTGTTGTCAGCAGCTGTGGCAGATTCCCGAGCAGTCGCGGTG

CCAAGCCATCCACAATGTTGTTCATGCTATTATTCTGCATCAACAACAACAACAACCGTCGAGCCAGGTCTCCTTCCAACAGCCTC
AACAACAATATCCATCAGGCCAAGGCTCCTTCCAGCCATCTCTGCAAAACCCACAGGCCCATGGCTCTGTCCAGCCTCAACAACTG
CCCCAGTTTGAGGAAATAAGGAACCTAGCGCTACAGACGCTACCTGCAATGTGCAATGTCTACATCCCTCCATATTGCACCATTGC
TCCATTTGGCATCTTCGGTACTAACTGA

α-D11 amino acid 382 aa

MKTFLILALLAIVATTATIAVRVPVPQLQPQNPSQQQPQEQVPLVQQQQFPGQQQPFPPQQPYPRPQPFSSQQPYLQLQPFPQPQL
PYPQPQPFRPQQPYPQPQPQY.QPQQPISQLQQQQQQ.QQQQQQKQQQQQQQQQQQQQQQQHQQQQHQQQ.QQQQQQQQQQQQQQ
QQQQQQQQQQQQQQQQQQQQQHNNNKQQQQQQQQQQQQQQQ.QQQILQQQQQQILQQQQQQQILQQQLIPCRDVVLQQHNIAHGS
SQVLQQSTYQLV.QLCCQQLWQIPEQSRCQAIHNVVHAIILHQQQQQPSSQVSFQQPQQQYPSGQGSFQPSLQNPQAHGSVQPQQL
PQFEEIRNLALQTLPAMCNVYIPPYCTIAPFGIFGTN.

α-D12 nucleotide 849 bp

ATGAAGACCTTTCTCATCCTAGCCCTCCTTGCTATCGTGGCGACCACCGCCACAAGTGCAGTTAGAGTTCCAGTGCCACAATTGCA
GCCGCAAAATCCATCTCAACAACAACCACAAGAGCAAGTTCCATTGATGCAACAACAACAACAATTTCCAGGGCAGCAAGAACAAT
TTCCACCACAACAGCCATATCCGCATCAGCAACCATTTCCATCACAACAACCATATCCGCAGCCGCAACCATTTCCGCCACAACTA
CCATATCCGCAGACGCAACCATTTCCACCACAACAACCATATCCACAACCGCAACCACAGTATCCGCAACCACAACAACCAATTTC
GCAGCAACAAGCACAACAACAACAACAACAACAACAAATCCTTCAACAAATTCTGCAACAACAACTGATTCCATGCAGGGATGTTG
TCTTGCAACAACACAACATAGCGCATGCAAGCTCACAAGTATTGCAACAAAGTAGTTACCAACAGTTGCAACAATTATGTTGTCAG
CAACTGTTTCAGATCCCCGAGCAGTCGCGGTGCCAAGCCATCCACAATGTCGTTCATGCTATTATTCTGCATCATCATCAACAACA
ACAACAACAACCGTCGAGCCAGGTCTCCTACCAGCAGCCTCAGGAACAATATCCATCAGGCCAGGGCTCCTTCCAGTCATCTCAGC
AAAACCCACAGGCCCAGGGCTCTGTCCAGCCTCAACAACTGCCCCAGTTCCAGGAAATAAGGAACTTAGCGCTGCAGACGCTGCCA
GCAATGTGCAATGTCTACATCCCTCCATATTGCTCGACCACCATTGCGCCATTTGGCATCTTCGGTACTAACTGA

α-D12 amino acid 283 aa

MKTFLILALLAIVATTATSAVRVPVPQLQPQNPSQQQPQEQVPLMQQQQQFPGQQEQFPPQQPYPHQQPFPSQQPYPQPQPFPPQL
PYPQTQPFPPQQPYPQPQPQYPQPQQPISQQQAQQQQQQQQILQQILQQQLIPCRDVVLQQHNIAHASSQVLQQSSYQQLQQLCCQ
QLFQIPEQSRCQAIHNVVHAIILHHHQQQQQQPSSQVSYQQPQEQYPSGQGSFQSSQQNPQAQGSVQPQQLPQFQEIRNLALQTLP
AMCNVYIPPYCSTTIAPFGIFGTN.

Note: --- represents deletion in the sequence that had resulted in frameshift mutation. In the sequences presented here, the frameshift has been manually corrected to provide a putative sequence without the deletion.

. in the middle of the sequences represents premature stop codon caused by nucleotide substitution.

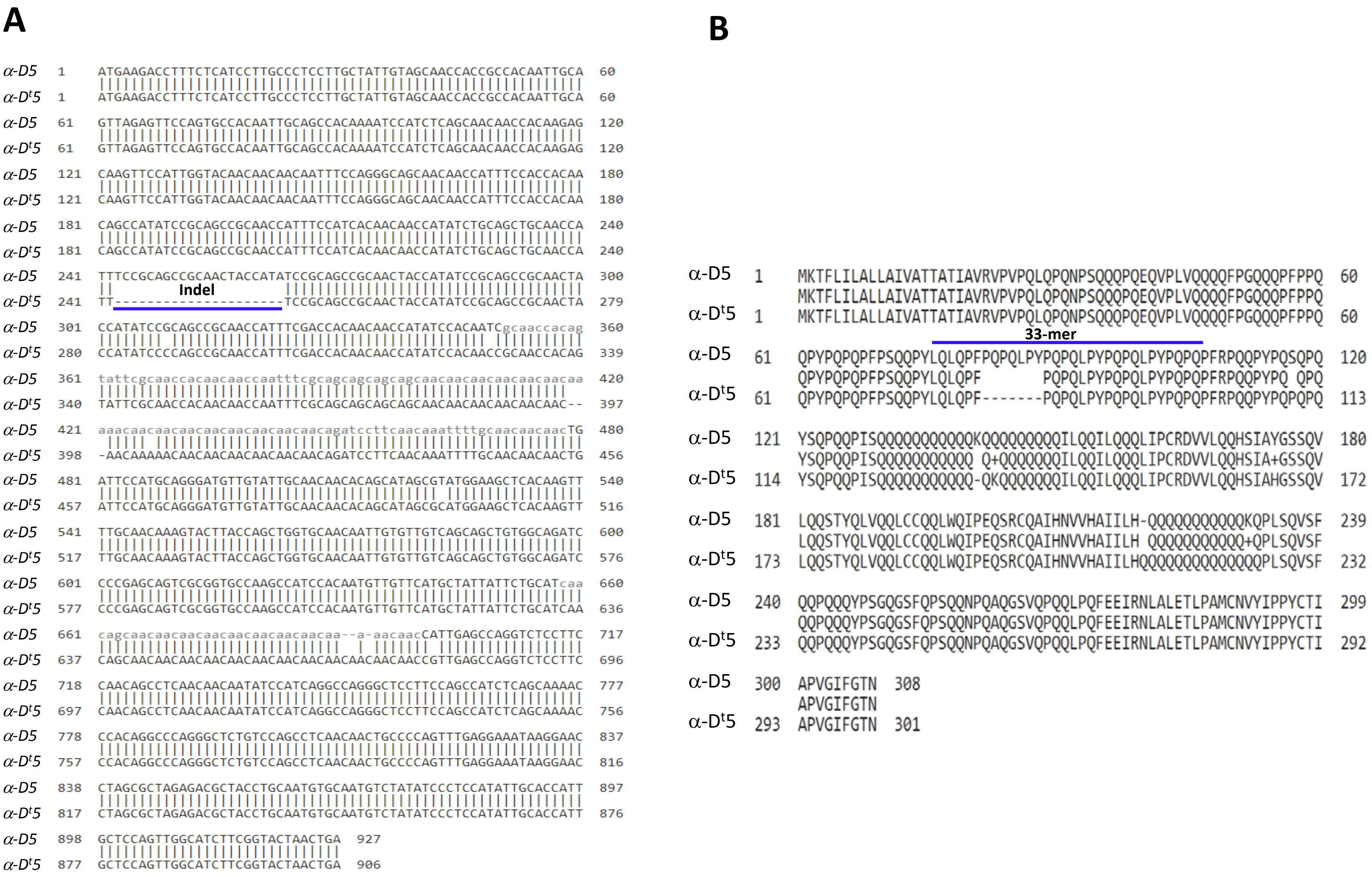

Figure S5. Alignments of nucleotide and amino acid sequences of α-D5 and α-D[t]5 homologues
The indel in the nucleotide sequence and the corresponding 33-mer peptide region in the protein are indicated
